# Nucleus-cytoskeleton communication impacts on OCT4-chromatin interactions in embryonic stem cells

**DOI:** 10.1186/s12915-021-01207-w

**Published:** 2022-01-07

**Authors:** Juan José Romero, María Cecilia De Rossi, Camila Oses, Camila Vázquez Echegaray, Paula Verneri, Marcos Francia, Alejandra Guberman, Valeria Levi

**Affiliations:** 1 Instituto de Química Biológica de la Facultad de Ciencias Exactas y Naturales (IQUIBICEN), CONICET-Universidad de Buenos Aires, Facultad de Ciencias Exactas y Naturales, C1428EGA Buenos Aires, Argentina; 2 Departamento de Fisiología, Biología Molecular y Celular, Facultad de Ciencias Exactas y Naturales, Universidad de Buenos Aires, C1428EGA Buenos Aires, Argentina; 3 Departamento de Química Biológica, Facultad de Ciencias Exactas y Naturales, Universidad de Buenos Aires, C1428EGA Buenos Aires, Argentina

**Keywords:** Embryonic stem cells, Cytoskeleton, Nuclear morphology, OCT4, Transcription factors dynamics, Fluorescence correlation spectroscopy

## Abstract

**Background:**

The cytoskeleton is a key component of the system responsible for transmitting mechanical cues from the cellular environment to the nucleus, where they trigger downstream responses. This communication is particularly relevant in embryonic stem (ES) cells since forces can regulate cell fate and guide developmental processes. However, little is known regarding cytoskeleton organization in ES cells, and thus, relevant aspects of nuclear-cytoskeletal interactions remain elusive.

**Results:**

We explored the three-dimensional distribution of the cytoskeleton in live ES cells and show that these filaments affect the shape of the nucleus. Next, we evaluated if cytoskeletal components indirectly modulate the binding of the pluripotency transcription factor OCT4 to chromatin targets. We show that actin depolymerization triggers OCT4 binding to chromatin sites whereas vimentin disruption produces the opposite effect. In contrast to actin, vimentin contributes to the preservation of OCT4-chromatin interactions and, consequently, may have a pro-stemness role.

**Conclusions:**

Our results suggest roles of components of the cytoskeleton in shaping the nucleus of ES cells, influencing the interactions of the transcription factor OCT4 with the chromatin and potentially affecting pluripotency and cell fate.

**Supplementary Information:**

The online version contains supplementary material available at 10.1186/s12915-021-01207-w.

## Background

Cells are continuously exposed to forces that propagate to their interior through the cytoskeleton, a network of interconnected biopolymers and crosslinker molecules in constant remodeling. This filament network is also physically connected to the cell nucleus through the LINC (linker of nucleoskeleton and cytoskeleton) complex which main components KASH and SUN interact with the cytoskeleton and the nuclear intermediate filaments lamins, respectively [1], constituting a direct mechanism for communicating mechanical signals to the nucleus interior [2].

Forces applied to cells may affect the shape and position of the nucleus [3, 4] and modulate diverse aspects of its function including chromatin organization and gene expression programs [3, 5]. This relation is particularly relevant in stem cells since forces can regulate cell fate and guide developmental processes [6, 7]. In this direction, it was demonstrated that the elasticity of the cell matrix impacts on lineage specification [8] opening the possibility of manipulating cell fate decisions through the rational design of substrates for in vitro differentiation protocols [9]. However, important aspects of the cytoskeleton organization in embryonic stem (ES) cells remain elusive and thus its role in pluripotency maintenance and differentiation is not completely understood. Relevantly, disruption or alterations of cytoskeleton components as actin [10, 11] or vimentin intermediate filaments [12] affect cell fate decisions emphasizing the necessity of a three-dimensional (3D) description of the cytoskeleton organization in live ES cells.

A previous work claimed that the cytoskeleton of ES cells was poorly organized from a comparative analysis of the distribution of cytoskeletal proteins in single-plane images of immunolabeled stem cells and fibroblasts [13]. However, ES cells are essentially three-dimensional objects, and thus, it is expected that single-plane observations are not sufficient to capture the complexity of the cytoskeleton. Moreover, the fixation of cells required for the immunostaining procedure can modify the 3D architecture and organization of intracellular components including the cytoskeleton [14, 15].

Here, we study the 3D distribution of different cytoskeletal filaments in live ES cells since the role of the cytoskeleton on gene expression regulation is poorly understood in the pluripotent state compared to its role during differentiation. We also evaluate if the different cytoskeleton components modulate the nuclear shape and use fluorescence correlation spectroscopy (FCS) to test if these networks affect the dynamical organization of OCT4, a key pluripotency transcription factor (TF). Together, OCT4, SOX2, and NANOG constitute the core of pluripotency defining a regulatory network that induce genes necessary to preserve pluripotency and repress those involved in differentiation [16].

Our study reveals new features of the 3D cytoskeleton organization in live ES cells that were hidden in single-plane images of fixed ES cells. We also show that alterations of either the actin or the intermediate filament vimentin networks affect the nuclear morphology and impact on OCT4-chromatin interactions in contrast to alterations of the microtubule network that does not modify these properties. These results highlight the role of specific cytoskeletal components in modulating the shape of the nucleus of ES cells and unveil its impact on the dynamical organization of a main pluripotency TF. We hypothesize that these early changes of OCT4-chromatin interactions may produce, at a longer time scale, modifications in gene expression ultimately affecting cell fate decisions.

## Results

### Three-dimensional organization of the cytoskeleton of mouse ES cells

In order to examine the 3D organization of the cytoskeleton in naïve ES cells, we acquired confocal z-stacks of live cells co-expressing cytoskeleton-related proteins fused to green fluorescent proteins (GFP or EGFP) and the histone H2B fused to the red fluorescent protein mCherry (H2B-mCherry) to visualize the cell nucleus simultaneously. To account for our observations, we report in each case the percentage of transfected cells that present a certain cytoskeletal feature (% of *n*_cells_). We should emphasize that this percentages do not correspond to the frequency of these features in ES cells because they also depend on other factors including the expression levels of the cytoskeletal proteins (which determines the signal/noise ratio of the specific structure) and instrumental factors. Particularly, the photobleaching caused during the z-stack confocal imaging and/or the scattering produced by intracellular structures may prevent the observation of a certain cytoskeleton feature in some planes of a given cell.

We first observed the microtubules using a plasmid encoding the GFP-tagged microtubule-binding domain of ensconsin (EMTB-3xGFP) [17]. Fluorescent microtubule-associated proteins are excellent tools to label microtubules in living cells since they do not alter the network organization substantially [18, 19].

**Fig. 1.**
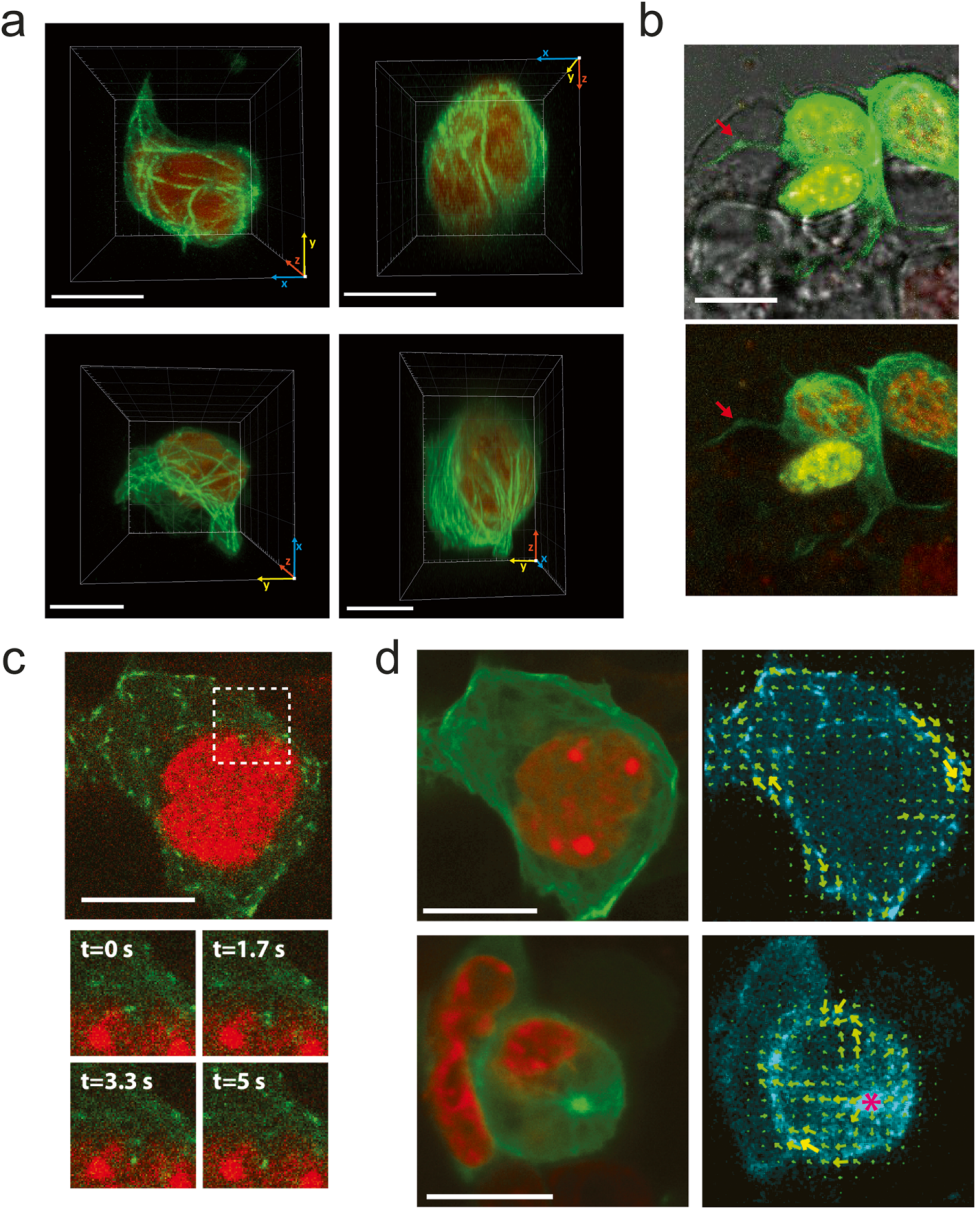
ES cells present an atypical organization of microtubules in interphase with nucleation centers. 3D confocal images of ES cells co-transfected with H2B-mCherry (red) and EMTB-3xGFP (green) (**A**, **B**) or EB3-GFP (**C**, **D)**. **A** 3D reconstruction of representative cells showing the organization of the microtubule network (Additional file 1: Supplementary Video S1 and Additional file 2: Supplementary Video S2). Other examples of 3D reconstructions can be found in Additional file 14: Supplementary Fig. S6. **B** Maximum intensity projection image merged with the transmission image collected at a single plane of the z-stack; the arrow points to a microtubule-enriched cellular protrusion extending to another cell. The top image was digitally saturated to facilitate the observation of the protrusion. **C** Representative, single-plane image of a cell expressing EB3-GFP (top); zoom-in images of the cell region delimited by the dashed square at four different frames of the time-lapse movie showing an EB3 comet in close contact to the nucleus (bottom). **D** Maximum intensity projection images obtained from a 100-image stack obtained during a time-lapse experiment lasting 166.6 s (left); flow maps of the EB3-GFP comets (right). The pink asterisk shows a microtubule nucleation center from which EB3 comets emanate. Scale bars: 10 μm. Other examples of the analysis of EB3 comets can be found in Additional file 15: Supplementary Video S8

Figure 1a shows representative 3D images of the cells with microtubules that spread in the cytoplasm (Additional file 1: Supplementary Video S1 and Additional file 2: Supplementary Video S2) in clear contrast to the disorganized tubulin distribution previously suggested from single-plane images of immunolabeled ES cells [20]. Nevertheless, the network does not seem to present the typical radial-like distribution observed in many somatic cells [21].

In some cases (12% of *n*_cells_ = 93), we detected microtubule-enriched protrusions that extend to other cells (Fig. 1b). Similar protrusions were also observed visualizing the plasma membrane by transfection of mem-mCherry (Additional file 3: Supplementary Fig. S1) and resemble those observed by scanning electron microscopy [22].

To get further insights in the organization of microtubules, we transfected ES cells with the end-binding protein EB3 fused to GFP (EB3-GFP) that associates to the growing tip of microtubules [23, 24], and acquired time-lapse confocal images at certain optical sections of the cells (Fig. 1c, d). We recovered the trajectories of the EB3-GFP comets and analyzed these trajectories to obtain a flow map of EB3 comets as described in the “Methods” section. Representative movies obtained in these imaging experiments show that, while some EB3-GFP comets point in every direction (32% of *n*_cells_ = 25, Fig. 1d top panel and Additional file 4: Supplementary Video S3), others seem to irradiate from specific sites in the cytoplasm (44% of *n*_cells_ = 25, Fig. 1d bottom panel and Additional file 5: Supplementary Video S4) suggesting the presence of microtubule-organizing centers (MTOCs). Although our experiments do not allow identifying the nature of these MTOCs, a previous work described centrioles in electron microscopy images of ES cells [22]. However, another report suggests that centrioles first appear in mouse embryos after the 64-cell stage in trophectoderm cells and thus they are absent in the inner cell mass from which ES cells are derived [25] suggesting that the observed nucleation centers might be acentrosomal MTOCs. These structures were also observed in other cell types (reviewed in [26]) such as mammalian oocytes that lack centriole pairs [27] and their spindle microtubules are nucleated by multiple acentrosomal MTOCs [28].

Interestingly, some movies show EB3-GFP comets in close contact with the nucleus and other comets that seem to be *poking* it (24%, *n*_cells_ = 25, Fig. 1c and Additional file 6: Supplementary Video S5) suggesting that they locally transmit pushing forces to this organelle. Relevantly, microtubules are usually involved in rotating and positioning the nucleus [29–31] and it has been previously proposed that *poking* microtubules produce nucleus wriggling that contribute to position this organelle [32].

**Fig. 2.**
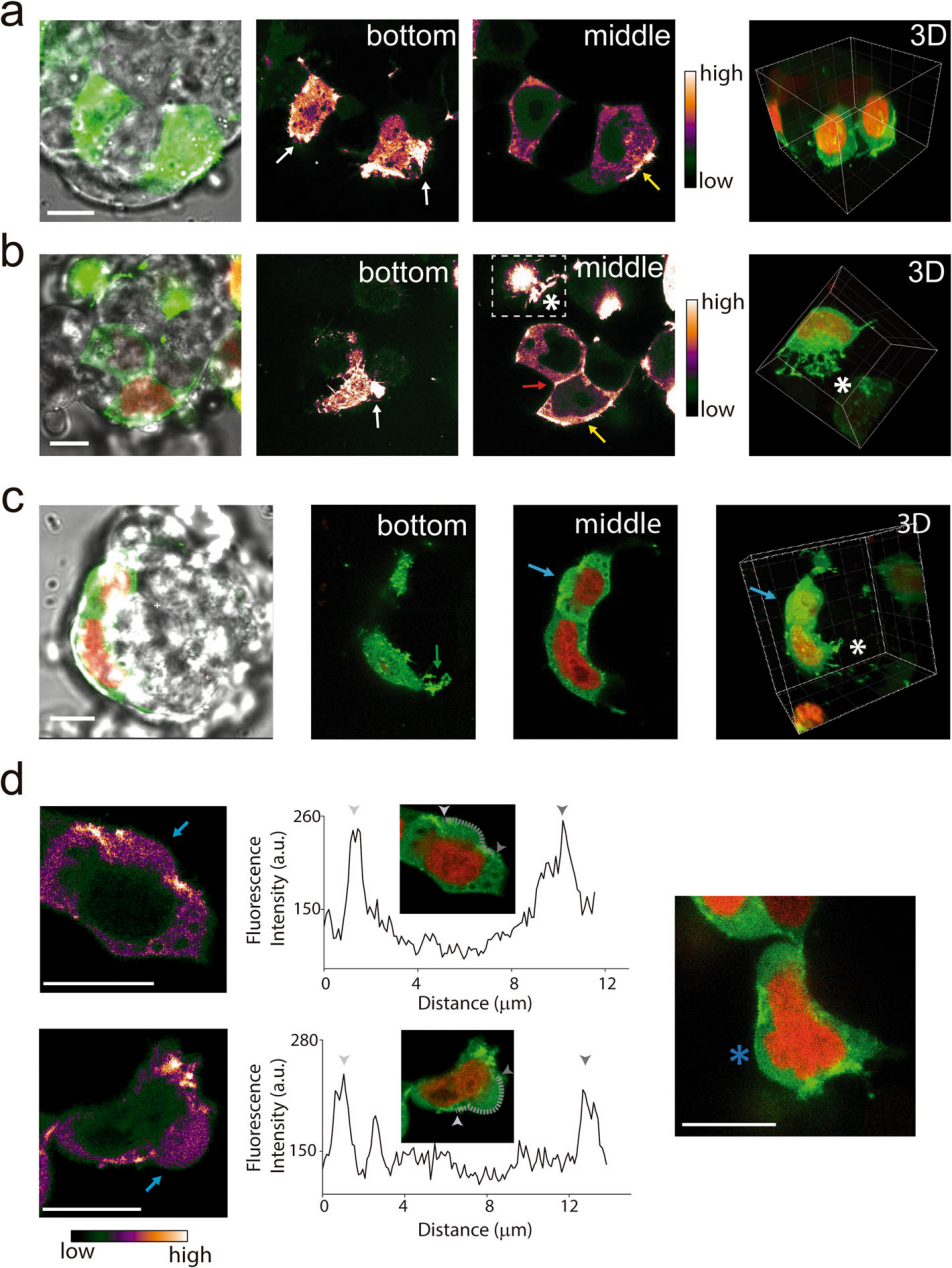
Actin preferentially concentrates in filopodia, cell-cell, and cell-substrate surfaces of ES cells. **A**–**C** Representative images of cells expressing EGFP-actin (green) and H2B-mCherry (red) exemplifying those key features of actin organization described in the text. Merge images (transmission, green and red channels) obtained at specific optical planes allow identifying the relative positions of the cells within colonies (left panels). Images obtained at specific planes of the cells as detailed in each case (central panels, in pseudocolor scale) and 3D reconstructions of the green and red channels images (right panels). White arrows point to actin-enriched structures in contact with the substrate, and the red and green arrows point to actin enrichments at cell-cell contacts and filopodia linking the cell to the substrate, respectively. The yellow arrows point to regions facing the extracellular milieu of those cells at the colony border. The white asterisks indicate actin-enriched filopodia extending from the cell showed in the dotted rectangle to a neighboring cell. **D** Representative examples of cells showing membrane blebs (light blue arrows) (left panels, pseudocolored images). Intensity profiles along the membrane borders (dotted, gray lines in the insets). Grey triangles show the initial and final positions of the lines along the blebs. Bleb formation may be also accompanied by changes in the nucleus shape (right panel, blue asterisk). The white asterisk indicates actin-enriched filopodia (Scale bars: 10 μm). Other examples of EGFP-actin fluorescence intensity along membrane blebs can be found in Additional file 16: Supplementary Fig. S7

We also explored the 3D distribution of actin in ES cells. Figure 2a shows that EGFP-actin displayed a diffuse organization in the cell cytoplasm in line with previous low-resolution and single-plane images of actin immunostaining in ES cells [13] and in contrast to the clear filamentous structures observed in many somatic cells [33]. Nevertheless, our 3D live imaging experiments revealed other aspects of actin organization in ES cells.

Figure 2a–c shows actin-enriched structures in contact with the substrate (white arrows) as expected from the involvement of this cytoskeleton component in cell-substrate interactions [34]. Previous works in human induced pluripotent stem (iPS) cells described an actin fence formed by thick fibers that organize parallel to the colony borders tightly packing the colony [35, 36]. Despite we could not observe these fences in our images of mouse ES cells, actin concentrated in close proximity to membranes facing the external milieu (68% of *n*_*peripherical cells*_ = 88, yellow arrows in Fig. 2a, b) with short actin-enriched filopodia linking cells of the colony borders to the substrate (83% of *n*_*peripherical cells*_ = 88, green arrow in Fig. 2c); these filopodia are also present in inner cells but in a lower proportion (41% of *n*_*inner cells*_ = 107). This different organization of actin in mouse and human pluripotent stem cells could also explain the comparatively higher cell-extracellular matrix traction forces generated by human ES cells [37]. Human and mouse pluripotent stem cell colonies also differ in their morphology and their substrate requirement in feeder-free conditions [38–40] evidencing the differences in cell-cell and cell-substrate interactions between these species.

Figure 2b, c shows that EGFP-actin also concentrates at cell-cell contacts (65% of *n*_cells_ = 195, red arrow) where it probably interacts with cell-adhesion molecules [41]. We observed actin-enriched structures that protrude from one cell and grasp the dorsal membrane of a neighboring cell (23% of *n*_cells_ = 195, Fig. 2b, c white asterisks), suggesting that they may contribute to keep cells tight together within the colony. These protrusions also resemble those filopodia involved in the control of changes in cell shape during compaction of early mouse embryos [42].

 Furthermore, we observed membrane blebs in some cells located at the colony boundaries (13% of *n*_*peripherical cells*_ = 88, Fig. 2c, d, light-blue arrows ); relevantly, the fluorescence intensity of EGFP-actin and therefore the actin concentration at these blebs seems to be lower. In a recent work, super-resolution microscopy showed that cortical actin in fixed ES cells organizes as a low density and isotropic meshwork that does not depend on myosin II activity [43]. In this context, we can hypothesize that some cells apply forces to their neighbors—through those filopodia described above and/or lateral forces—which may release the tension by locally disrupting the sparse meshwork of cortical actin and thus generating a bleb. Similar blebs produced by a breakage of the actin cortex were observed when adherent cells detach from their substrate [44]. Additionally, blebbing increases during the exit from naïve pluripotency, prior to cell spreading in mouse ES cells [45, 46]. In some cases, blebs were accompanied by deformation of the nucleus illustrating how forces applied to ES cells may also shape the nucleus (Fig. 2d).

We next focused our attention on vimentin, one of the most studied intermediate filaments in many cell lines due to its key role in diverse cell processes such as migration [47]. Previous evidence also suggests that vimentin is relevant for differentiation of ES cells [12].

Based on immunofluorescence assays, Ginis et al. [38] claimed that this protein was undetectable in mouse ES cells whereas Boraas et al. [13] observed that it is expressed at relatively low levels. These apparently contradictory reports led us to explore vimentin expression by transcriptomic and proteomic data mining (Additional file 7: Supplementary Table S1, [48–55]).

The analysis of RNA-seq and microarray data showed that vimentin is expressed at different stages of the developing embryo (Additional file 8: Supplementary Fig. S2a), in ES cells and in other types of stem cells (Additional file 8: Supplementary Fig. S2b). Although vimentin mRNA levels increase during most differentiation processes (Additional file 8: Supplementary Fig. S2c), it is downregulated during epiblast-like cells (EpiLCs) differentiation and, remarkably, it is still detectable after this downregulation (Additional file 8: Supplementary Fig. S2d). We also found that vimentin expression is similar in ES cells and iPS cells and is higher in mouse embryonic fibroblasts (MEFs), which are the corresponding parental differentiated cells (Additional file 8: Supplementary Fig. S2e), agreeing with Boraas et al. [13]. Moreover, RNA-seq and proteomic data analyses revealed that vimentin expression is downregulated during the reprogramming process (Additional file 8: Supplementary Fig. S2f). Altogether, these data demonstrate that vimentin is expressed in pluripotent stem cells.

**Fig. 3.**
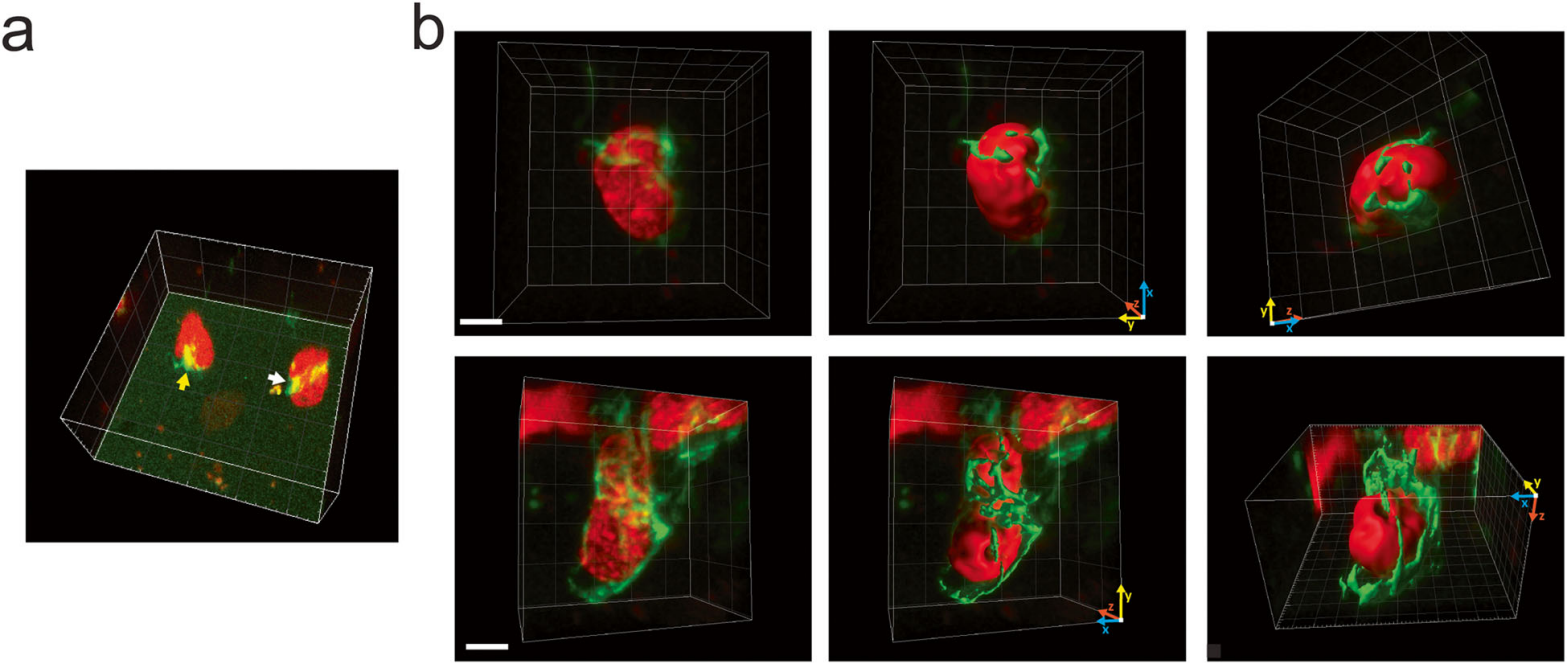
Vimentin intermediate filaments associate with the nucleus of interphase ES cells. Representative 3D images of ES cells expressing H2B-mCherry (red) and GFP-vimentin (green). **A** Yellow and white arrows point to vimentin knot and ring-like structures, respectively. **B** Images of the cells expressing relatively low (top panels) or intermediate (bottom panels) levels of GFP-vimentin showing a close association between vimentin structures and the nucleus (Additional file 9: Supplementary Video S6 and Additional file 10: Supplementary Video S7). These images were segmented as described in Methods (middle and right panels) to facilitate their observation (scale bars: 5 μm)

We next analyzed through confocal imaging the distribution of vimentin in live ES cells transfected with a plasmid encoding GFP-vimentin and observed filaments close to or surrounding the cell nucleus (Fig. 3, Additional file 9: Supplementary Video S6 and Additional file 10: Supplementary Video S7). Relevantly, we observed a close association of GFP-vimentin with the nucleus even in those cells presenting relatively low expression levels of the fluorescent protein (Fig. 3a) suggesting that this association is not an aberrant distribution due to overexpression of the fusion protein. Notably, vimentin is organized in knots and ring-like structures around the nucleus in 33% and 37% of the studied cells, respectively (*n*_cells_ = 70, Fig. 3b). These last structures evoke transient vimentin rings observed during both the initial stages of cell spreading and the detachment that precedes mitosis [56], and thus, we speculate that they might represent a frequent structure in cells with low spreading. Despite the vimentin network cannot generate forces per se, those vimentin-containing rings described before may be involved in nuclear shaping [56] supporting their contribution in transmitting mechanical stimuli to the nucleus. Taken together, the close association between vimentin intermediate filaments and the nucleus of living ES cells suggest that these filaments are involved in mechanical communication to the nucleus.

### Modulation of the nuclear shape of ES cells by the cytoskeletal networks

In the previous section, we analyzed the 3D distribution of different cytoskeleton components and examined their organization in relation to the cell nucleus. Several works showed that internal and external forces may affect the nuclear volume and its morphology in a cell-type dependent manner (e.g., [57, 58]). In this sense, the morphology of the nucleus changes in mechanically stressed situations for example, during migration [59] and cell spreading [58].

We next asked if the cytoskeleton components studied in the previous section mechanically communicate with the ES cell nucleus. With this idea, we analyzed the nuclear morphology after disturbing each of these cytoskeletal networks. We highlight that these experiments provide indirect, qualitative information regarding the involvement of different cytoskeletal filaments in mechanotransmission to the nucleus (as defined in [60]) but does not allow the quantification of the mechanical properties of the cytoskeleton of ES cells or the forces applied to the nucleus.

**Fig. 4.**
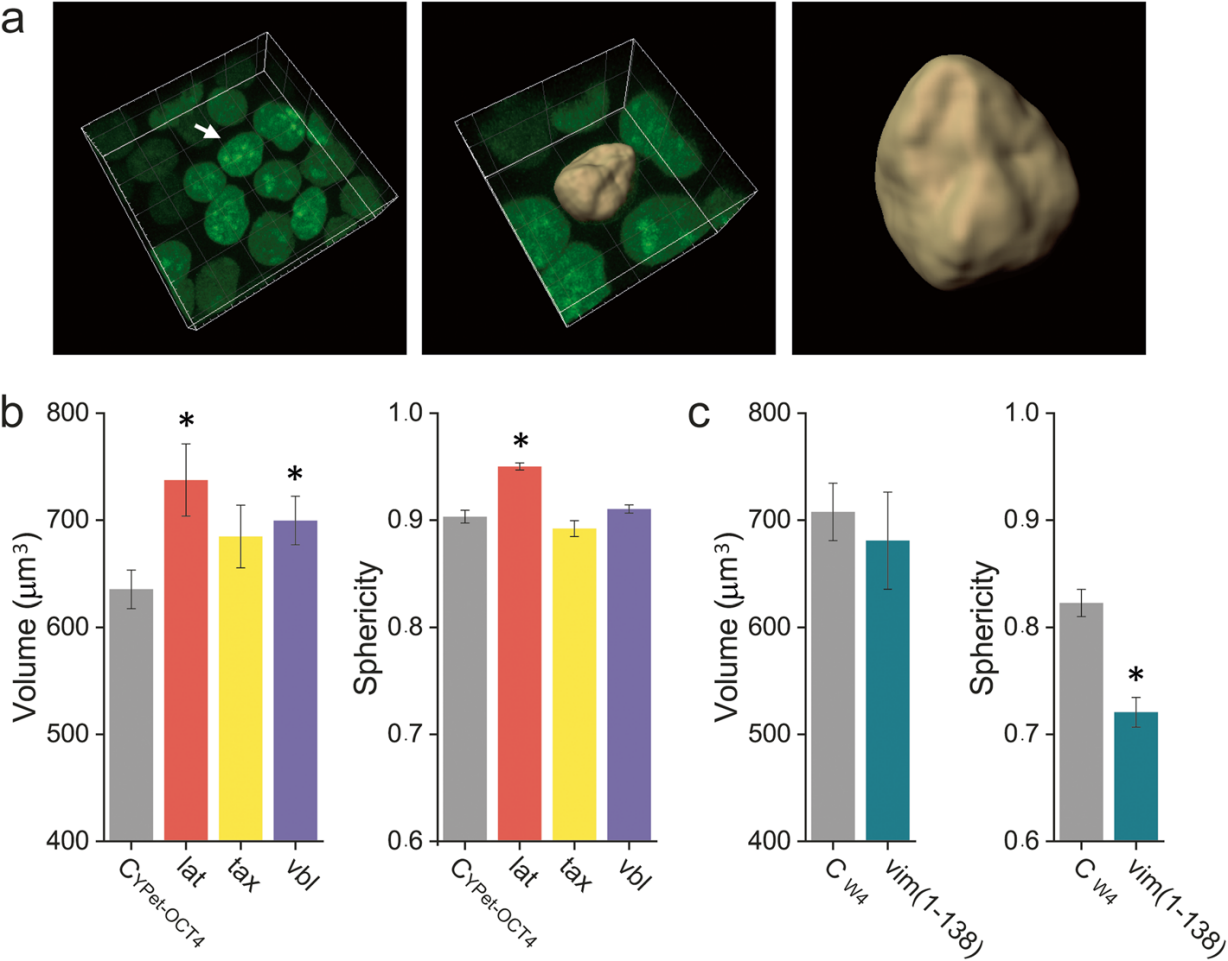
The cytoskeletal networks regulate the nuclear morphology of ES cells. **A** Representative 3D image of a region of an YPet-OCT4 ES cell colony. The nuclei images were segmented to quantify the volume of each nucleus and its sphericity as described in the “Methods” section. **B** Quantification of the nuclei volume and sphericity in untreated YPet-OCT4 ES cells (C_YPet-OCT4_) or YPet-OCT4 ES cells incubated with latrunculin-B (lat), taxol (tax), or vinblastine (vbl). **C** Similar quantifications performed in W4 ES cells only expressing H2B-mCherry (C_W4_) or co-expressing H2B-mCherry and the dominant negative vimentin mutant GFP-(vim(1-138)). The data is presented as median ± SE for each experimental condition (*n*_CYPet-OCT4_ = 165; *n*_lat_ = 55; *n*_tax_ = 58; *n*_vbl_ = 146; *n*_CW4_ = 32; *n*_vim(1-138)_ = 55). Please, notice that the values measured in C_YPet-OCT4_ and C_W4_ conditions could be different due to the different emission spectra of the fluorescent protein used in each case. Asterisks indicate significant differences (*p* < 0.05) with respect to that obtained for the corresponding control cells. Raw data can be found in Additional file 17: Supplementary Table S2

For these experiments, we used the YPet-OCT4 ES cell line previously generated by our group [61] that expresses the pluripotency TF OCT4 fused to the fluorescent protein YPet in a docycycline-inducible manner. We have previously shown that this cell line preserves relevant properties of the parental cell line including the morphology of the cells and colonies, normal cell cycle and the expression profile of pluripotency markers [61], and it was also observed that the YPet tag does not affect the subcellular localization of OCT4 [62]. Additionally, the YPet-OCT4 fusion protein is functional since it rescues pluripotency of inducible OCT4 knockout ES cells and presents genome-wide binding profiles similar to those of the endogenous TF [63]. The expression of YPet-OCT4 allows visualizing every nucleus in a colony (Fig. 4a). We segmented nuclei images and quantified their volume and sphericity; this last parameter approaches a value of one when the nucleus becomes more spherical and decreases as the nucleus is deformed.

Depolymerization of actin filaments by treating ES cells with latrunculin-B drastically altered the colony morphology (Additional file 11: Supplementary Fig. S3); ES cells rounded-up and detached from each other as expected from the involvement of actin in cell-cell and cell-substrate adhesions.

The abrupt change in cell shape produced by latrunculin-B treatment was accompanied by an increase in the nuclear volume (Fig. 4b). This result could be explained considering that actin produces and/or transmits compressive forces to the nucleus which relaxes after depolymerization of these filaments leading to an increase in its volume. In line with this hypothesis, Kim et al. [57] proposed that actin—and also the microtubule network—compress the nucleus in MEFs. Nevertheless, we should emphasize that this volume change represents a ~ 6% increment in the nucleus radius if we assume the volume to scale with the radius cubed.

We also observed that the nuclei sphericity significantly increased after latrunculin-B treatment (Fig. 4b) suggesting a coupling between cell and nuclear morphologies in mouse ES cells. In line with this statement, previous observations in fibroblasts proposed a similar coupling with round cells deriving in round nuclei and well-spread cells resulting in flat nuclei [58].

To study the impact of microtubules, we first depolymerized them using nocodazole, but ES cell colonies detached from the coverslip after the treatment (Additional file 11: Supplementary Fig. S3). Therefore, we followed an alternative procedure and only disturbed the microtubule network instead of depolymerizing it.

First, we treated the cells with paclitaxel (also referred to as taxol) that promotes the assembly of these filaments slowing down their dynamical instability. This drug produces a reduction of microtubule stiffness in vitro [64, 65] and eliminates the nuclear wriggling produced by poking microtubules [32]. These previous reports led us to speculate that this drug may also produce a mechanical imbalance of the microtubule network of ES cells. We observed that the morphology of the colony was preserved after taxol treatment (Additional file 11: Supplementary Fig. S3) and neither the nuclear volume nor its sphericity significantly changed after this treatment (Fig. 4b). In addition, we analyzed the effects of vinblastine treatment; low concentrations of this drug stabilize microtubules by capping their plus-ends thus arresting their polymerization and depolymerization dynamics [66]. The morphology of the colony was also preserved after vinblastine treatment (Additional file 11: Supplementary Fig. S3) whereas nuclei slightly increased their volumes (~ 3 % increment in the nucleus radius) but did not significantly change their sphericity (Fig. 4b). Taken together, these results suggest that the microtubule network is not a key player in defining the nuclear shape of ES cells.

Finally, we studied the morphology of the nucleus in the parental W4 ES cells transfected with H2B-mCherry and a dominant negative vimentin mutant (vim 1-138) fused to GFP; this fluorescently tagged mutant disrupts vimentin filaments [67]. We should highlight that, in contrast to the relatively fast drug-treatments followed to disrupt microtubules and actin networks, the expression of the mutant vimentin requires a longer period of time. To our knowledge, there are no other methods to selectively disrupt this intermediate filament network. Thus, we cannot rule out that some of the effects observed in these experiments may be indirectly related to the vimentin network disruption. The morphology of the colony was also preserved after transfection of this truncated vimentin (Additional file 11: Supplementary Fig. S3). Figure 4c shows that the nuclear volume increased in those cells expressing the mutant vimentin and the nucleus sphericity was significantly smaller in the transfected cells. These results suggest that, while not being able of generating tension, the vimentin network plays a relevant role protecting the nucleus against forces as was observed in other cell systems [68]. Therefore, we could hypothesize that the intermediate filament network may modulate forces applied to the nucleus in ES cells and consequently, it may also influence gene expression.

### Actin and intermediate filament networks modulate the dynamical organization of OCT4

We have previously used FCS to quantify the dynamics of TFs in the nucleus of living cells (e.g., [69, 70]). The application of this exquisite technique in ES cells revealed that OCT4-chromatin interactions weaken at the onset of differentiation [61] and uncovered how histone acetyltransferase Kat6b modulates OCT4 and NANOG interactions with chromatin [71]. Therefore, we decided to use a similar approach to explore if the dynamical organization of OCT4 responds to alterations of those cytoskeleton networks that modulate the nuclear sphericity.

**Fig. 5.**
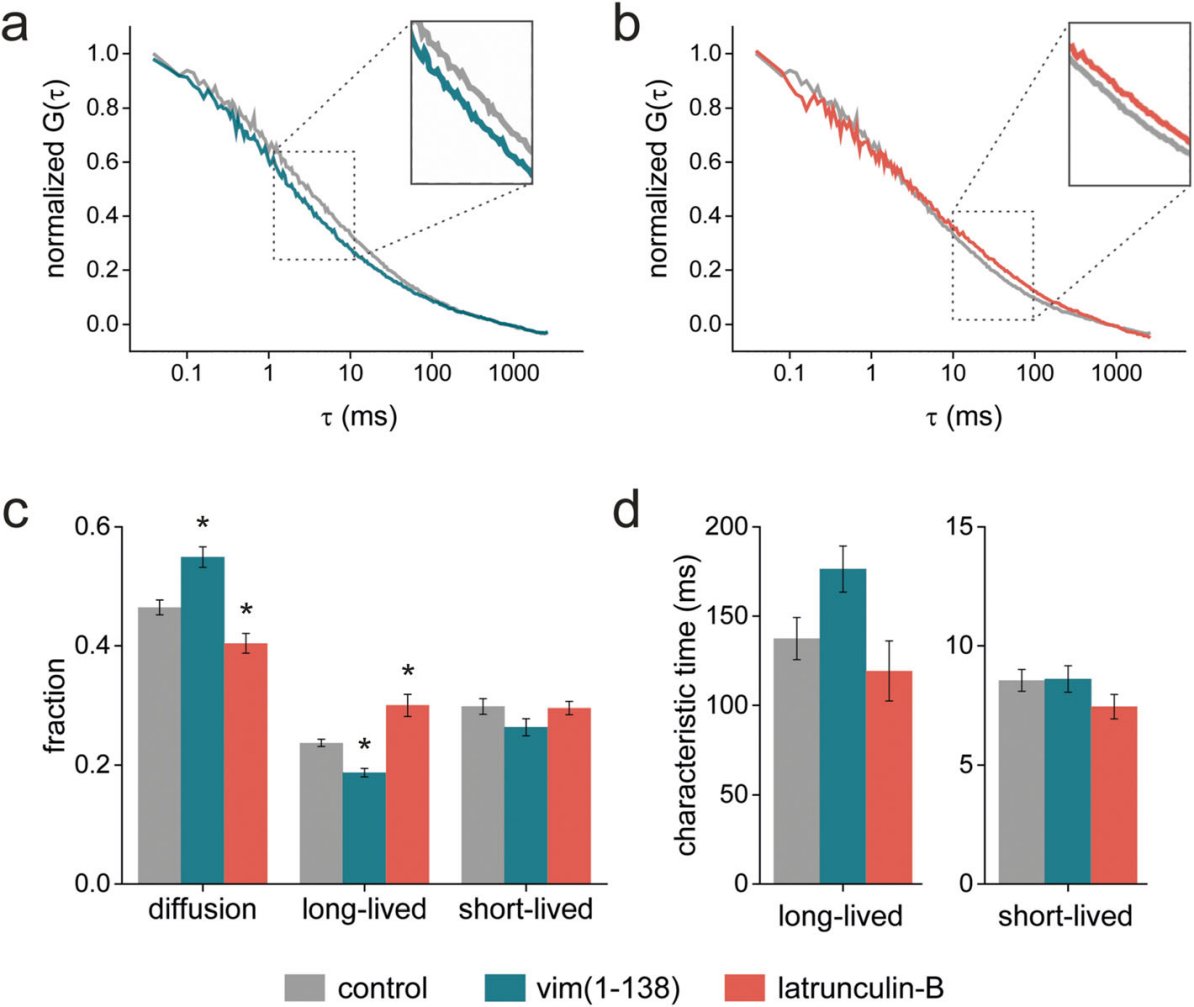
OCT4-chromatin interactions are modulated by the actin and vimentin networks. Single-point FCS measurements were run in YPet-OCT4 ES cells. Mean, normalized ACF obtained at the nucleoplasm of control (gray), vimentin disrupted (**A**, green), and latrunculin-treated (**B**, orange) cells. **C**, **D** The ACF data were fitted with Eq. 1 to obtain the fractions of free (diffusion), long-lived bound and short-lived bound TF molecules (**C**) and the characteristic times of long-lived and short-lived interactions of the TF with chromatin (**D**). The data is presented as mean ± SE for each experimental condition (control: gray bars, *n* = 17; vim(1-138): green bars, *n* = 17; latrunculin-B: orange bars, *n* = 12). Asterisks denote significant differences (*p* < 0.01) with respect to the control condition. Raw data can be found in Additional file 18: Supplementary Table S3

Figure 5 shows mean, normalized autocorrelation functions (ACF) measured for YPet-OCT4 in control, vimentin-disrupted, or actin-depolymerized ES cells treated as described above. In a previous work, we showed that the ACF data of TFs in the cell nucleus follow Eq. 1 that is derived from a model that includes the diffusion of TF molecules in the nucleus and their interactions with chromatin targets in two distinct temporal windows [70]. The fitting of the experimental data with this equation suggests that OCT4 molecules engaged in long- and short-lived interactions with characteristic times similar to those previously reported [61].

The analyses also indicate that disruption of either actin or vimentin networks modified the dynamics of the pluripotency TF (Fig. 5c,d). Interestingly, these treatments affected OCT4 dynamics in opposed ways. Particularly, vimentin disruption promoted the detachment of OCT4 from long-lived chromatin targets with a parallel increase in the proportion of freely diffusing TF molecules. We mentioned before that OCT4-chromatin interactions weaken at the onset of differentiation [61]. In this context, we hypothesize that the intermediate filaments network protects the nucleus from mechanical stimuli thus contributing to the maintenance of OCT4-chromatin interactions and therefore, the pluripotent state. Contrary, actin disruption triggered the attachment of OCT4 molecules to long-lived sites. Therefore, we hypothesize that the actin network communicates mechanical signals to the nucleus affecting the interactions of OCT4 with chromatin targets and probably modifying, in a longer time window, the gene expression profile leading to the exit of the pluripotent state.

On the other hand, the arrest of microtubule polymerization/depolymerization through vinblastine treatment did not affect OCT4 dynamics (Additional file 12: Supplementary Fig. S4) supporting that the microtubule network does not modulate the morphology of the nucleus neither affects OCT4-chromatin interactions.

Taken together, our results suggest that the actin and vimentin filaments networks modulate the landscape of chromatin interactions of the pluripotency TF OCT4 and may ultimately impact on the preservation of the pluripotent state.

## Discussion

Mechanical forces regulate many aspects of cell function [3, 60]. One of the key components involved in the intracellular mechanical communication is the cytoskeleton, an interconnected network of structurally different biopolymers and crosslinking molecules [72]. Forces applied to cells could also be transmitted to the nucleus affecting a variety of nuclear properties and functions including chromatin organization and transcriptional regulation [73–78]. Extensive evidence shows that forces can define cell fate and guide developmental processes [6, 7].

The mechanical interplay between the cytoskeleton and the nucleus has been deeply studied in somatic cells; however, relevant aspects of this communication in ES cells remain elusive. This void in the field is probably due to the fact that most studies describing the cytoskeleton relied on single-plane images of fixed specimens.

In this work, we used non-invasive fluorescence microscopy methods to study the three-dimensional organization of the cytoskeleton in live ES cells and analyzed its influence in the nuclear morphology to explore if certain cytoskeletal components are involved in the transmission of mechanical signals to this organelle. Also, we analyzed if this communication impacts on the dynamical interactions of the pluripotency TF OCT4 with the chromatin.

Our imaging experiments in live ES cells revealed that, contrary to the observations in fixed specimens, microtubules present a complex organization extending throughout the cytoplasm. Time-lapse imaging of EB3-GFP comets highlights the dynamic behavior of the microtubule network and suggests the presence of MTOCs. Microtubules also localize in protrusions that extend to other cells that may be involved in keeping cells together within the colony and/or in cell-cell communication [79]; further, research is necessary to firmly assess their biological function.

We also found that mechanical imbalances in the microtubule network caused by taxol or vinblastine did not significantly affect the nuclear sphericity. This result, combined with the observation through time-lapse imaging of EB3-GFP comets of microtubules growing in contact and even poking the nucleus, suggests that these biopolymers are not involved in modulating its shape; further experiments are required to test their involvement in the rotation and positioning of the nucleus.

Some previous works also described the relevance of microtubules in the nuclear properties but, differently from our study in naïve ES cells, most of these works focus on differentiation processes of multipotent stem cells. For example, differentiation of human adipose-derived stem cells requires a crosstalk between perinuclear microtubules and the LINC complex, and its disruption impairs adipogenesis [77]. Also, microtubules modulate the nucleus shape and affect heterochromatin distribution impacting in human hematopoietic stem cells differentiation. Invaginations generated by microtubules define the distinctive nuclear shape of myeloid progenitors which seem to be relevant to establish the genetic program that identifies this specific cell lineage [76].

A recent report in ES cells [80] reveals microtubule-enriched cytoplasmic bridges that link sister cells for a long time after cell division and shows that the exit from naïve pluripotency requires the abscission of this bridge. We did not observe these bridges in our experiments probably due to the combination of the relatively low proportion of transfected cells and the transient characteristic of these structures [80].

We also analyzed the 3D distribution of actin and found that it concentrates at cell-cell boundaries and cell-substrate contacts as expected from its role in both cell-cell junctions and cell attachment to the substrate [34]. It was recently described that cortical actin in fixed ES cells is organized as an isotropic meshwork [43]. Despite confocal microscopy does not allow resolving this meshwork, our observations provide information on other features of actin organization in living ES cells.

Mouse ES cells did not present the typical actin stress fibers observed in many somatic cell lines nor the actin fence described in human ES cells [36] as expected from their different mechanical properties and interactions with the substrate. Particularly, human pluripotent stem cell colonies are bigger and flatter than those of mouse pluripotent stem cells although in both cases the colonies are composed of tightly compact cells [38–40]. In addition, the composition of the substrate required for feeder-free culture of these cell types is different; mouse pluripotent stem cells can grow on gelatin-coated plates whereas human pluripotent stem cells require more complex coatings such as Matrigel or Geltrex [39, 40] with different mechanical properties [81, 82].

We also observed filopodia-like structures projecting from cells to their closest neighbors; these filopodia resemble those observed in early mouse embryos and required for compaction [42] suggesting that they might be also involved in keeping ES cells tight together in the colony.

Interestingly, ES cells nuclei increased their sphericity upon actin depolymerization, accompanying the loss of cell-cell junctions and cell’s rounding-up. These results suggest that actin is involved in the mechanical coupling between cell and nuclear shapes agreeing with the proposed role of these filaments in strain transmission to the nucleus of ES cells [83], mesenchymal stem cells [84] and endothelial cells [85] among other cell types [2, 86].

Finally, we explored the distribution of vimentin, an intermediate filament protein that remained poorly explored in undifferentiated ES cells since it is expressed at relatively low levels. Relevantly, our analyses of transcriptomic and proteomic data revealed that both vimentin mRNA and protein are detected in these cells.

It is widely accepted that intermediate filaments, the softest component of the cytoskeleton [87], passively contribute to the cell stiffness and protect the nucleus in mechanically stressed situations in somatic cells [68]. These filaments withstand significantly greater mechanical deformation than actin and microtubules [88] with an elastic modulus that increases at large strains [89] and form bundles of increased rigidity in cells [90, 91]. In contrast to microtubules and actin filaments, intermediate filaments do not constitute the tracks of molecular motors and cannot produce and/or respond to external forces by polymerization/depolymerization [87]. Interestingly, recent evidence pointed to more active roles of the vimentin intermediate filament network in the mechanical properties of somatic cells [90–92].

Vimentin has been extensively studied in many other systems due to its role in cell migration associated with both embryogenesis and cancer invasiveness [93, 94]; however, it has been poorly studied in the context of pluripotency. Vast evidence also highlights the involvement of vimentin in multiple differentiation processes since its expression increases during the epithelial-mesenchymal transition [95]. Additionally, it is downregulated during reprogramming to induced pluripotent stem cells generation [13]. Moreover, the absence of vimentin impairs spontaneous in vitro differentiation of ES cells to the endothelial phenotype [12]. A recent report also suggests that vimentin intervenes in the stress response of differentiating cells [96].

We found that vimentin concentrates around the nucleus and forms knots and ring-like structures in mouse ES cells that resemble those observed during processes involving loosely attached cells, i.e., during the initial steps of cell spreading and the detachment step that precedes mitosis [56]. Similar ring-like structures formed by intermediate filaments were proposed to cause nuclear invagination in diverse cell lines [97, 98]. Despite the functional roles of vimentin-structures associated to the nucleus in ES cells remain elusive, we hypothesize that they may interact with other active components of the cytoskeleton as already observed in other cell lines [99–101] modulating the mechanical stimuli applied to the nucleus and consequently protecting it from mechanical stress. In this line, we found that disruption of the vimentin network by expression of a dominant negative vimentin mutant increases the nuclear deformation. Relevantly, this observation also brings in the idea of a role of these intermediate filaments in the transmission of mechanical signals to the nucleus of ES cells.

It is important to emphasize that we run our assays with cells growing onto coverslips, condition widely used in the literature to explore a variety of properties of ES cells. However, many properties of stem cells in 2D and 3D are different [102–104] including the architecture of the cytoskeleton [105] and, even in 2D, the particular characteristics of the substrate influence the behavior of the ES cells [8]. Thus, the observations performed in our experimental conditions cannot be directly extrapolated to other experimental conditions neither to the in vivo context of the embryo.

Finally, we analyzed if those cytoskeleton components that modulate the nuclear shape also triggered changes in other properties of ES cells that may ultimately impact on gene expression and pluripotency maintenance.

Specifically, we studied the dynamics of the pluripotency TF OCT4 through FCS, a technique that provides exquisite information on TFs organization both in single cells and in whole organisms [61, 69–71, 106]. Here, we showed that disruption of either the actin or vimentin networks impact on the dynamical organization of OCT4 whereas the alteration of the microtubule network did not affect the dynamics of this pluripotency TF.

Vimentin disruption induced the detachment of this TF from long-lived chromatin sites with a parallel increase in the relative amount of diffusing OCT4 molecules. In stark contrast, actin depolymerization triggered the binding of OCT4 to long-lived sites with a concomitant reduction of the proportion of TF molecules undergoing diffusion. Altogether, these observations suggest that the cytoskeleton contribute to modulate the nuclear shape and also modify the landscape of OCT4-chromatin interactions.

We have previously reported that OCT4 detaches from chromatin sites at early stages of differentiation preceding its downregulation [61]. In this context, we could hypothesize that the vimentin network protects the nucleus from deformations and contributes with the preservation of the pluripotent state of mouse ES cells. Thus, our results strongly suggest that vimentin may have a pro-stemness role in pluripotent stem cells. In line with this hypothesis, previous reports correlate high vimentin expression with restriction of differentiation during development and cancer [107–111]. Also, the reduction of vimentin levels at early stages of mammalian erythroid cell differentiation seems to be critical for enucleation [112], stressing the relevance of the nucleus-protecting function of vimentin.

On the other hand, our results also highlight the role of actin in modulating the shape of the nucleus that could indirectly guide differentiation. Particularly, we observed that actin depolymerization increased the sphericity of the nucleus and promoted OCT4 binding to chromatin favoring the preservation of the pluripotent state. These results are in line with previous observations showing that weak interactions with the substrate and actin network disruption preserve ES cells pluripotency [83, 113].

In conclusion, our results provide new insights to dissect how the communication between the cytoskeleton and the nucleus of ES cells may impact on pluripotency maintenance and differentiation.

## Conclusions

In this work, we examined the 3D organization of the cytoskeleton of live naïve ES cells and showed how certain cytoskeletal components affect the nuclear shape. We also found that those cytoskeletal components involved in shaping the nucleus (i.e., actin and vimentin intermediate filaments), also modulate the dynamical organization of the pluripotency transcription factor OCT4.

Our data suggest that vimentin protects the nucleus and contributes to maintain OCT4-chromatin interactions thus; it may have a pro-stemness function in ES cells. On the other hand, actin seems to play the opposed role since it contributes to deform the nucleus and triggers the detachment of OCT4 from chromatin sites.

Taken together, our results support a relevant role of the cytoskeleton in communicating signals to the nucleus of ES cells, influencing the landscape of interactions of the transcription factor OCT4 with chromatin and most probably affecting pluripotency and cell fate.

## Methods

### Cell culture

Mouse ES cells were cultured in a medium composed of DMEM (Gibco), 2 mM Glutamax (Gibco), 100 mM MEM nonessential amino acids (Gibco), 0.1 mM 2-mercaptoethanol, 100 U/ml penicillin, and 100 mg/ml streptomycin (Gibco), supplemented with 15% FBS (Gibco), LIF and 2i (1 μM PD0325901 and 3 μM CHIR99021, Tocris). The use of these inhibitors allows culturing ES cells preserving naïve pluripotency [114].

Cells were maintained on 0.1% gelatin coated dishes at 37 °C in a 5 % CO_2_ (v/v) incubator and passaged every 3 days using trypsin (Gibco) and routinely assessed for mycoplasma contamination by genomic DNA extraction and PCR analysis.

The experiments were performed using two cell lines: the mouse ES cell line W4 provided by the Rockefeller University Core Facility and the YPet-OCT4 ES cell line, previously generated in our laboratory from the same W4 cell line [61]. The YPet-OCT4 cell line expresses the TF OCT4 fused to the fluorescent protein YPet in a doxycycline-inducible manner. Cells were incubated with 5 μg/ml doxycycline for 48 h prior to imaging experiments.

### Plasmids and transfection

ES cells were plated for 24 h onto 18-mm round coverslips previously treated with 100 μg/ml PDL (Sigma-Aldrich) and 20 μg/ml Laminin (Invitrogen) which were placed into the wells of a 12-multiwell plate in 800 μl of complete medium. Transient transfection was carried out using Lipofectamine 2000 (Thermo Fisher) and 1.6 μg of plasmid DNA in Opti-MEM medium (Thermo Fisher). The transfection medium was replaced by fresh culture medium 6 h after transfection and microscopy observations were performed 48 h after transfection.

The plasmids were GFP-tagged full-length vimentin and the dominant-negative construct containing the head and alpha-helical domain 1A of vimentin [mCherry-vim(1-138)] generated from the GFP-vim(1-138) plasmid [67] that was provided by Dr. Vladimir I Gelfand (Northwestern University, Chicago, IL); EMTB-3xGFP [17], which codifies the microtubule-binding domain of ensconsin fused to a tandem of 3 copies of GFP (Addgene # 26741) and EB3-GFP which binds to the plus-end of growing microtubules [115] were gifts from Dr. Arpita Upadhyaya (University of Maryland, College Park, MD); PGK-H2B-mCherry was a gift from Mark Mercola (Addgene plasmid # 21217; http://n2t.net/addgene:21217 ; RRID:Addgene_21217) [116] and pEGFP-actin [117] kindly provided by Dr. Nicolás Plachta (Institute of Molecular and Cell Biology, ASTAR, Singapore).

### Sample preparation for imaging

For microscopy measurements, ES cells were plated onto 18-mm round coverslips coated with PDL and laminin as described above. Before observation, the coverslips were mounted in a custom-made chamber specially designed for the microscope.

Cells were incubated with 10 μM latrunculin-B (Sigma-Aldrich) at 37 °C for 15 min or 10 μM nocodazole at 0 °C for 30 min to promote actin and microtubule depolymerization, respectively. To perturb microtubule dynamics, cells were incubated at 37 °C with 30 nM paclitaxel for 4 h or 30 nM vinblastine sulfate (Sigma-Aldrich) for 10 min.

### Confocal microscopy

Confocal images were acquired in FV1000 Olympus confocal microscopes (Olympus Inc., Japan). GFP, EGFP, YPet, and mCherry fusion proteins were observed using a multi-line Ar laser tuned at 488 nm and a solid diode laser of 543 nm as excitation sources, respectively. The average power at the sample was ~ 1 μW. The laser light was reflected by a dichroic mirror (DM 405/488/543/635) and focused through an Olympus UPlanSApo 60X oil immersion objective (NA = 1.35) onto the sample. Fluorescence was collected by the same objective and split into two channels set to collect photons in the range 500–525 nm (GFP, EGFP and YPet) and 650–750 nm (mCherry). Fluorescence was detected with photomultipliers set in the photon-counting detection mode.

### Tracking of EB3 comets

We used the Trackmate plugin [118] of Fiji ImageJ (NIH, USA) to track EB3 comets; the images stacks were preprocessed using the despeckle filter of the same program. These data were exported to Icy [119] to obtain the flow map.

### 3D image analyses

Z–stack images were preprocessed using median and ROF filters in ImageJ (NIH, USA) and analyzed using the automatic surface rendering mode of the software Imaris (Bitplane) that was also used to calculate the morphological descriptors sphericity and volume of cell nuclei. Examples of nuclei segmentation can be found in Additional file 13: Supplementary Fig S5.

### Fluorescence correlation spectroscopy (FCS)

Single-point FCS measurements were performed in the Olympus FV1000 confocal microscope set in the photon-counting mode. The laser was focused at a position in a cell nucleus selected by the user and the intensity was collected at 50 MHz during ~ 3 min. Single experiment was performed in each cell to minimize its photodamage.

ACF data were calculated using SimFCS program (LFD, Irvine, CA, USA) and were fitted with Eq. 1 that considers the diffusion of the TFs and their binding to two populations of fixed sites [70]:

1 $$ \mathrm{G}\left(\uptau \right)=\frac{1}{2^{3/2}N}\left[{f}_{\mathrm{D}}{\left(1+\frac{\tau }{\tau_{\mathrm{D}}}\right)}^{-1}{\left(1+\frac{\tau }{\omega^2{\tau}_{\mathrm{D}}}\right)}^{-1/2}+{f}_{\mathrm{short}}{e}^{\raisebox{1ex}{$-\tau $}\!\left/ \!\raisebox{-1ex}{${\tau}_{\mathrm{short}}$}\right.}+{f}_{\mathrm{long}}{e}^{\raisebox{1ex}{$-\tau $}\!\left/ \!\raisebox{-1ex}{${\tau}_{\mathrm{long}}$}\right.}\right] $$

where *N* is the mean number of fluorescent molecules in the confocal volume, τ_D_ is the characteristic diffusion time, ω is the ratio between axial and radial waists of the observation volume, and *f*_D_ is the freely diffusing population fraction. *f*_short_ and *f*_long_ are the population fractions bound to short-lived and long-lived targets, and τ_short_ and τ_long_ are their residence times, respectively. The reciprocal of the residence time corresponds to the dissociation constant k_off_.

### Bioinformatics analysis

Vimentin gene expression analysis was performed on transcriptomic and proteomic data-mining platform, Stemformatics web tool (https://www.stemformatics.org, [120]), using publicly available datasets (Additional file 7: Supplementary Table S1, [48–55]) stored in Gene Expression Omnibus (GEO, ncbi), Sequence Read Archive (ncbi), GnomEx (Utah) and ProteomeXchange. Data normalization, transformation and annotation methods are available at Stemformatics documentation (https://www.stemformatics.org/Stemformatics_data_methods.pdf).

### Statistical analysis

All the results shown in this work were obtained from experiments replicated at least 3 times. Nuclear volume and sphericity were expressed as median ± SE. To compare the median values (med) of different data sets, we used a hypothesis test computing the *p*-values as follows [121]:

2 $$ p-\mathrm{value}=2\left[1-F\left(\frac{\left|{\mathrm{med}}_{\left(\mathrm{g}1\right)}-{\mathrm{med}}_{\left(\mathrm{g}2\right)}\right|}{\sqrt{\upsigma_{\left(\mathrm{g}1\right)}^2+{\upsigma}_{\left(\mathrm{g}2\right)}^2}}\right)\right] $$

where *F* is the standard normal distribution and σ2 (g1) and σ2 (g2) represent the variance of each data group. Differences were regarded as significant at *p* < 0.05.

The parameters’ standard errors (SE) and variance were computed by a bootstrap procedure [122].

Experimental results obtained for OCT4 dynamics were expressed as mean ± SEM. Statistical significance between groups was analyzed using linear mixed models (LMM) followed by comparisons between means using the Dunett test, when required. Differences were regarded as significant at *p* ≤ 0.01. Statistical data analysis was performed using the R software.

## Supplementary Information

**Additional file 1.** Supplementary Video S1. 3D organization of the microtubule network. Representative 3D confocal images of ES cells transfected with EMTB-3xGFP (green) and H2B-mCherry (red) (Scale bar: 5 μm). Related to Fig. 1a, top panel.

**Additional file 2.** Supplementary Video S2. 3D organization of the microtubule network. Representative 3D confocal images of ES cells transfected with EMTB-3xGFP (green) and H2B-mCherry (red) (Scale bar: 5 μm). Related to Fig. 1a, bottom panel.

**Additional file 3.** Supplementary Fig. S1. ES cells exhibit long membrane protrusions. (left) Representative confocal image of ES cells expressing YPet-OCT4 (green) and mem-mCherry (red) collected at a single plane of the z-stack (Scale bar: 10 μm). (middle) 3D reconstruction of the images showing a protrusion extending from one cell to a neighboring cell. (right) Zoom-in image of the same cell, the protrusion is indicated with an asterisk; the red image was digitally saturated to facilitate the visualization of the protrusion.

**Additional file 4.** Supplementary Video S3. EB3-GFP comets point to every direction. ES cells transfected with EB3-GFP and H2B-mCherry were imaged at 0.6 frames/s (100 frames) to observe the dynamics of EB3-GFP comets. Related to Fig. 1d.

**Additional file 5.** Supplementary Video S4. EB3-GFP comets irradiate from a specific site in the cytoplasm. ES cells transfected with EB3-GFP and H2B-mCherry were imaged at 0.6 frames/s (100 frames) to capture the dynamical behavior of EB3-GFP comets. Related to Fig. 1d.

**Additional file 6.** Supplementary Video S5. EB3-GFP comets in close contact with the cell nucleus. ES cells transfected with EB3-GFP and H2B-mCherry were imaged at 0.6 frames/s (100 frames). Related to Fig. 1c.

**Additional file 7.** Supplementary Table S1. Meta-analysis of microarray, RNA-seq and proteomic datasets analyzed in this work.

**Additional file 8 **Supplementary Fig. S2. Omics data analysis of vimentin expression in mouse embryo and different cell types. Data analysis of vimentin expression from microarray, RNA-seq and proteomics (as indicated in each panel) performed in Stemformatics data-mining platform. Bars represent mean ± SEM when corresponding. Full meta-data of analyzed datasets is available at Additional file 7: Supplementary Table S1. **A** The left panel shows the comparison between embryonic stem (ES) and epiblast-derived stem (EpiS) cells from different development stages of mouse embryo: Cavity (CAV, E5.5 – E6.0); Pre-primitive streak (PS; E6.0 – E6.5); Late Mid Streak (LMS; E6.75 – E 7.25); Late Streak (LS; E7.25 – E7.5); Early Bud (EB; E7.75) and Late Bud (LB; E8.0). The right panel shows data from different tissues during advanced embryo development. **B** Data from different stem cell types: ES cells, mesenchymal stem (MS) cells and multipotent adult progenitor (MAP) cells. **C** Data from ES cells differentiation experiments. The left panel shows vimentin mRNA levels from ES cells and neural progenitor (NP) cells. The right panel shows data from ES cells and ES cells-derived mesoderm cells, cardiac progenitors and cardiomyocytes. **D** Data obtained from ES cells during their differentiation to epiblast-like stem (EpiLS) and primordial germ cell-like (PGCL) cells. **E** Data from ES cells, mouse embryonic fibroblasts (MEF) and induced pluripotent stem (iPS) cells. **F** RNA-seq (left panel) and proteomic (right panel) data of ES cells, and MEF during their reprogramming to iPS cells.

**Additional file 9.** Supplementary Video S6. 3D organization of the vimentin network. Representative 3D confocal images of ES cells transfected with GFP-vimentin (green) and H2B-mCherry (red). (Scale bar: 5 μm). Related to Fig. 3b, top panel.

**Additional file 10.** Supplementary Video S7.3D organization of the vimentin network. Representative 3D confocal images of ES cells transfected with GFP-vimentin (green) and H2B-mCherry (red). (Scale bar: 5 μm). Related to Fig. 3b, bottom panel.

**Additional file 11.** Supplementary Fig. S3. Morphology of the ES cells colonies after different treatments that disturb the cytoskeleton. Representative transmission (top panels) and fluorescence (bottom panels) images of colonies of YPet-OCT4 ES cells and W4 ES cells, (green: nuclei). YPet-OCT4 cells were registered in control condition and treated with latrunculin-B, nocodazole, taxol or vinblastine, whereas W4 ES cells were transfected with GFP-vim(1–138). The last image was saturated digitally to facilitate the visualization of out-of-focus cells (Scale bars: 10 μm). The bottom panel shows a 3D section of the nocodazole-treated colony to exhibit its detachment from the substrate.

**Additional file 12 **Supplementary Fig. S4. OCT4-chromatin interactions are not affected by the microtubules network. Single-point FCS measurements were run in YPet-OCT4 ES cells. **A** Mean, normalized ACF obtained at the nucleoplasm of control (gray) and vinblastine-treated (violet) cells. **B,C** The ACF data were fitted with Eq. 1 to obtain the fractions of free (diffusion), long-lived bound and short-lived bound TF (**B**) and the characteristic times of long-lived and short-lived interactions of the TF with chromatin (**C**). These experiments were run using a higher laser power that could explain the slightly different characteristic times from those showed in Fig. 5. The data is presented as mean ± SE for each experimental condition (control: gray bar, *n*=16, vinblastine: violet bar, *n*=16).

**Additional file 13.** Supplementary Fig. S5. Comparison of raw z-stack images before and after nuclei segmentation.

**Additional file 14.** Supplementary Fig. S6. Representative 3D images of ES cells expressing EMTB-3xGFP (green) and H2B-mCherry (red). Related to Fig. 1a.

**Additional file 15.** Supplementary Video S8. Representative time-lapse images of EB3-GFP comets. ES cells transfected with EB3-GFP and H2B-mCherry were imaged at 0.6 frames/s (100 frames) to observe the dynamics of EB3-GFP comets. Related to Fig. 1c and d.

**Additional file 16.** Supplementary Fig. S7. Quantification of EGFP-actin fluorescence intensity along membrane blebs. Related to Fig. 2c and d.

**Additional file 17.** Supplementary Table S2. Related to Fig. 4. Raw data.

**Additional file 18.** Supplementary Table S3. Related to Fig. 5. Raw data.

## Data Availability

All data generated or analyzed during this study are included in this published article, its supplementary information files, and publicly available repositories. 3D images of ES cells are available in Figshare: EB3-GFP and H2B-mCherry: https://figshare.com/s/4df2870709faf1618ae8 [123]. EMTB-3GFP and H2B-mCherry: https://figshare.com/s/828fcb4d80ddead23574 [124]. Actin-GFP and H2B-mCherry: https://figshare.com/s/2dfcf066f4a10839ad47 [125]. Vim-GFP and H2B-mCherry: https://figshare.com/s/74edbb5a93ae74caa9e8 [126].

## Abbreviations

ACF: Autocorrelation functions
EB3: Microtubule end-binding protein
EGFP: Enhanced green fluorescent protein
EMTB: Ensconsin microtubule-binding domain
EpiLCs: Epiblast-like cells
ES cells: Embryonic stem cells
FCS: Fluorescence correlation spectroscopy
GFP: Green fluorescent protein
iPS cells: Induced pluripotent stem cells
lat: Latrunculin-B
LINC: Linker of nucleoskeleton and cytoskeleton
mCherry: Monomeric red fluorescent protein
MEFs: Mouse embryonic fibroblasts
MTOCs: Microtubule-organizing centers
RNA-seq: RNA sequencing
tax: Taxol
TF: Transcription factor
vim(1-138): Dominant negative vimentin mutant
vbl: Vinblastine
YPet: Yellow fluorescent protein
