# Supplementary figures and images for: Nucleus-cytoskeleton communication impacts on OCT4-chromatin interactions in embryonic stem cells
Source: BMC Biol. 2022 Jan 7;20:6. doi: 10.1186/s12915-021-01207-w (PMC8742348; doi:10.1186/s12915-021-01207-w)

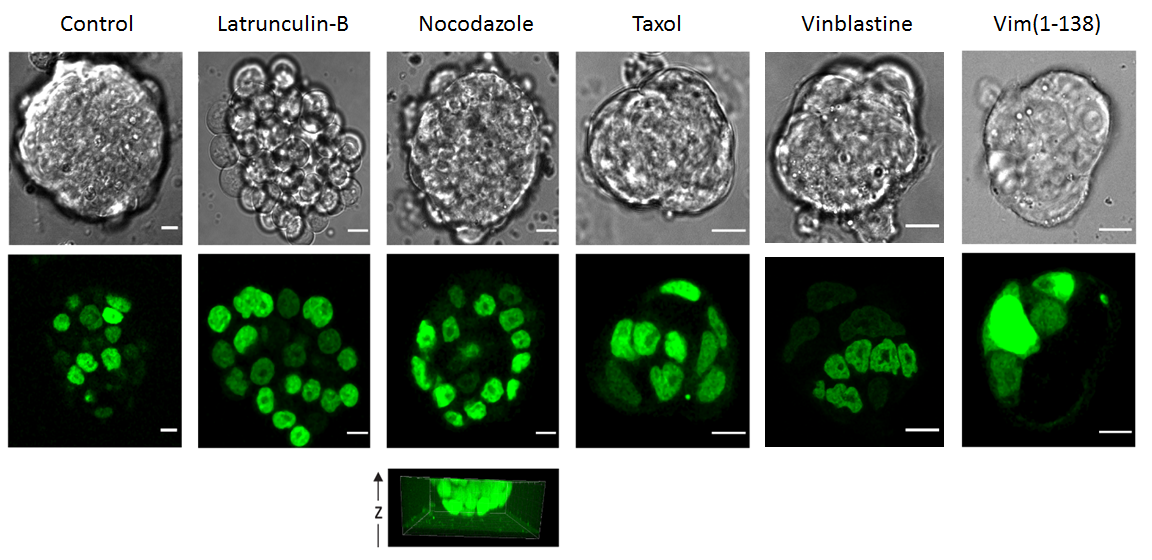

Supplement: Supplementary file 3 — Additional file 3. Supplementary Fig. S1. ES cells exhibit long membrane protrusions. (left) Representative confocal image of ES cells expressing YPet-OCT4 (green) and mem-mCherry (red) collected at a single plane of the z-stack (Scale bar: 10 μm). (middle) 3D reconstruction of the images showing a protrusion extending from one cell to a neighboring cell. (right) Zoom-in image of the same cell, the protrusion is indicated with an asterisk; the red image was digitally saturated to facilitate the visualization of the protrusion. [file 12915_2021_1207_MOESM3_ESM.tif]

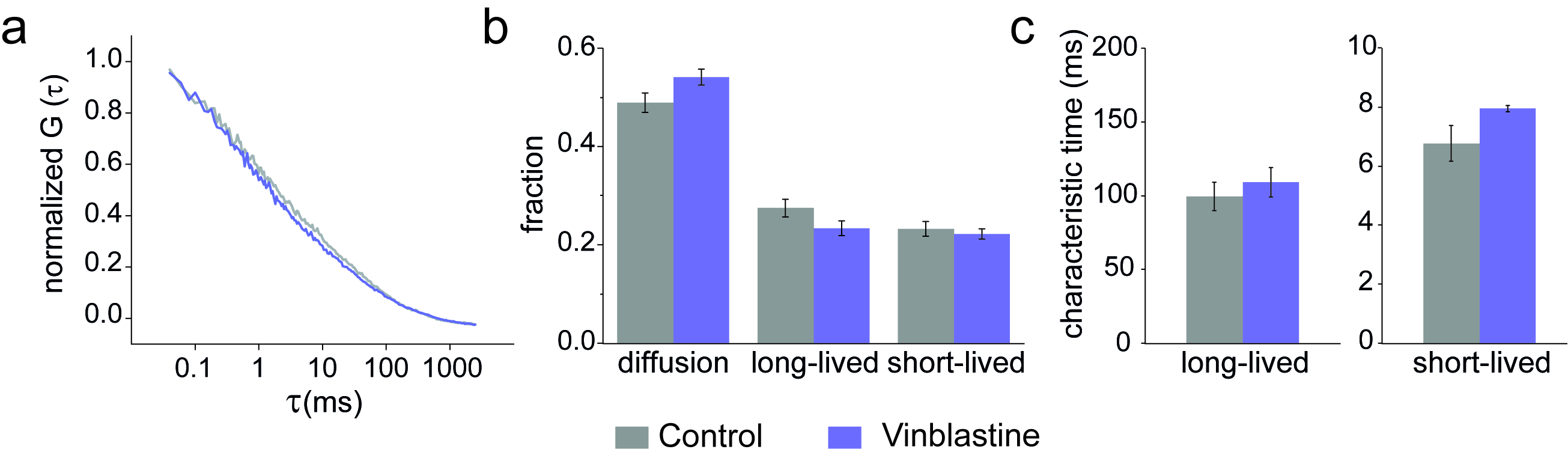

Supplement: Supplementary file 4 — Additional file 4. Supplementary Video S3. EB3-GFP comets point to every direction. ES cells transfected with EB3-GFP and H2B-mCherry were imaged at 0.6 frames/s (100 frames) to observe the dynamics of EB3-GFP comets. Related to Fig. 1d. [file 12915_2021_1207_MOESM4_ESM.tif]

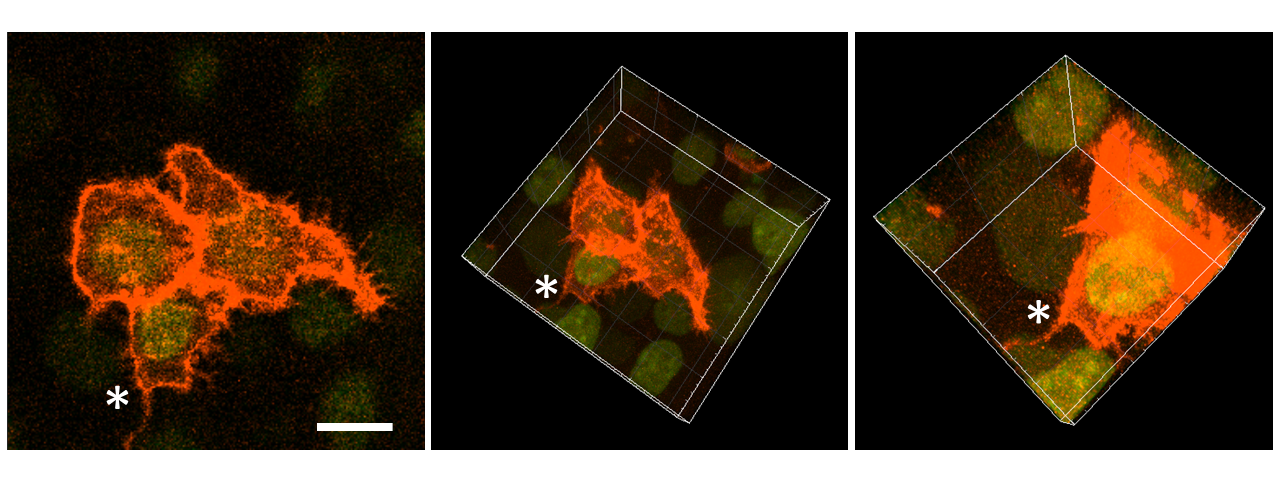

Supplement: Supplementary file 12 — Additional file 12 Supplementary Fig. S4. OCT4-chromatin interactions are not affected by the microtubules network. Single-point FCS measurements were run in YPet-OCT4 ES cells. A Mean, normalized ACF obtained at the nucleoplasm of control (gray) and vinblastine-treated (violet) cells. B,C The ACF data were fitted with Eq. 1 to obtain the fractions of free (diffusion), long-lived bound and short-lived bound TF (B) and the characteristic times of long-lived and short-lived interactions of the TF with chromatin (C). These experiments were run using a higher laser power that could explain the slightly different characteristic times from those showed in Fig. 5. The data is presented as mean ± SE for each experimental condition (control: gray bar, n=16, vinblastine: violet bar, n=16). [file 12915_2021_1207_MOESM12_ESM.tif]

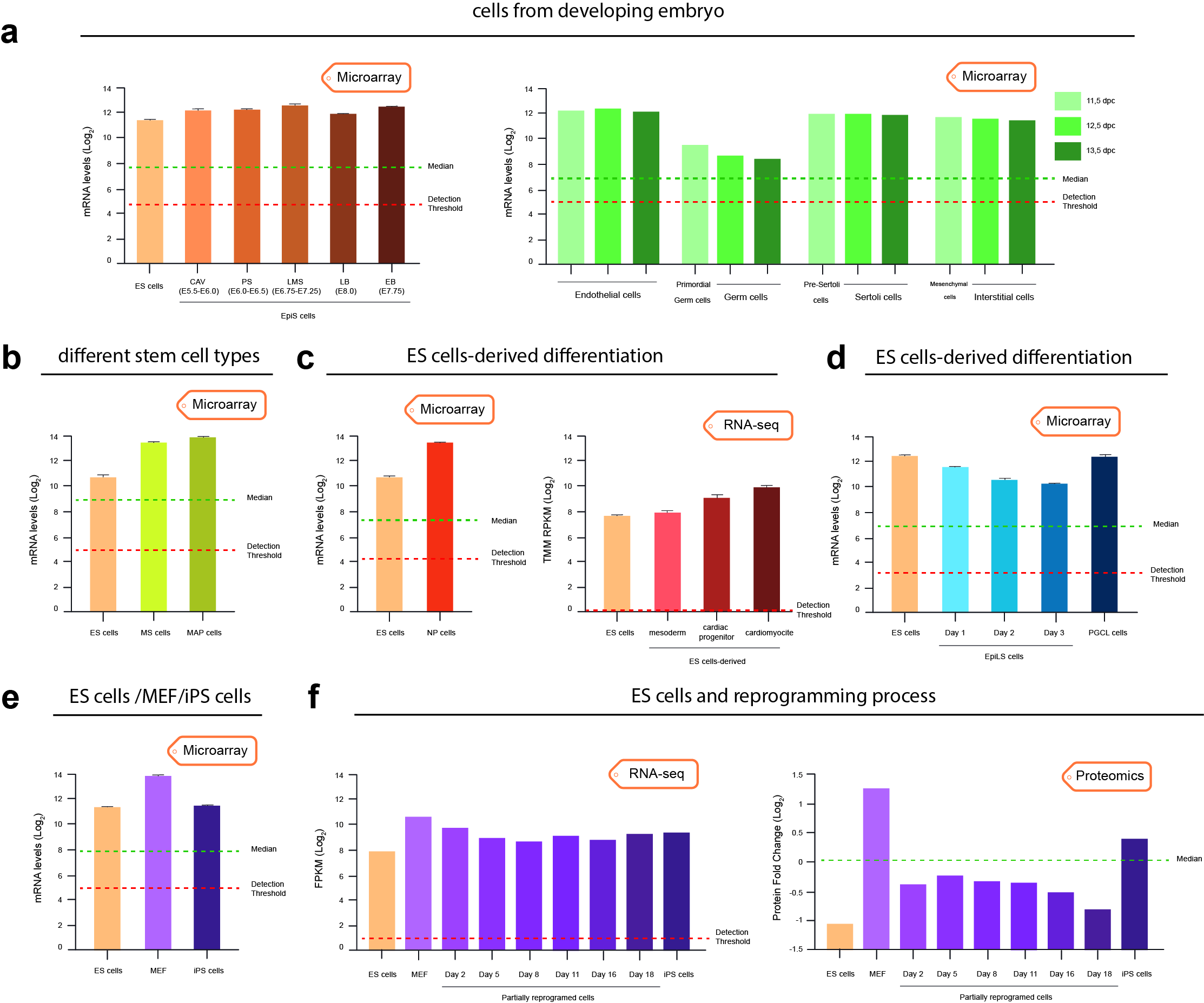

Supplement: Supplementary file 17 — Additional file 17. Supplementary Table S2. Related to Fig. 4. Raw data. [file 12915_2021_1207_MOESM17_ESM.tif]
