# Supplementary material for: Nucleus-cytoskeleton communication impacts on OCT4-chromatin interactions in embryonic stem cells
Source: BMC Biol. 2022 Jan 7;20:6. doi: 10.1186/s12915-021-01207-w (PMC8742348; doi:10.1186/s12915-021-01207-w)
Supplement: Supplementary file 5 — Additional file 5. Supplementary Video S4. EB3-GFP comets irradiate from a specific site in the cytoplasm. ES cells transfected with EB3-GFP and H2B-mCherry were imaged at 0.6 frames/s (100 frames) to capture the dynamical behavior of EB3-GFP comets. Related to Fig. 1d. [file 12915_2021_1207_MOESM5_ESM.pptx]

## Slide 1
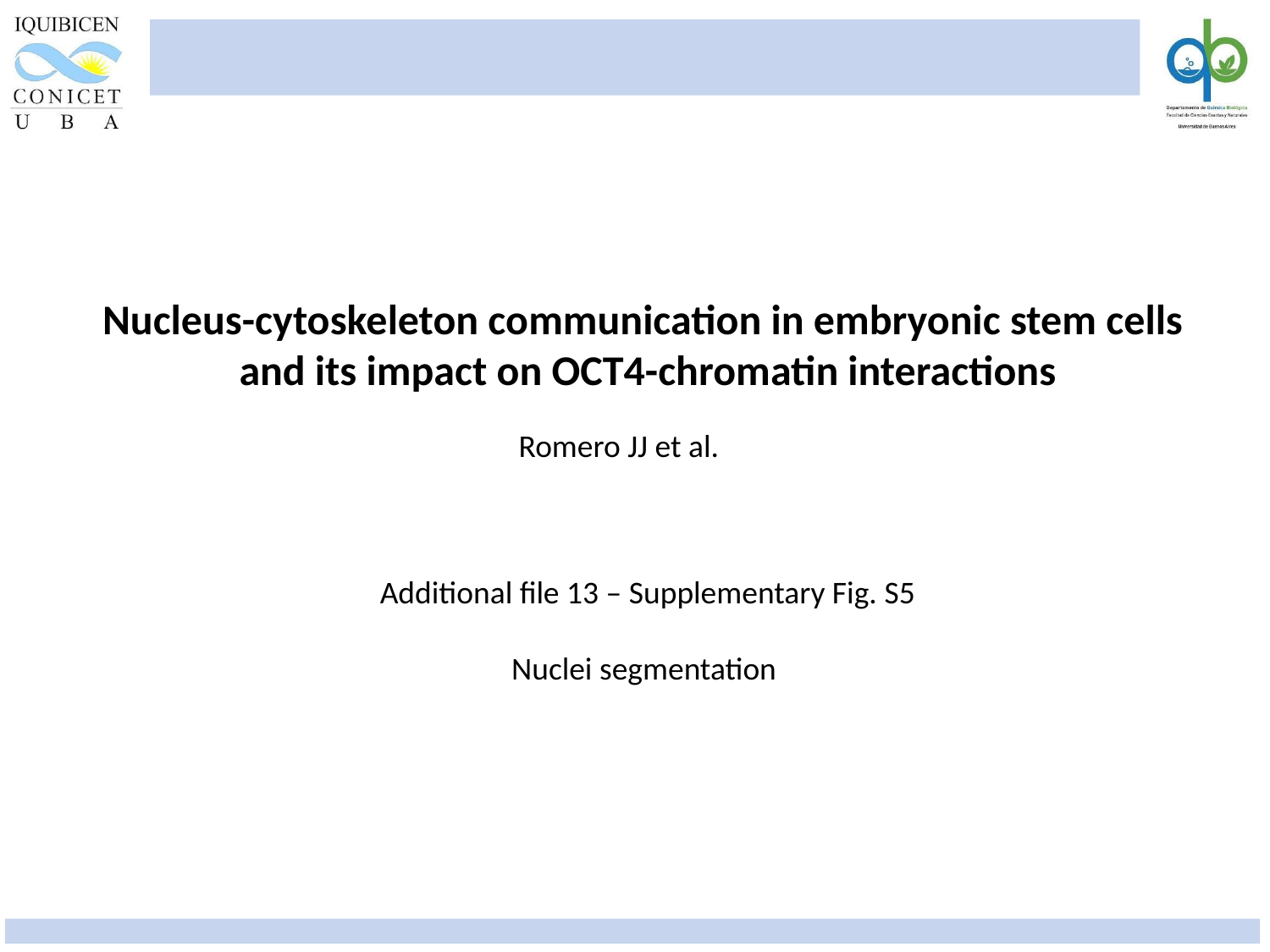

Nucleus-cytoskeleton communication in embryonic stem cells
and its impact on OCT4-chromatin interactions
Romero JJ et al.
Additional file 13 – Supplementary Fig. S5
Nuclei segmentation

## Slide 2
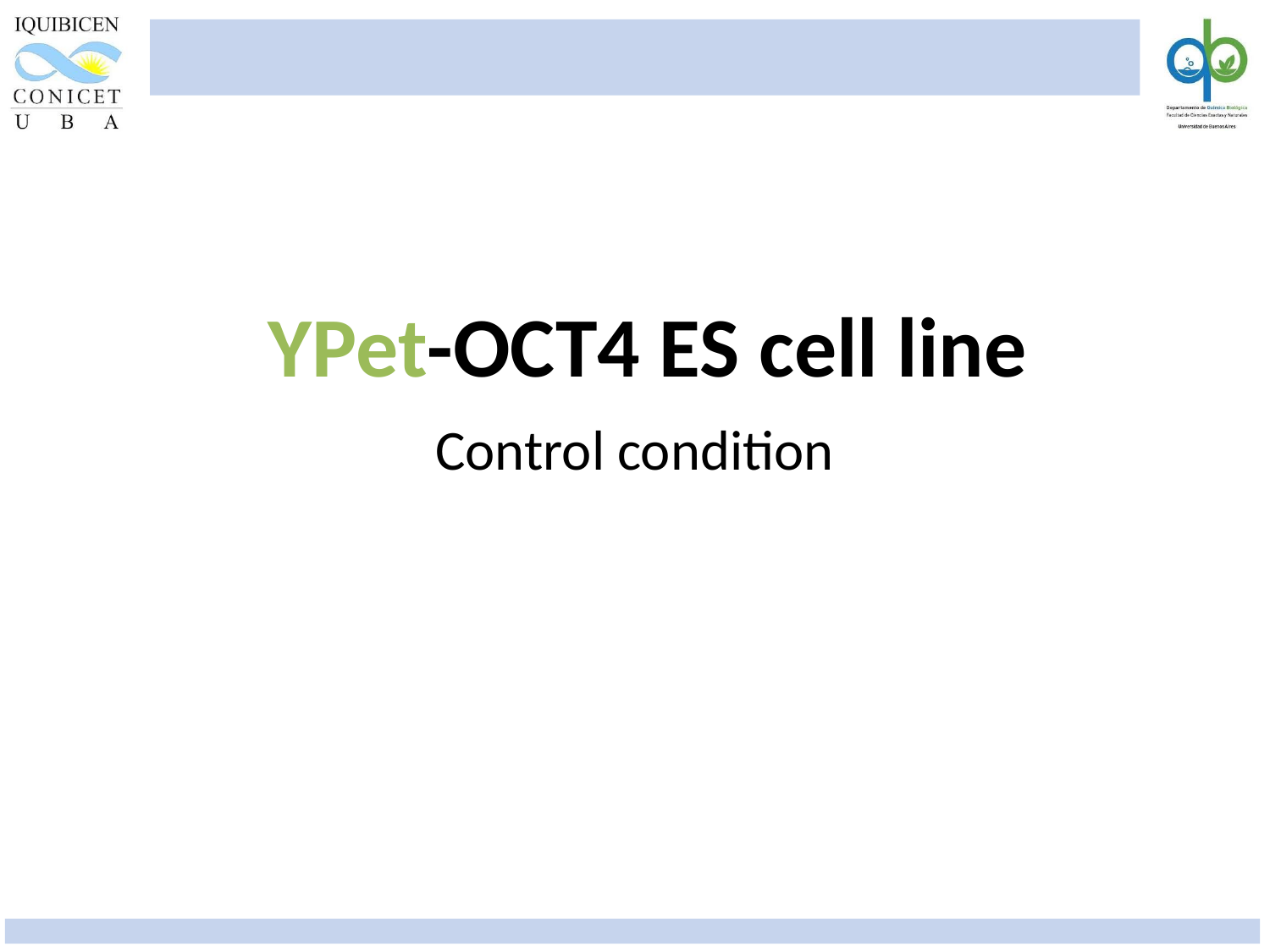

YPet-OCT4 ES cell line
Control condition

## Slide 3
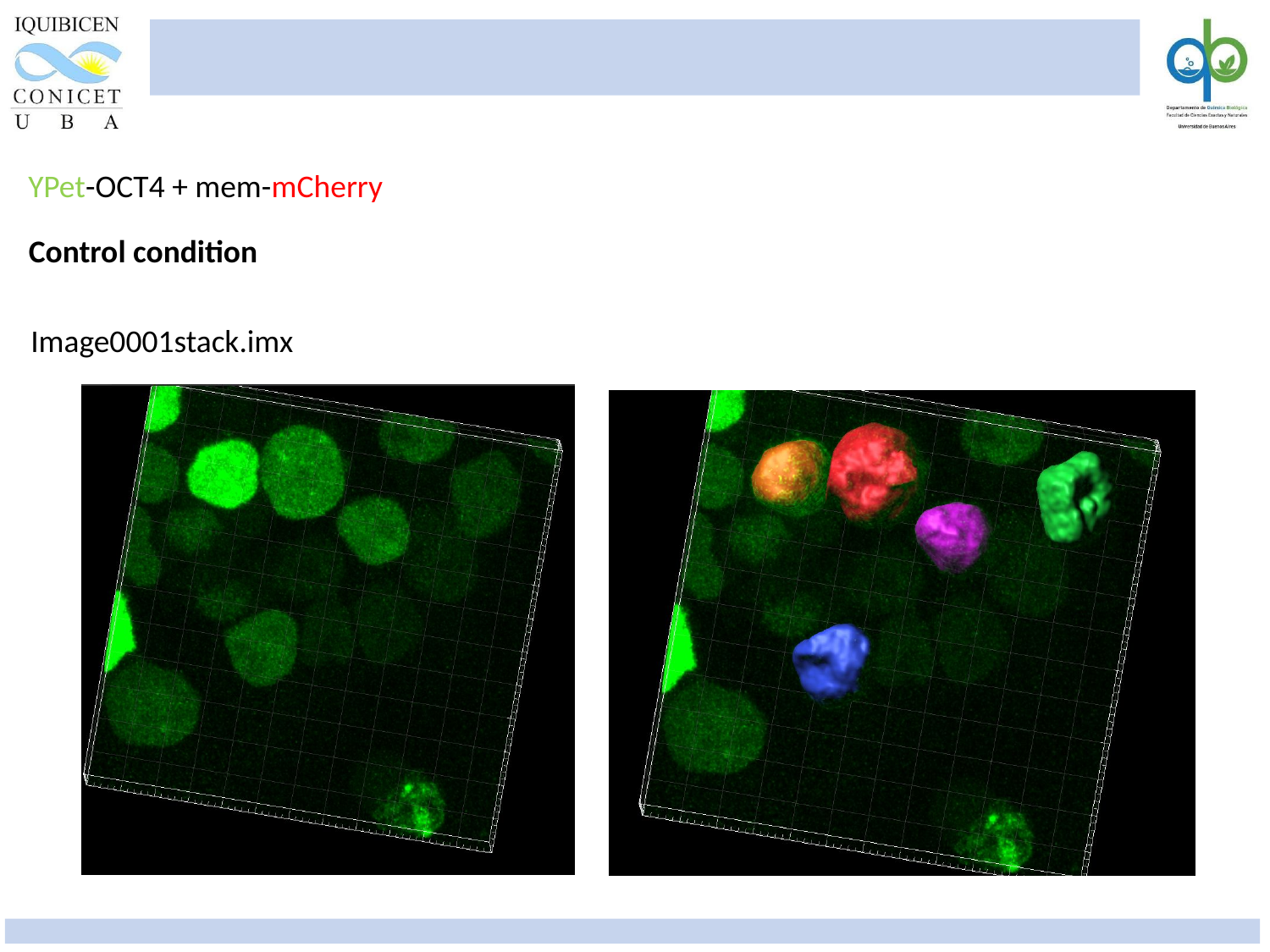

YPet-OCT4 + mem-mCherry
Control condition
Image0001stack.imx

## Slide 4
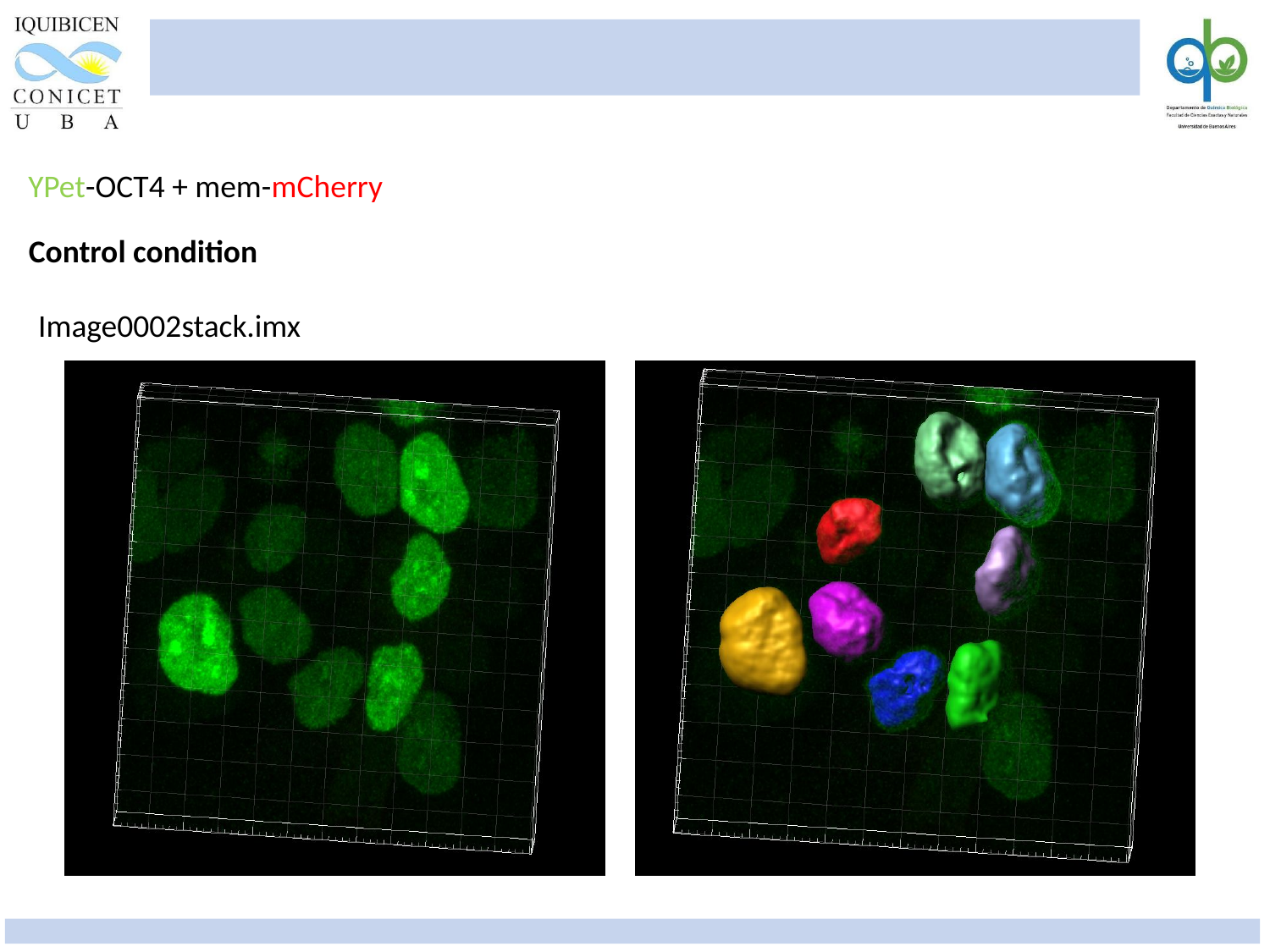

YPet-OCT4 + mem-mCherry
Control condition
Image0002stack.imx

## Slide 5
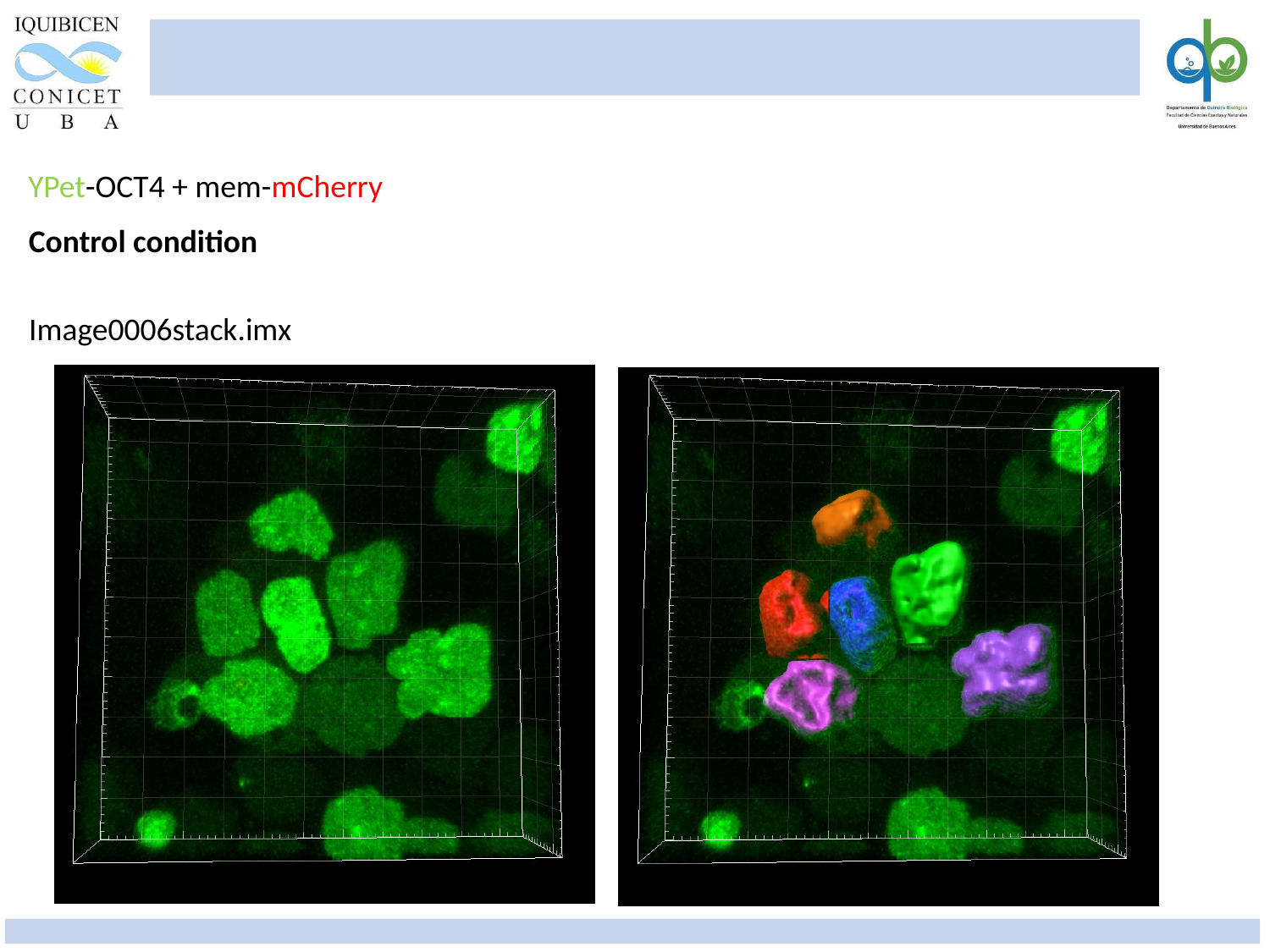

YPet-OCT4 + mem-mCherry
Control condition
Image0006stack.imx

## Slide 6
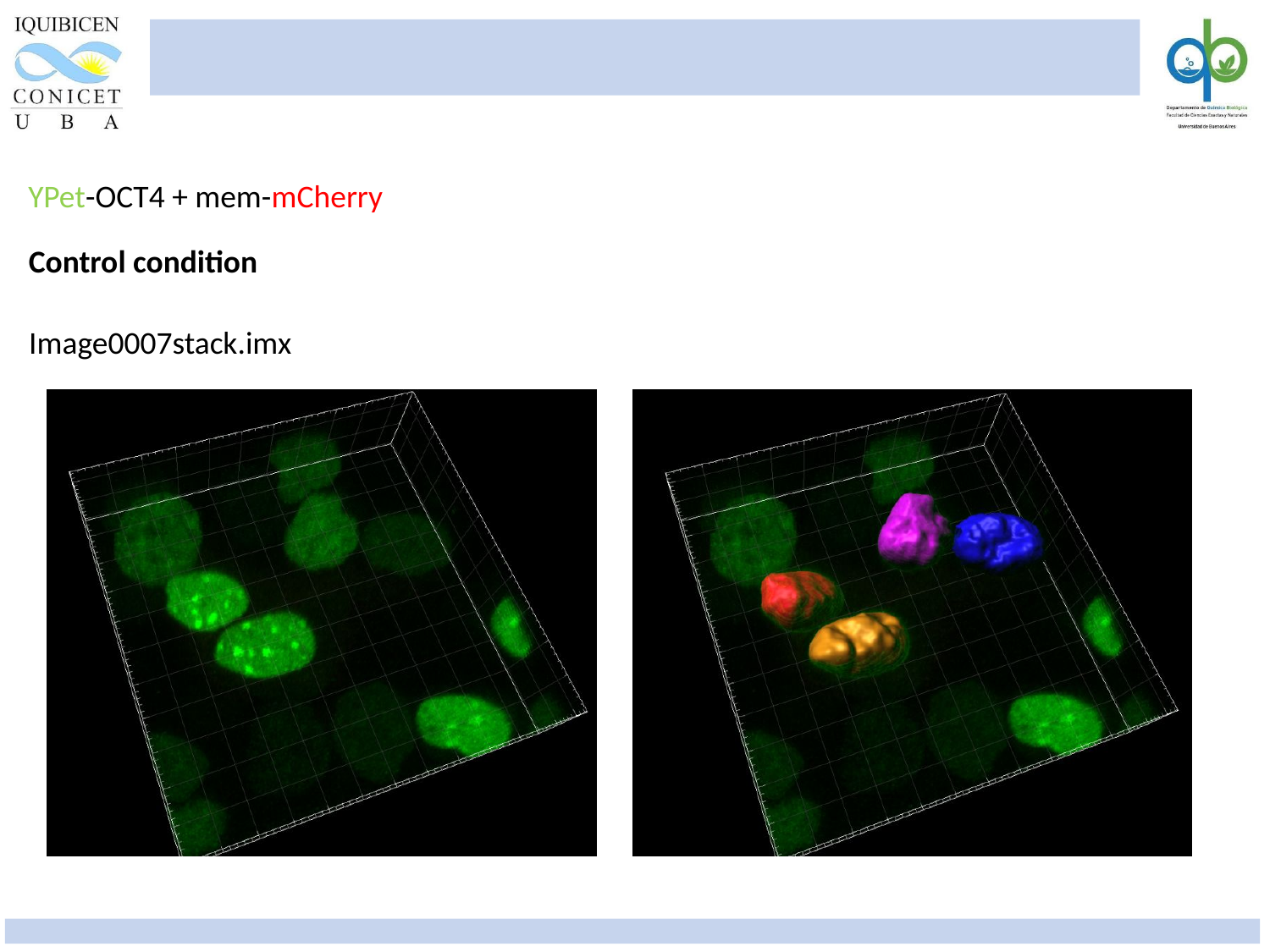

YPet-OCT4 + mem-mCherry
Control condition
Image0007stack.imx

## Slide 7
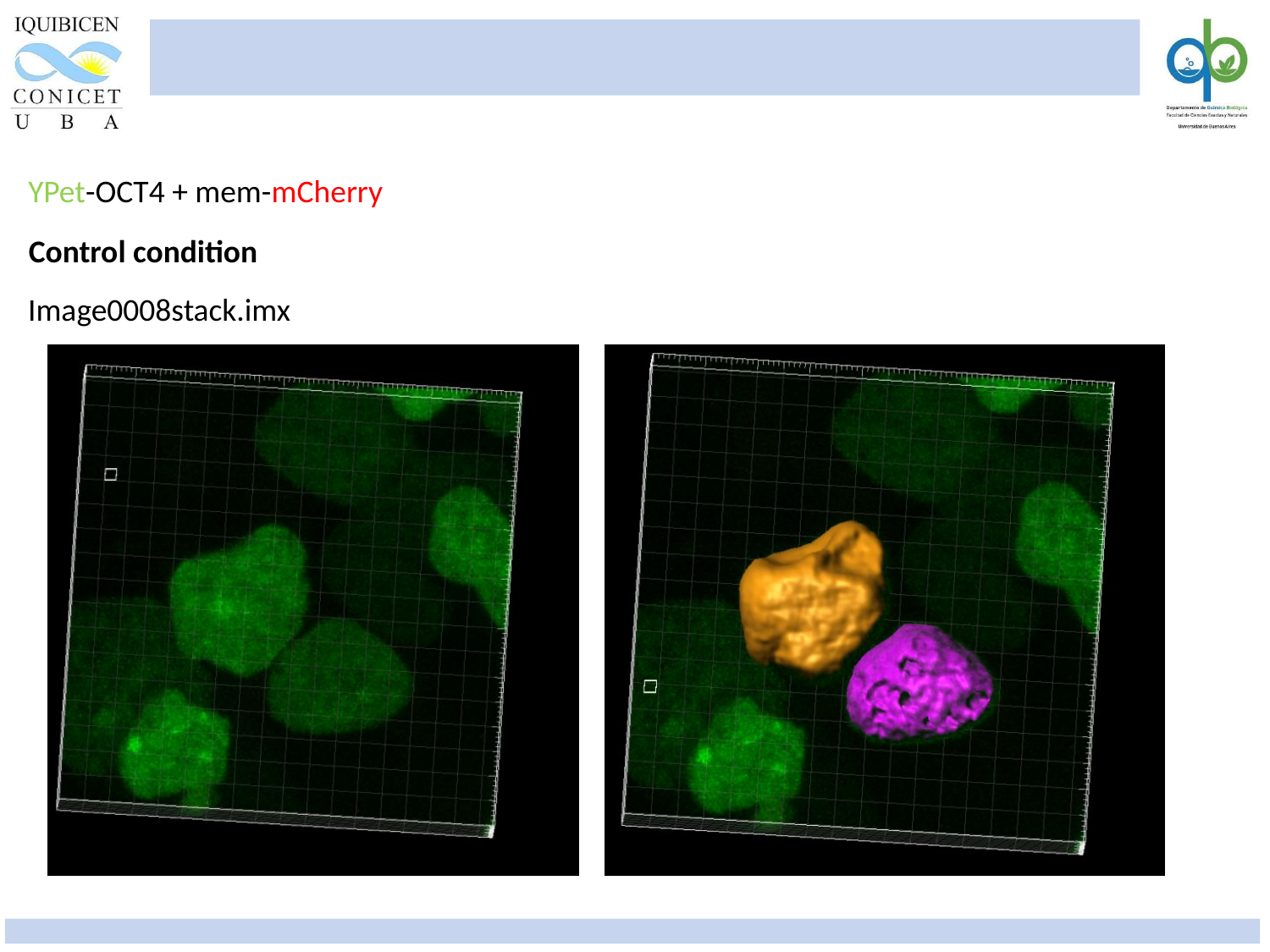

YPet-OCT4 + mem-mCherry
Control condition
Image0008stack.imx

## Slide 8
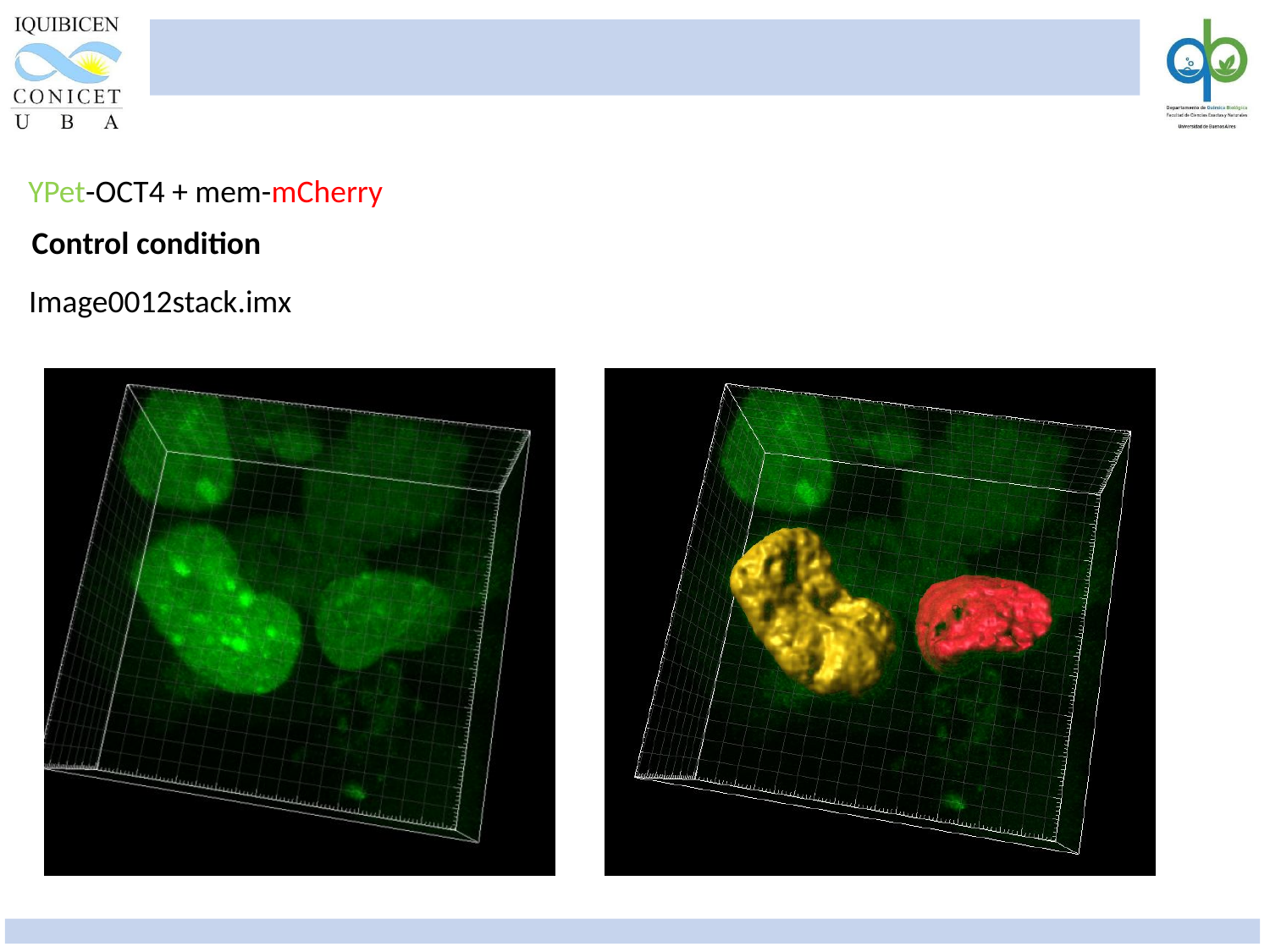

YPet-OCT4 + mem-mCherry
Control condition
Image0012stack.imx

## Slide 9
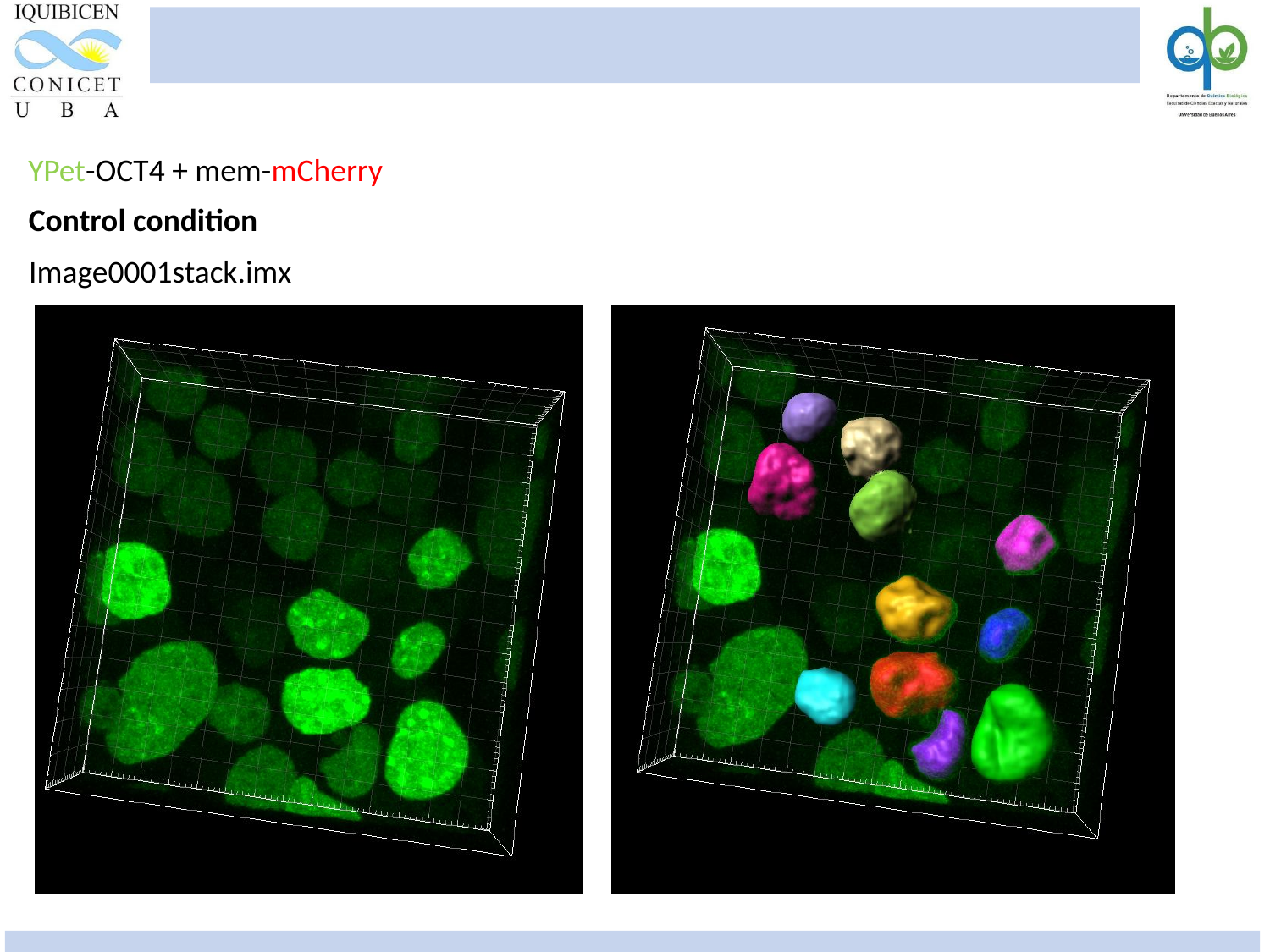

YPet-OCT4 + mem-mCherry
Control condition
Image0001stack.imx

## Slide 10
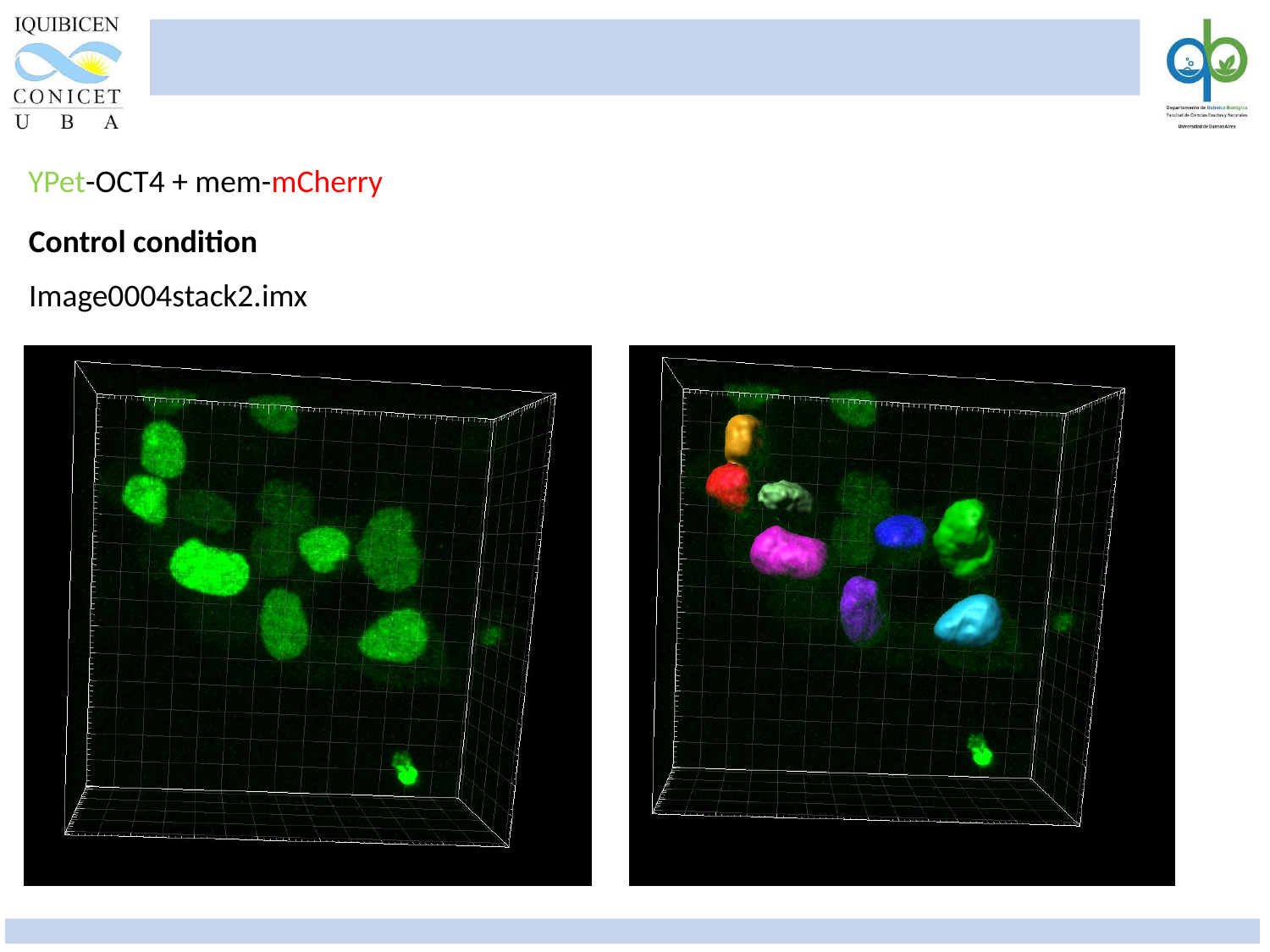

YPet-OCT4 + mem-mCherry
Control condition
Image0004stack2.imx

## Slide 11
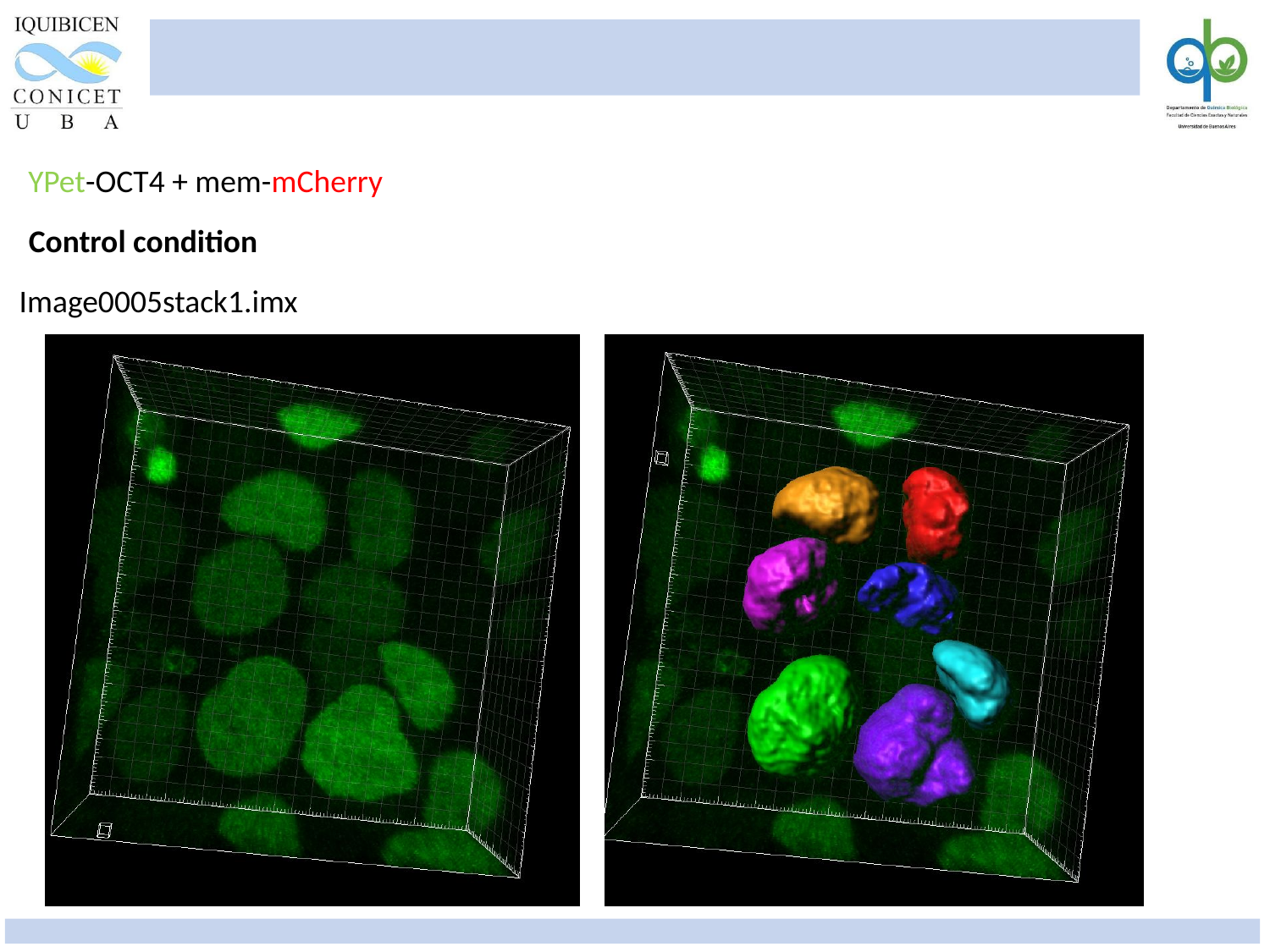

YPet-OCT4 + mem-mCherry
Control condition
Image0005stack1.imx

## Slide 12
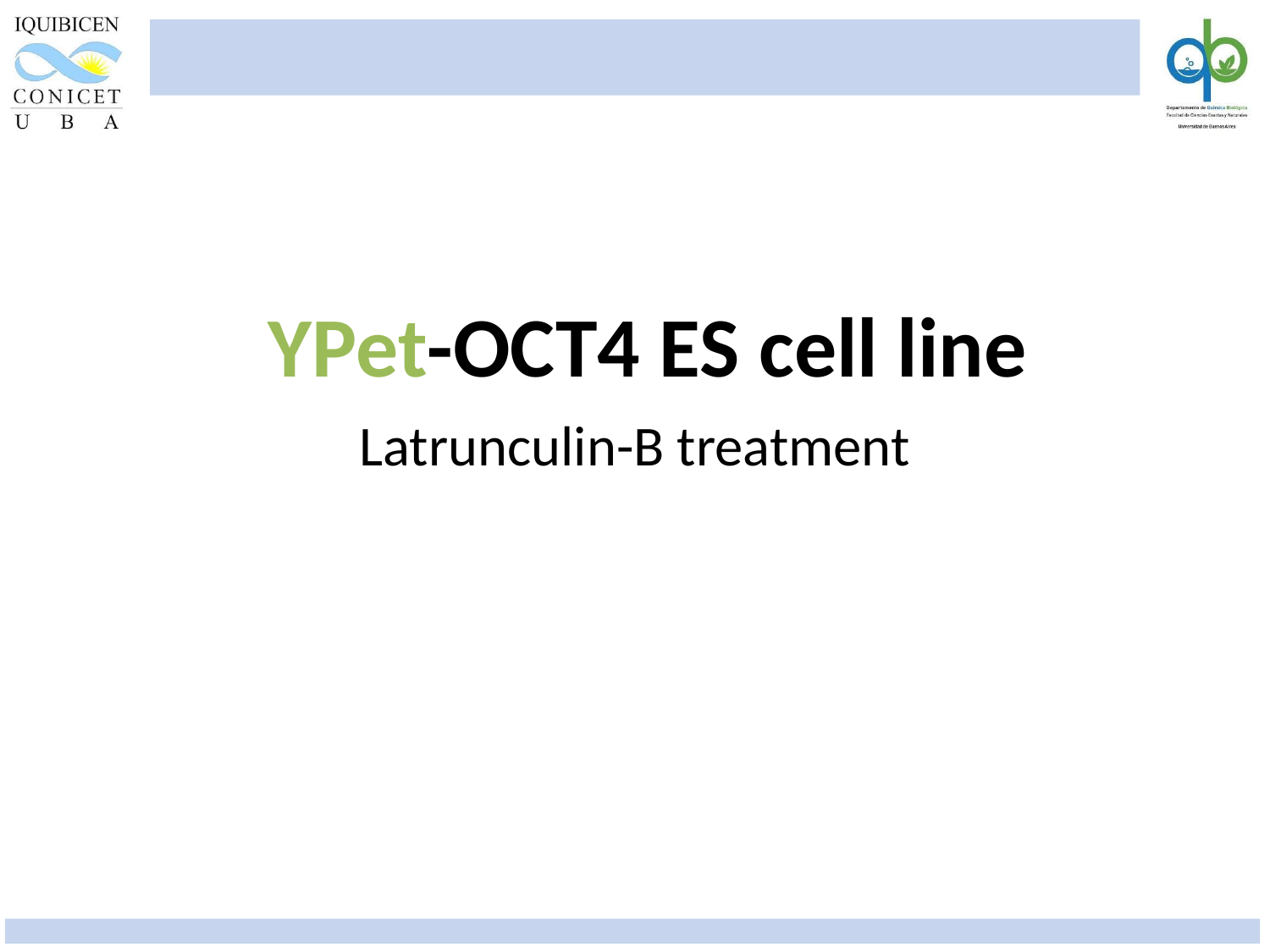

YPet-OCT4 ES cell line
Latrunculin-B treatment

## Slide 13
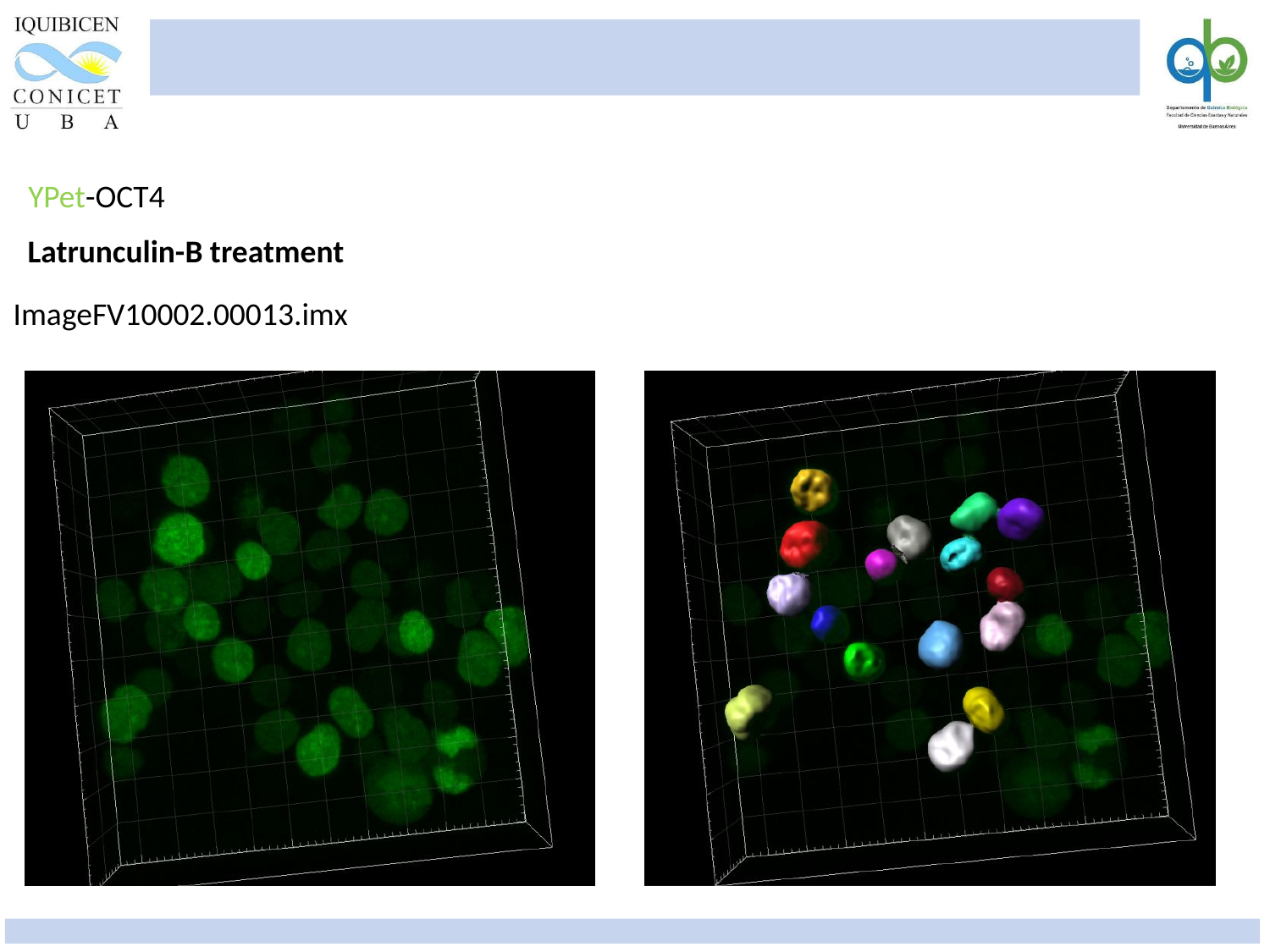

YPet-OCT4
Latrunculin-B treatment
ImageFV10002.00013.imx

## Slide 14
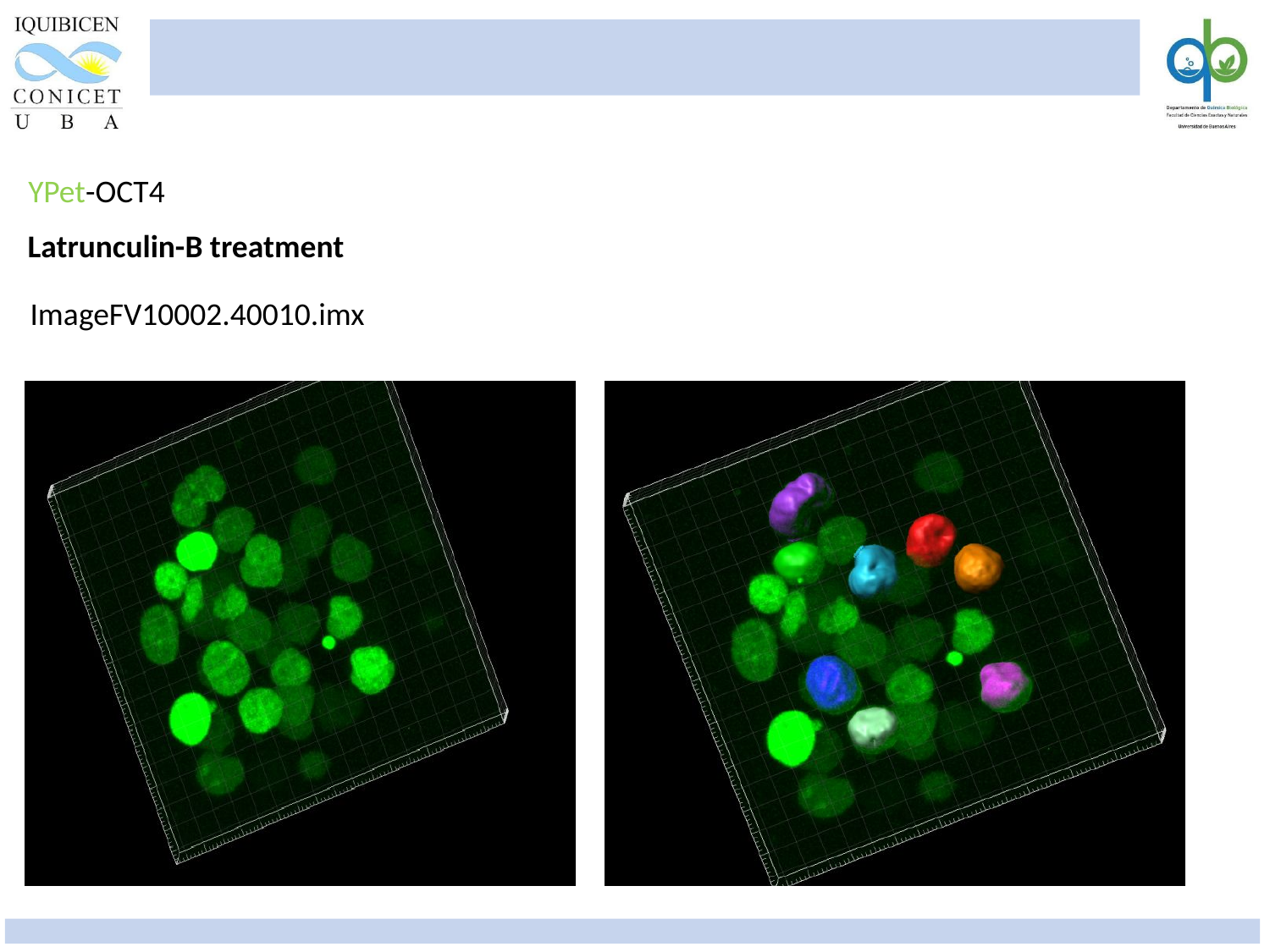

YPet-OCT4
Latrunculin-B treatment
ImageFV10002.40010.imx

## Slide 15
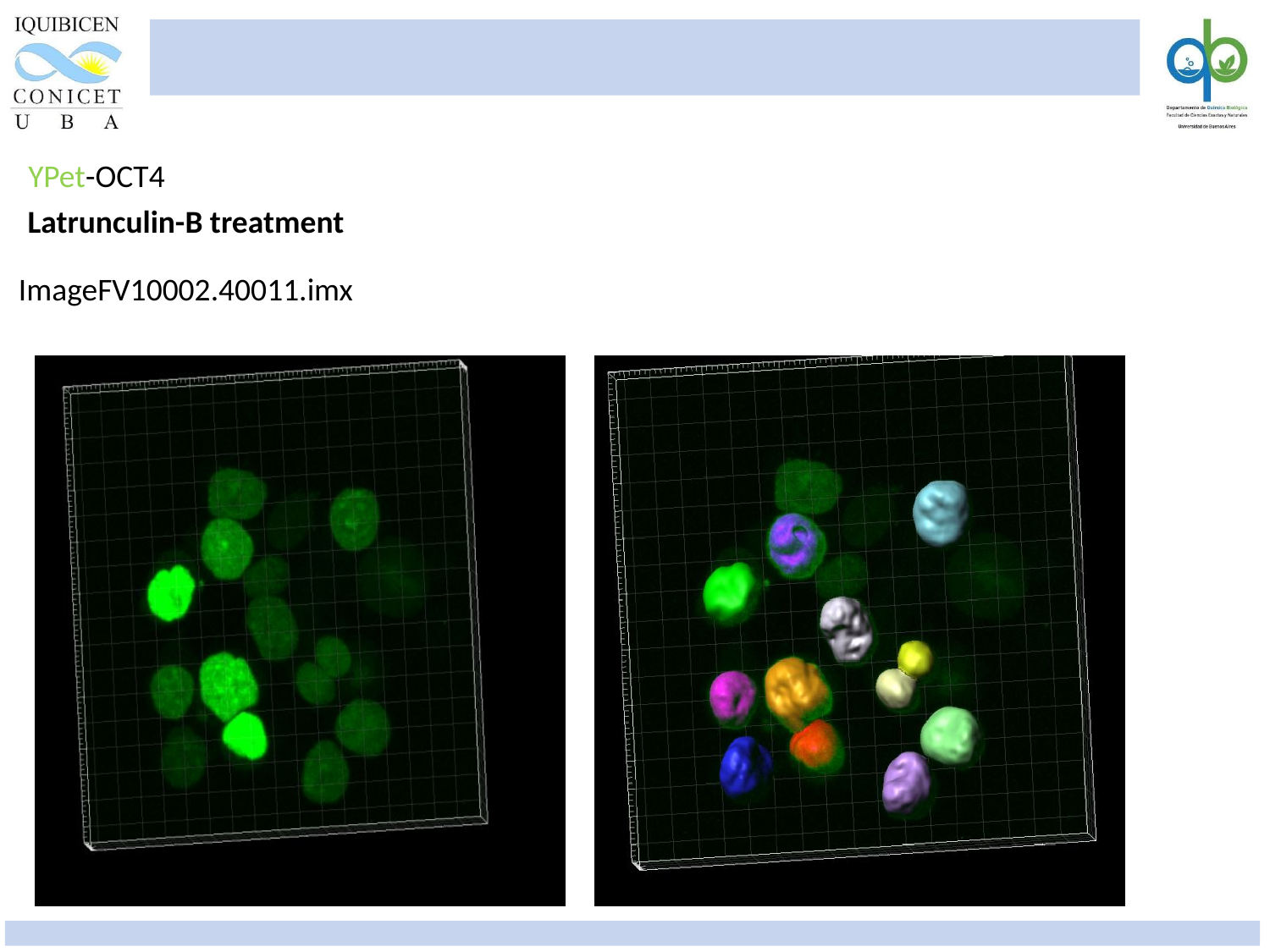

YPet-OCT4
Latrunculin-B treatment
ImageFV10002.40011.imx

## Slide 16
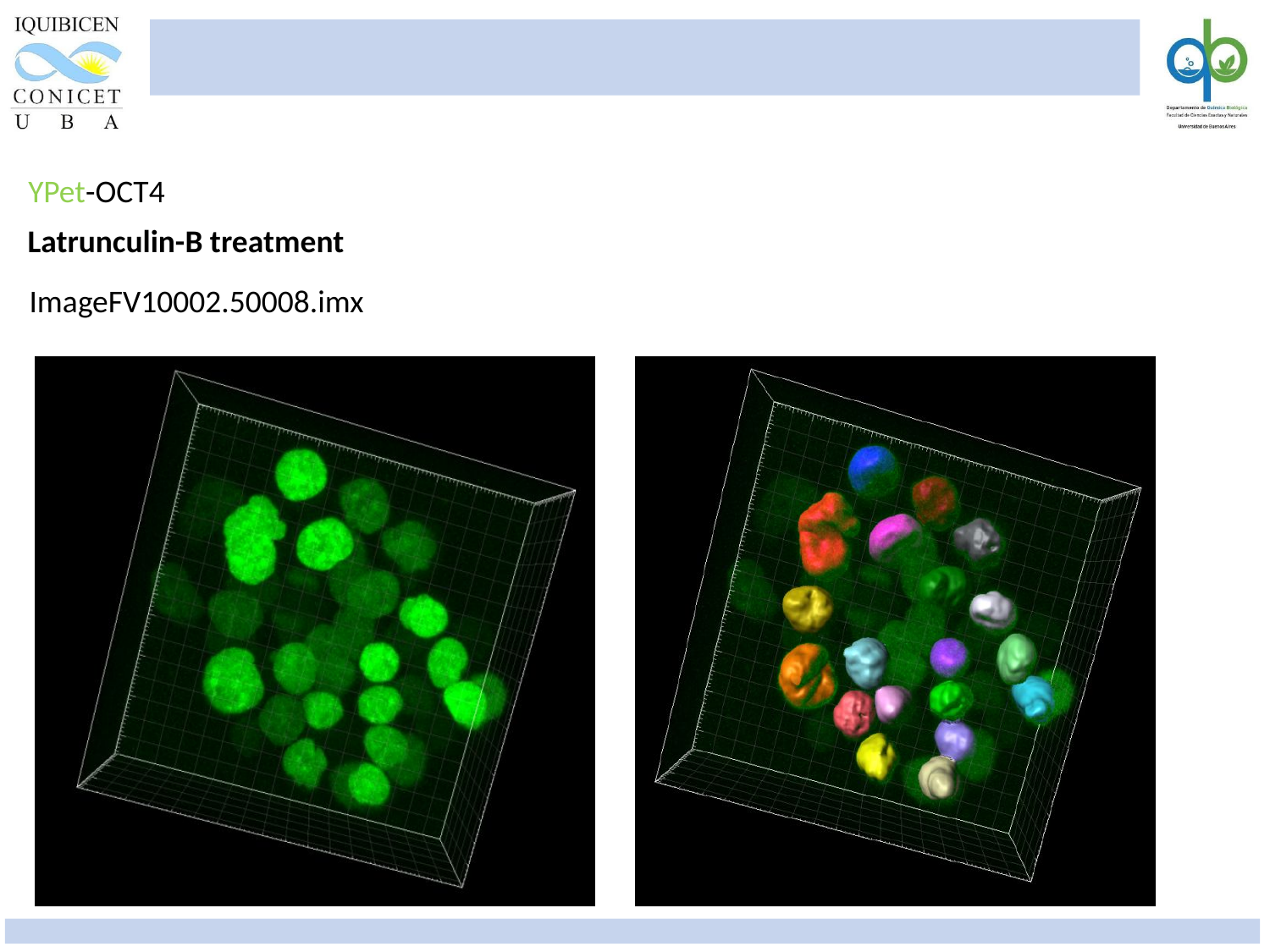

YPet-OCT4
Latrunculin-B treatment
ImageFV10002.50008.imx

## Slide 17
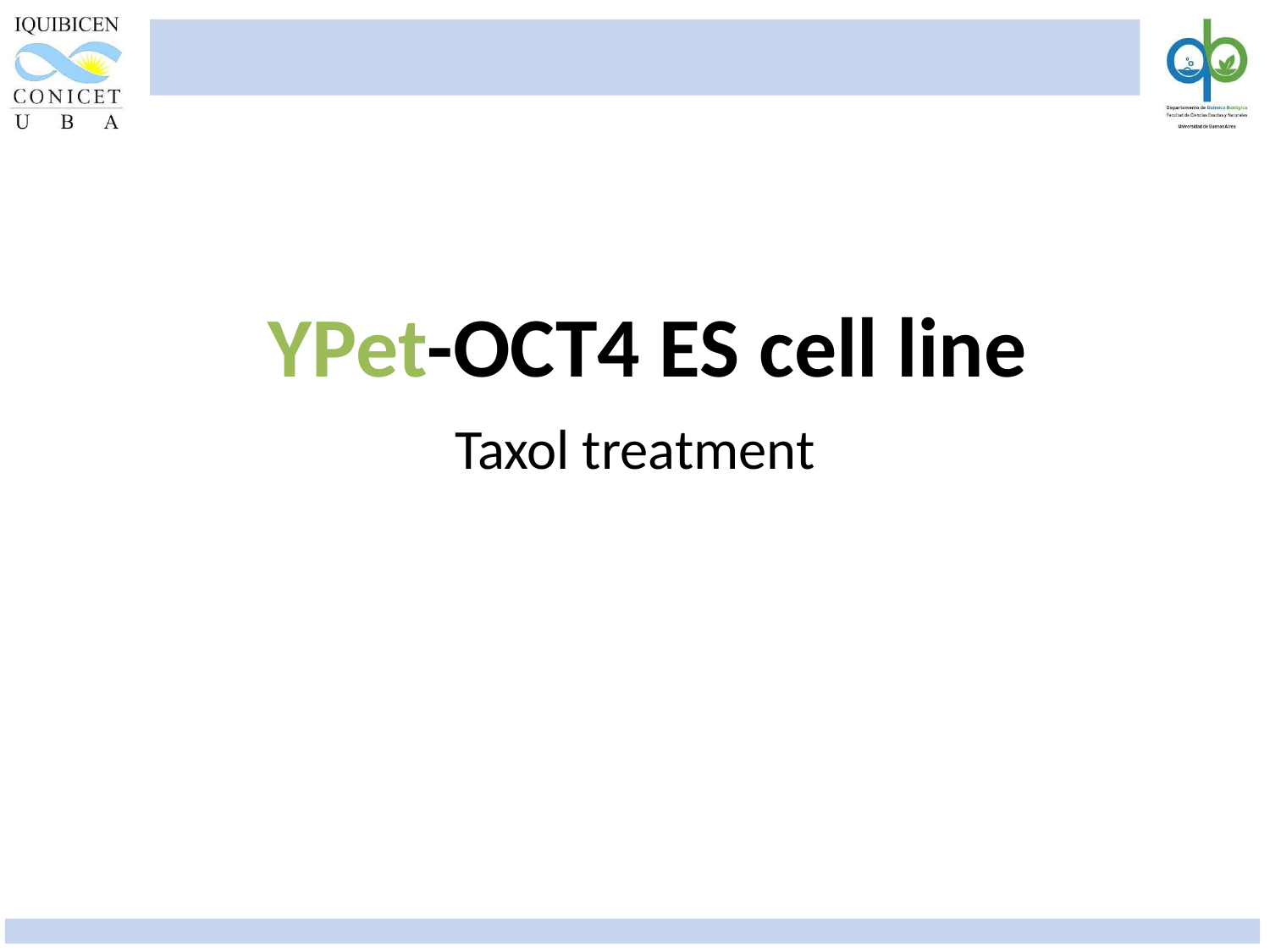

YPet-OCT4 ES cell line
Taxol treatment

## Slide 18
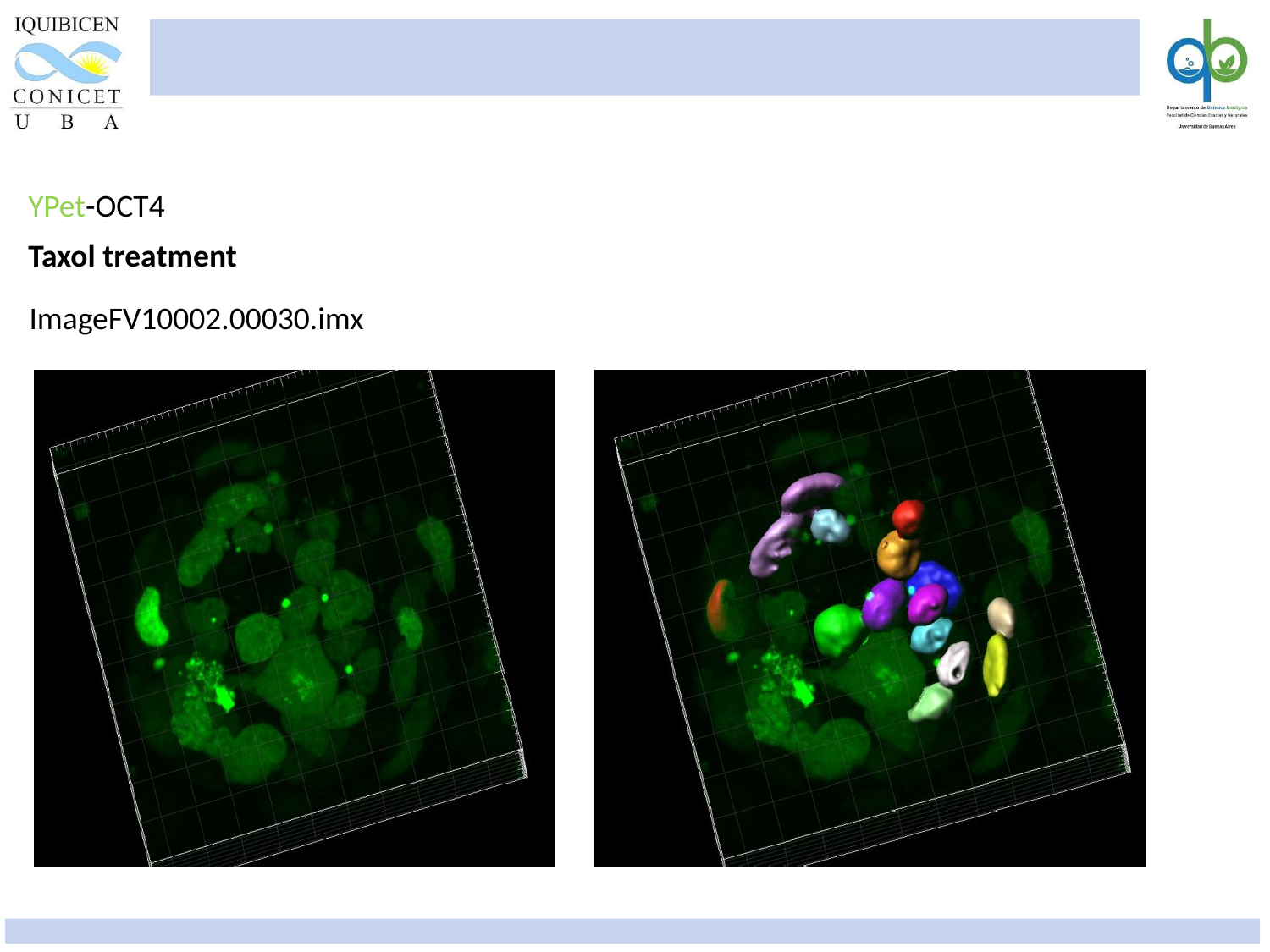

YPet-OCT4
Taxol treatment
ImageFV10002.00030.imx

## Slide 19
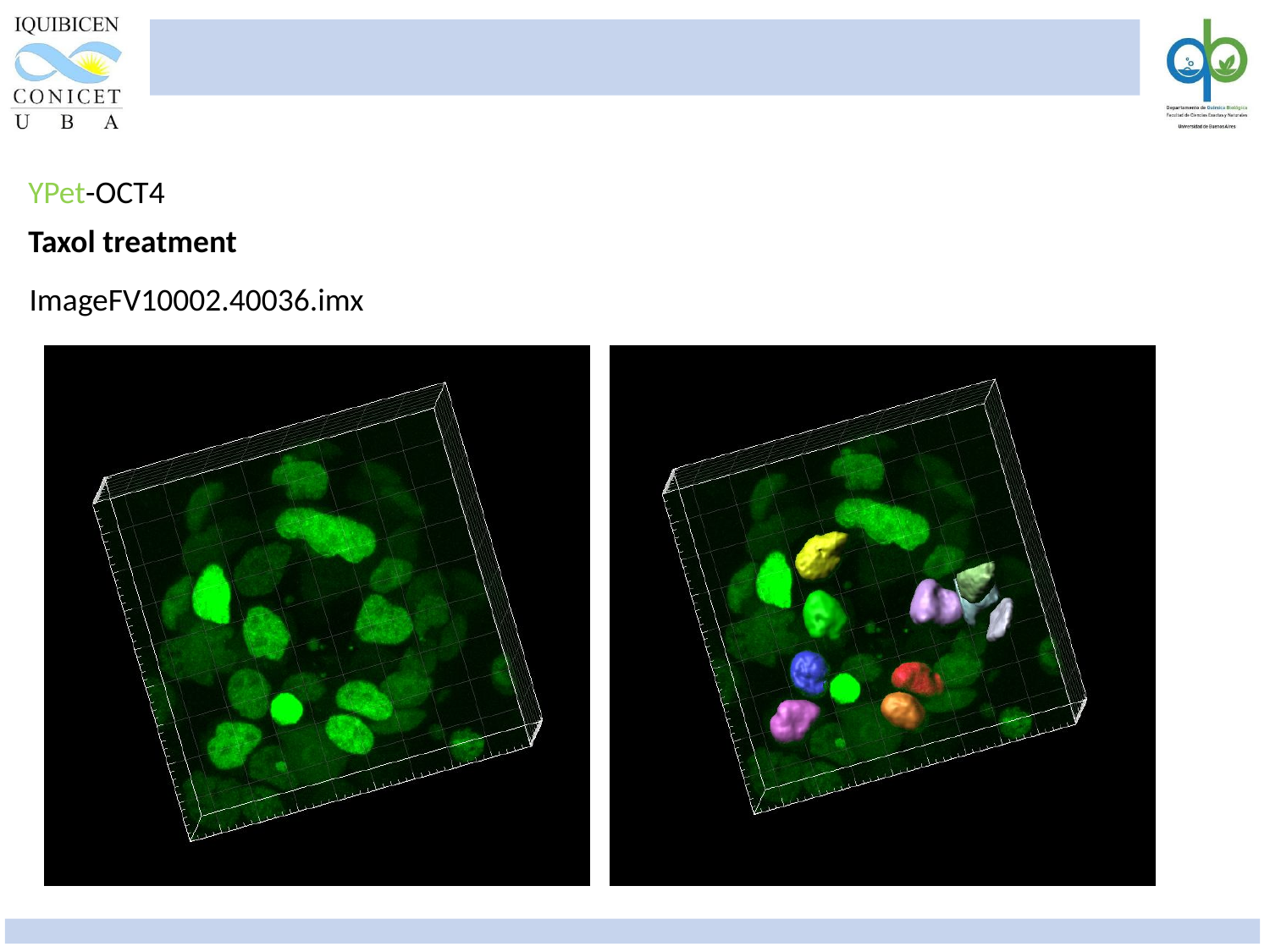

YPet-OCT4
Taxol treatment
ImageFV10002.40036.imx

## Slide 20
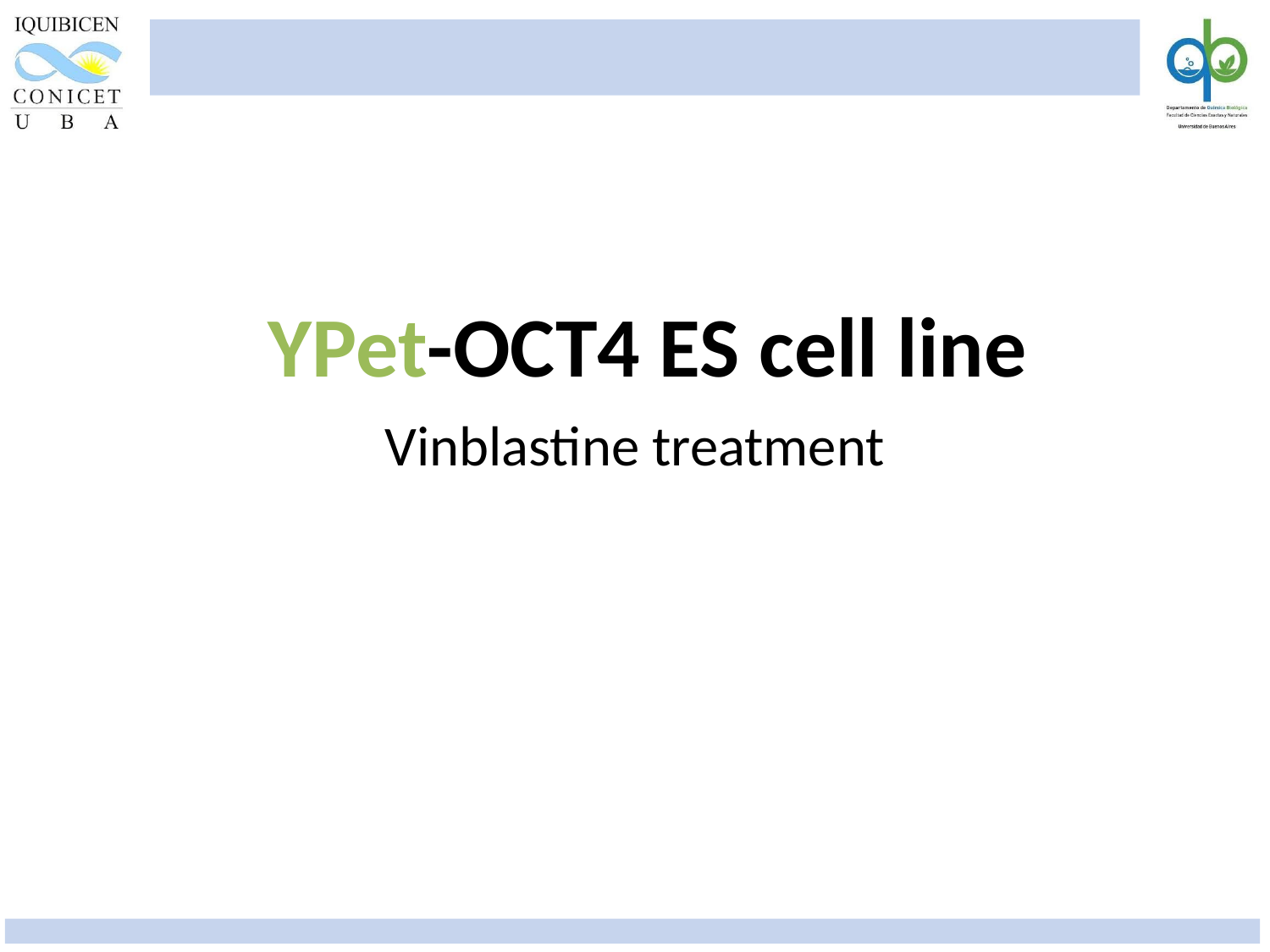

YPet-OCT4 ES cell line
Vinblastine treatment

## Slide 21
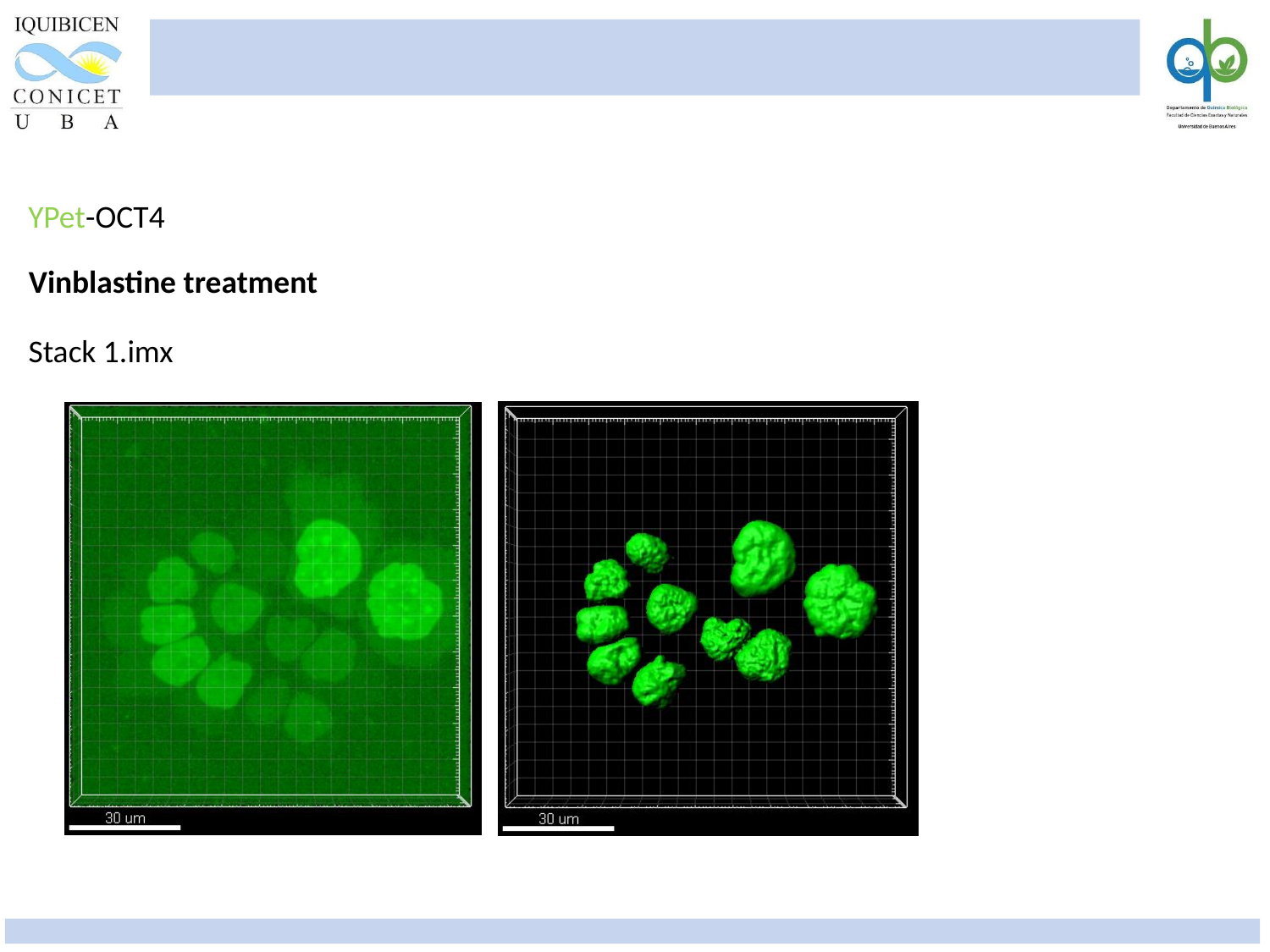

YPet-OCT4
Vinblastine treatment
Stack 1.imx

## Slide 22
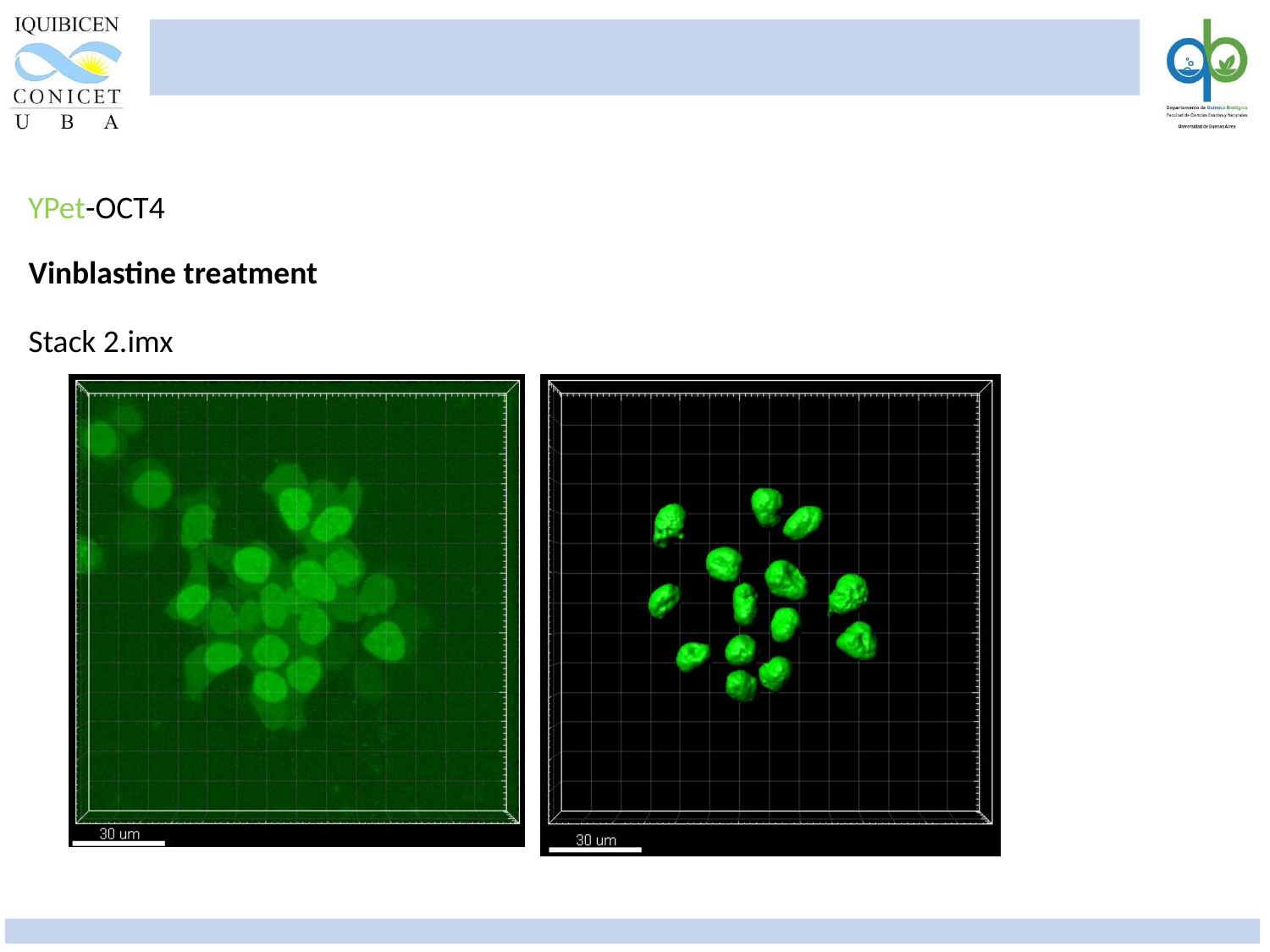

YPet-OCT4
Vinblastine treatment
Stack 2.imx

## Slide 23
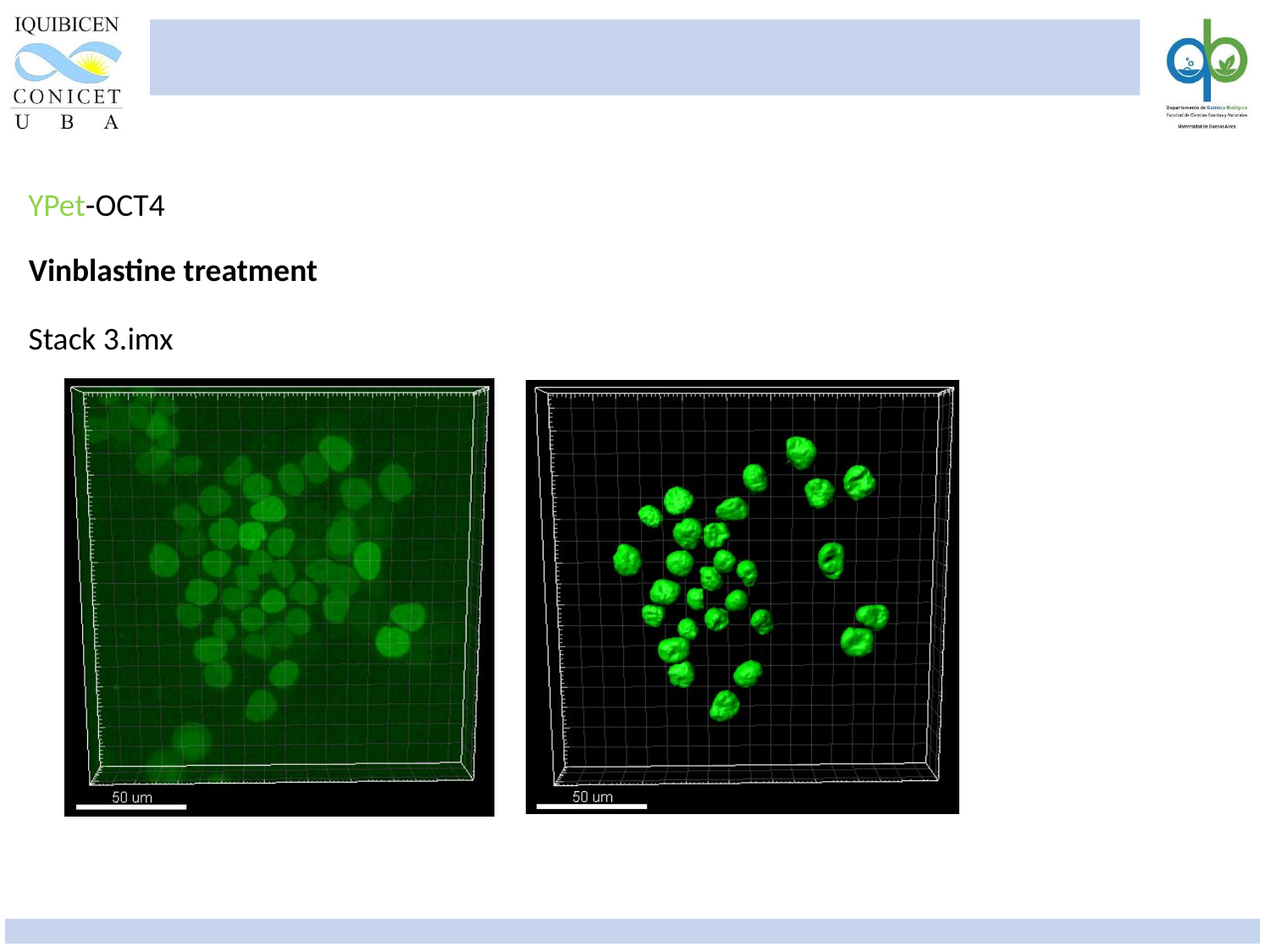

YPet-OCT4
Vinblastine treatment
Stack 3.imx

## Slide 24
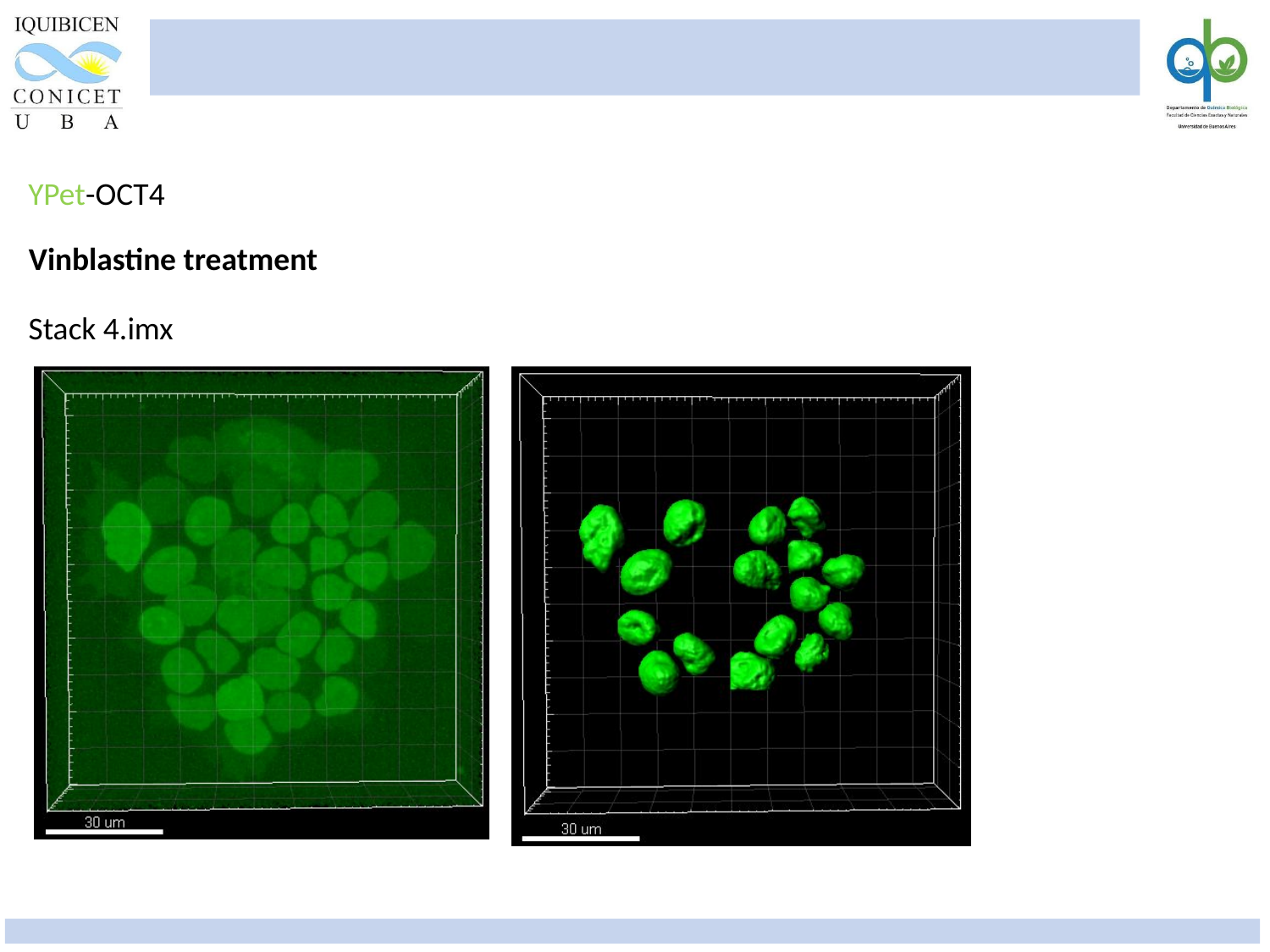

YPet-OCT4
Vinblastine treatment
Stack 4.imx

## Slide 25
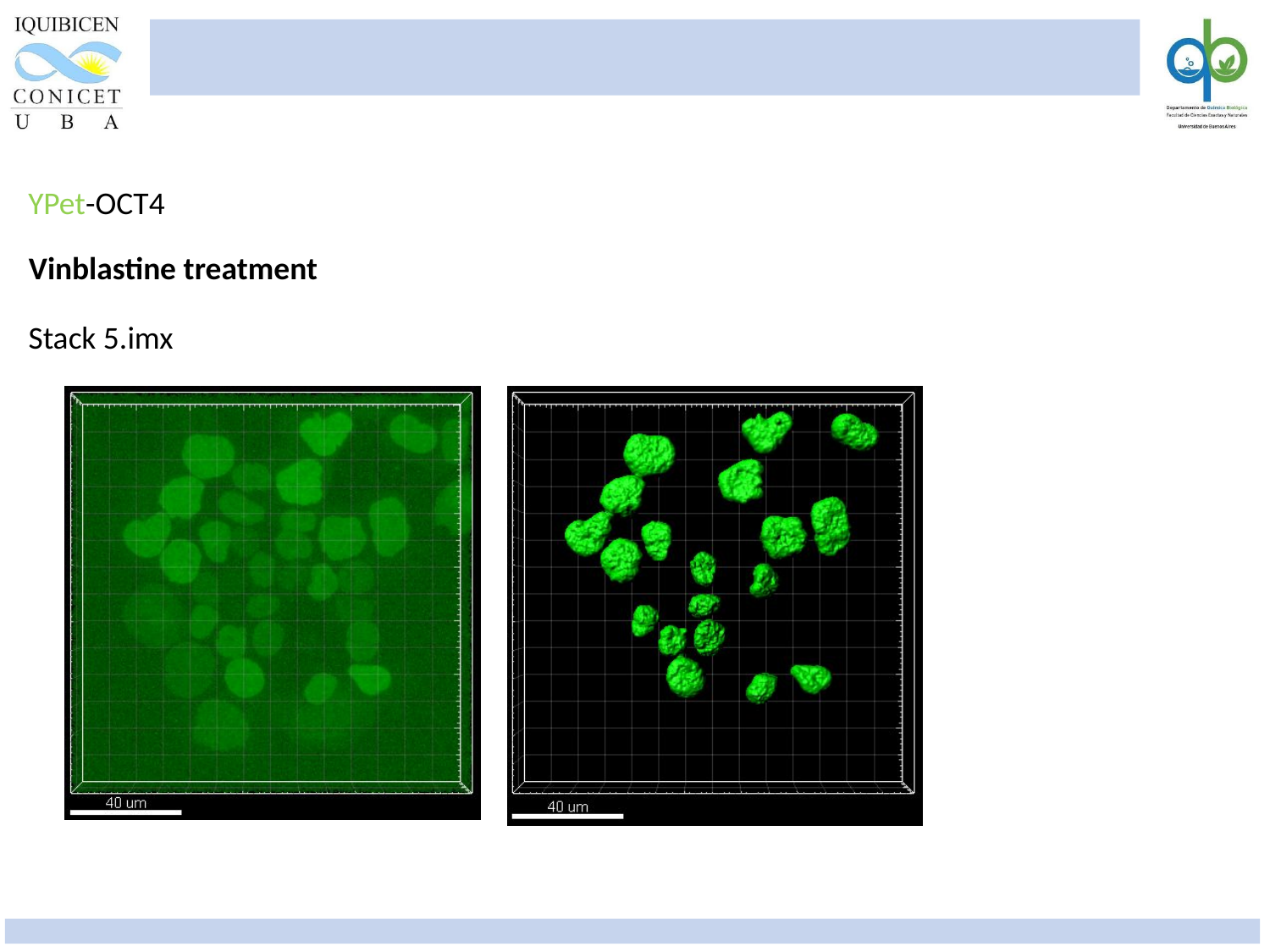

YPet-OCT4
Vinblastine treatment
Stack 5.imx

## Slide 26
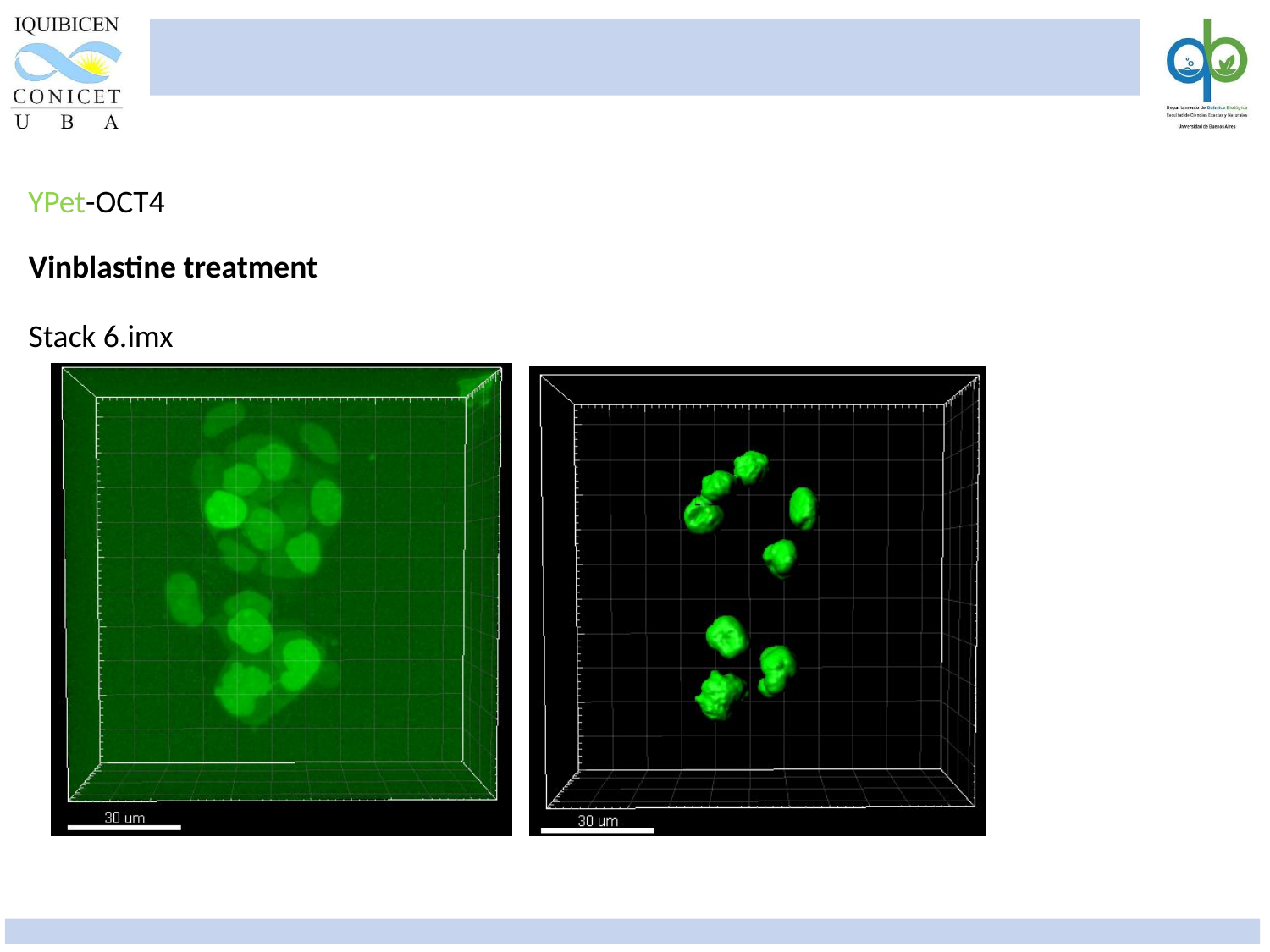

YPet-OCT4
Vinblastine treatment
Stack 6.imx

## Slide 27
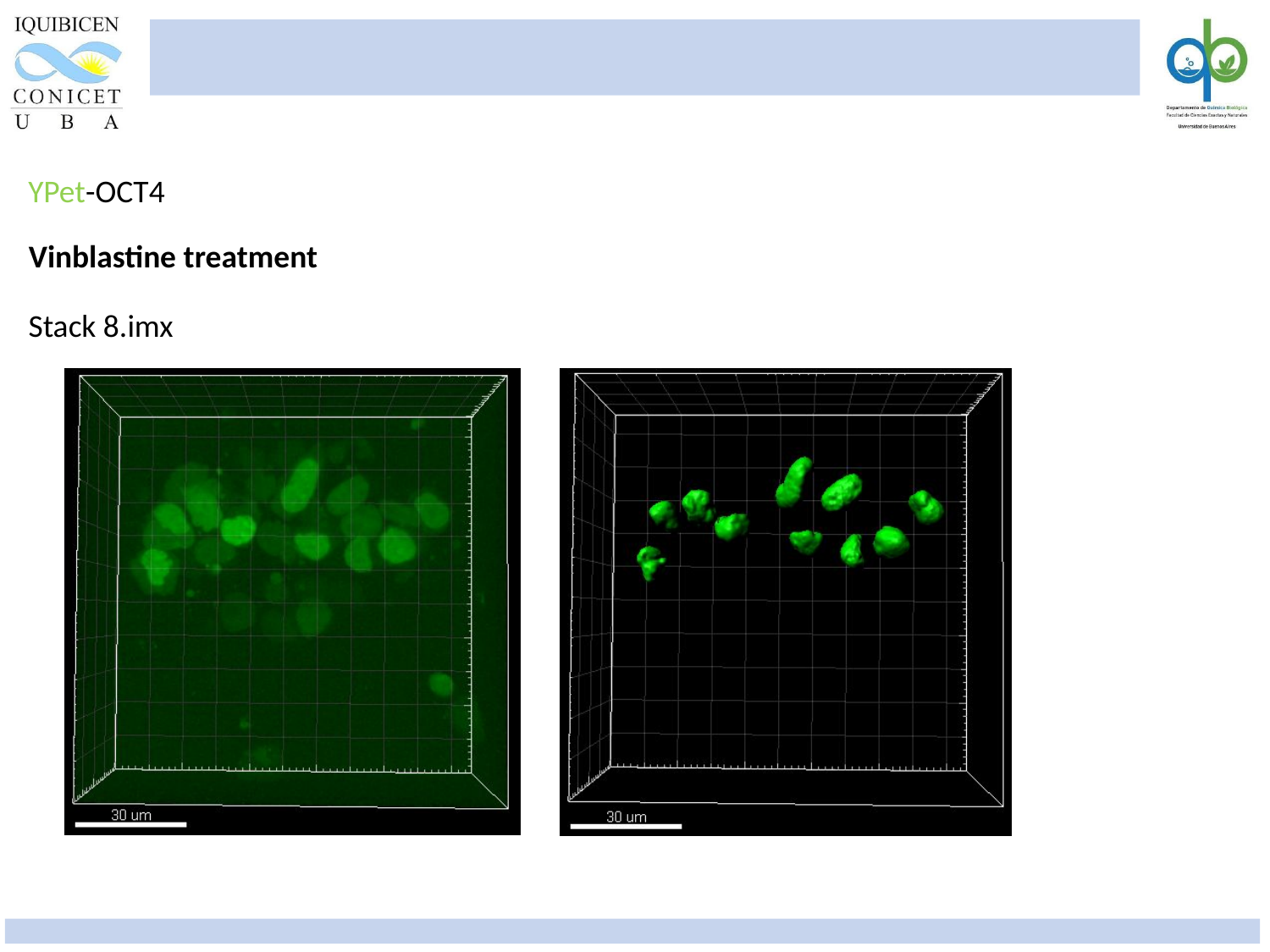

YPet-OCT4
Vinblastine treatment
Stack 8.imx

## Slide 28
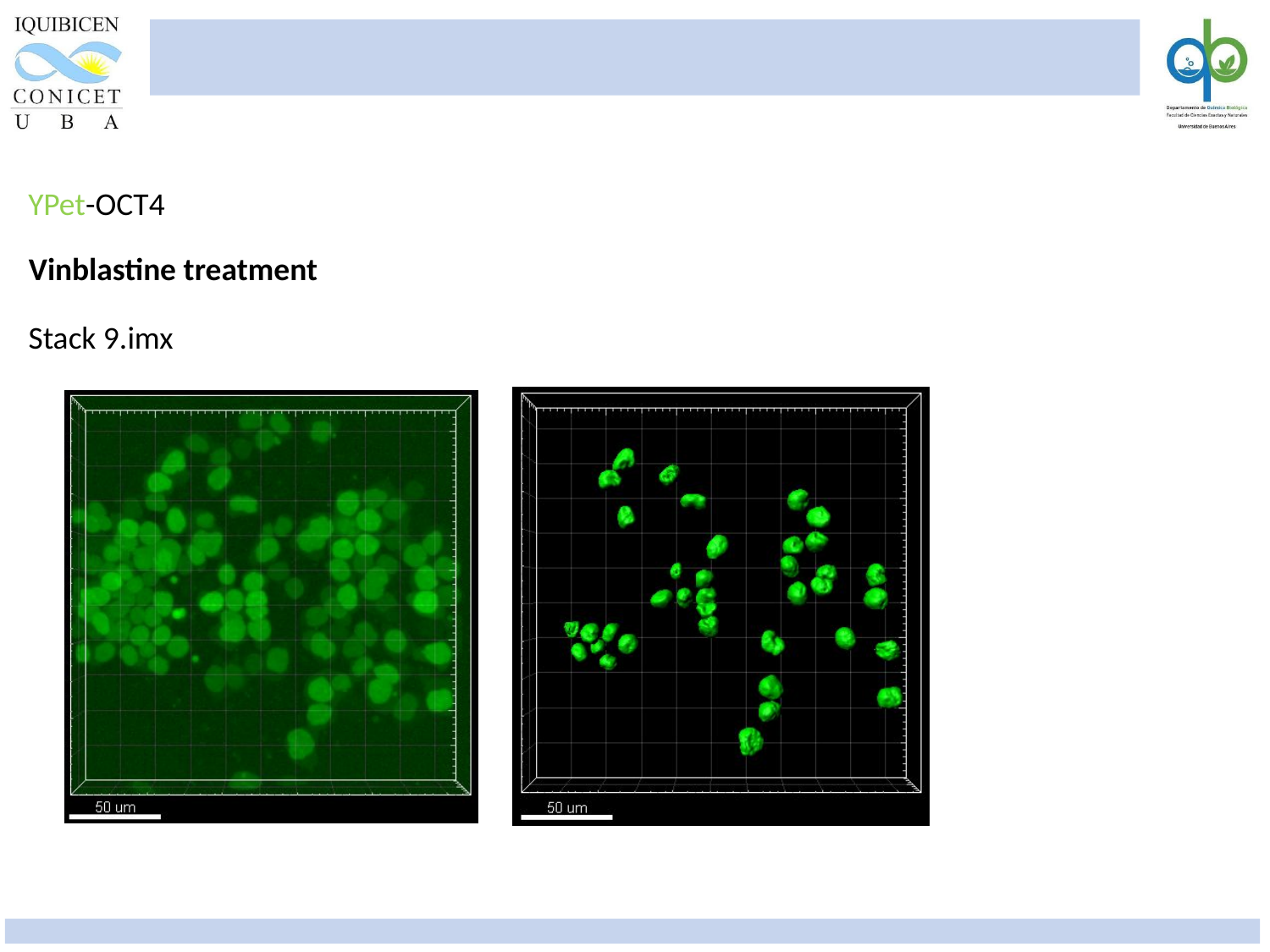

YPet-OCT4
Vinblastine treatment
Stack 9.imx

## Slide 29
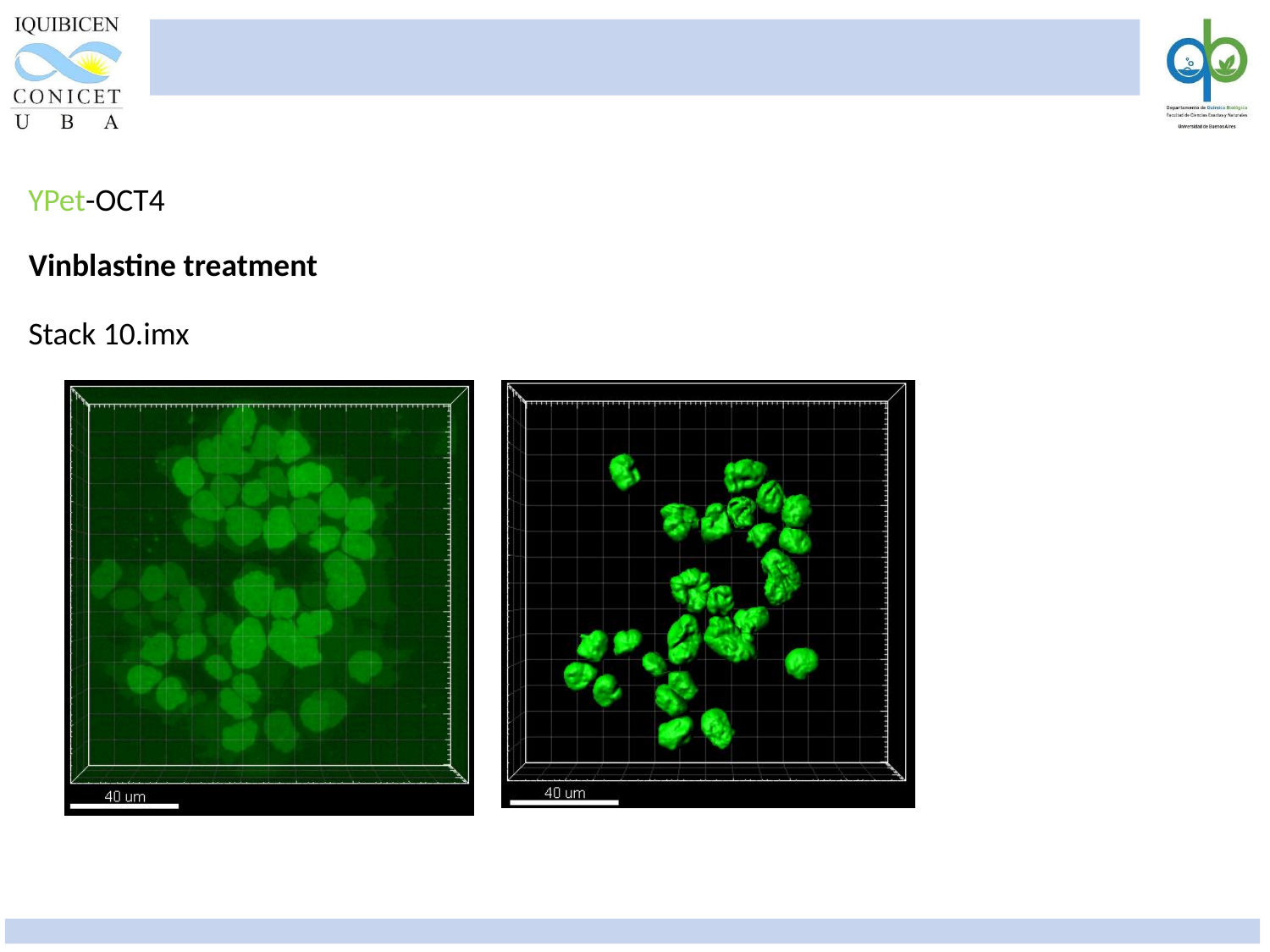

YPet-OCT4
Vinblastine treatment
Stack 10.imx

## Slide 30
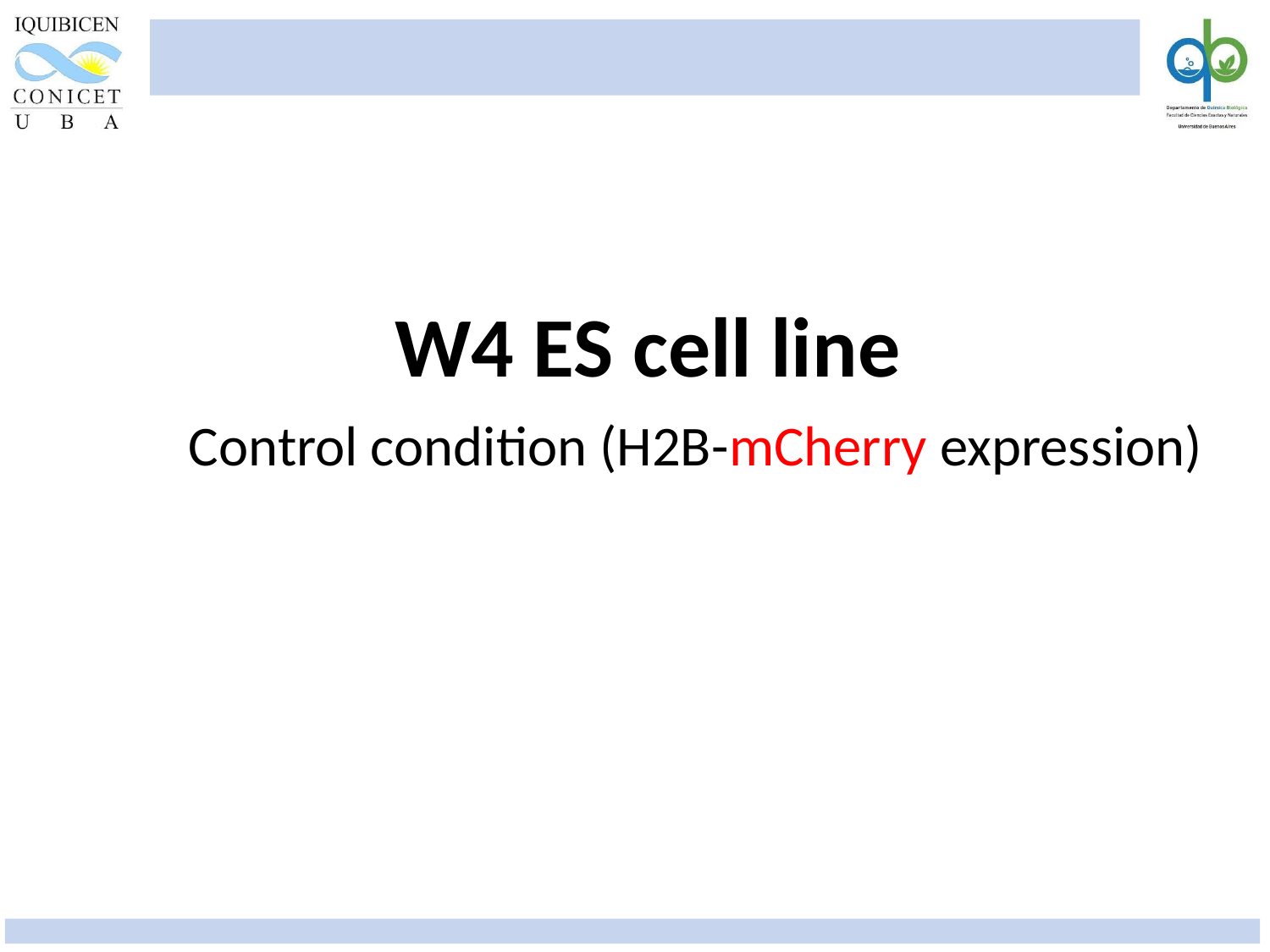

W4 ES cell line
Control condition (H2B-mCherry expression)

## Slide 31
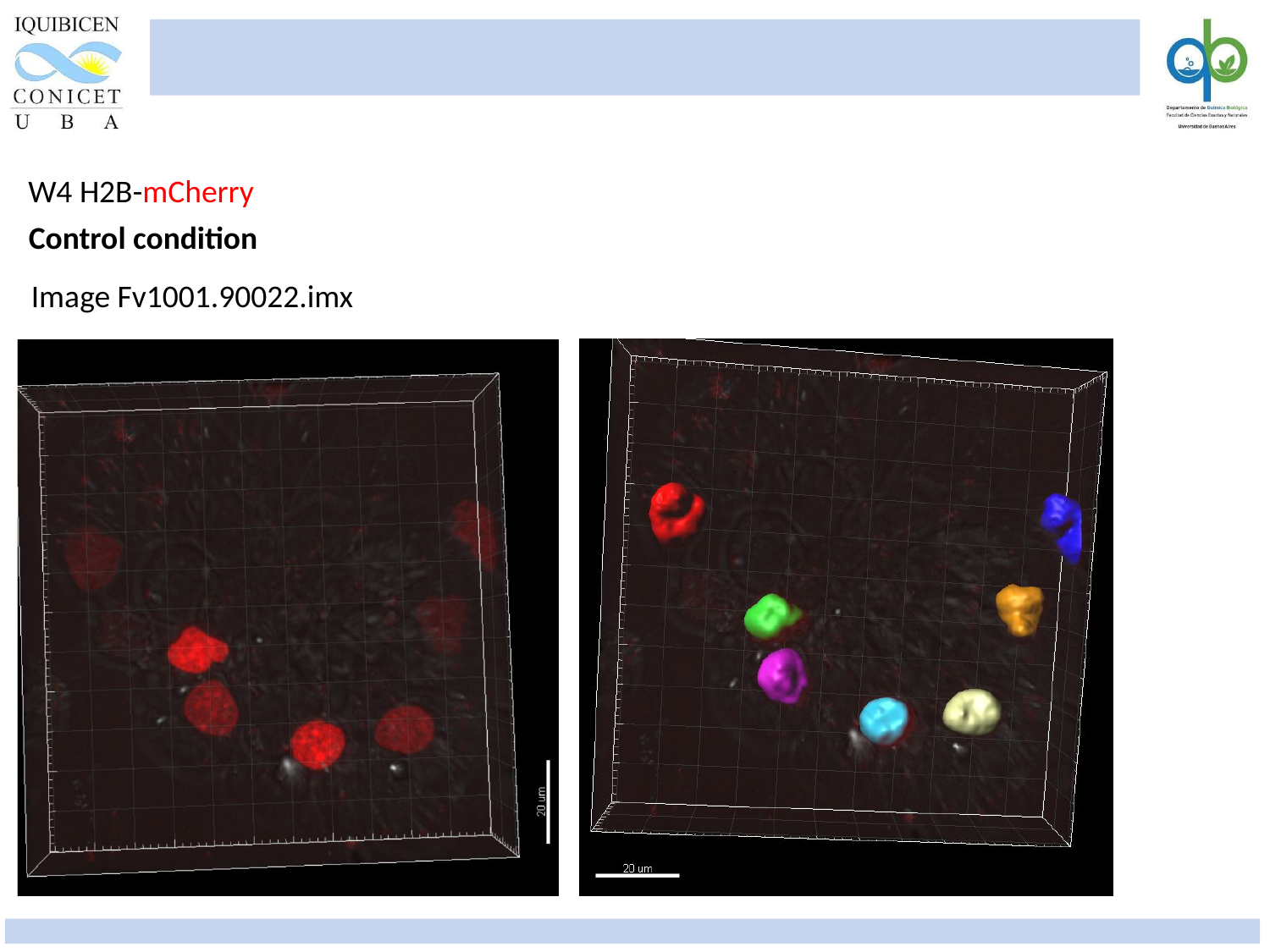

W4 H2B-mCherry
Control condition
Image Fv1001.90022.imx

## Slide 32
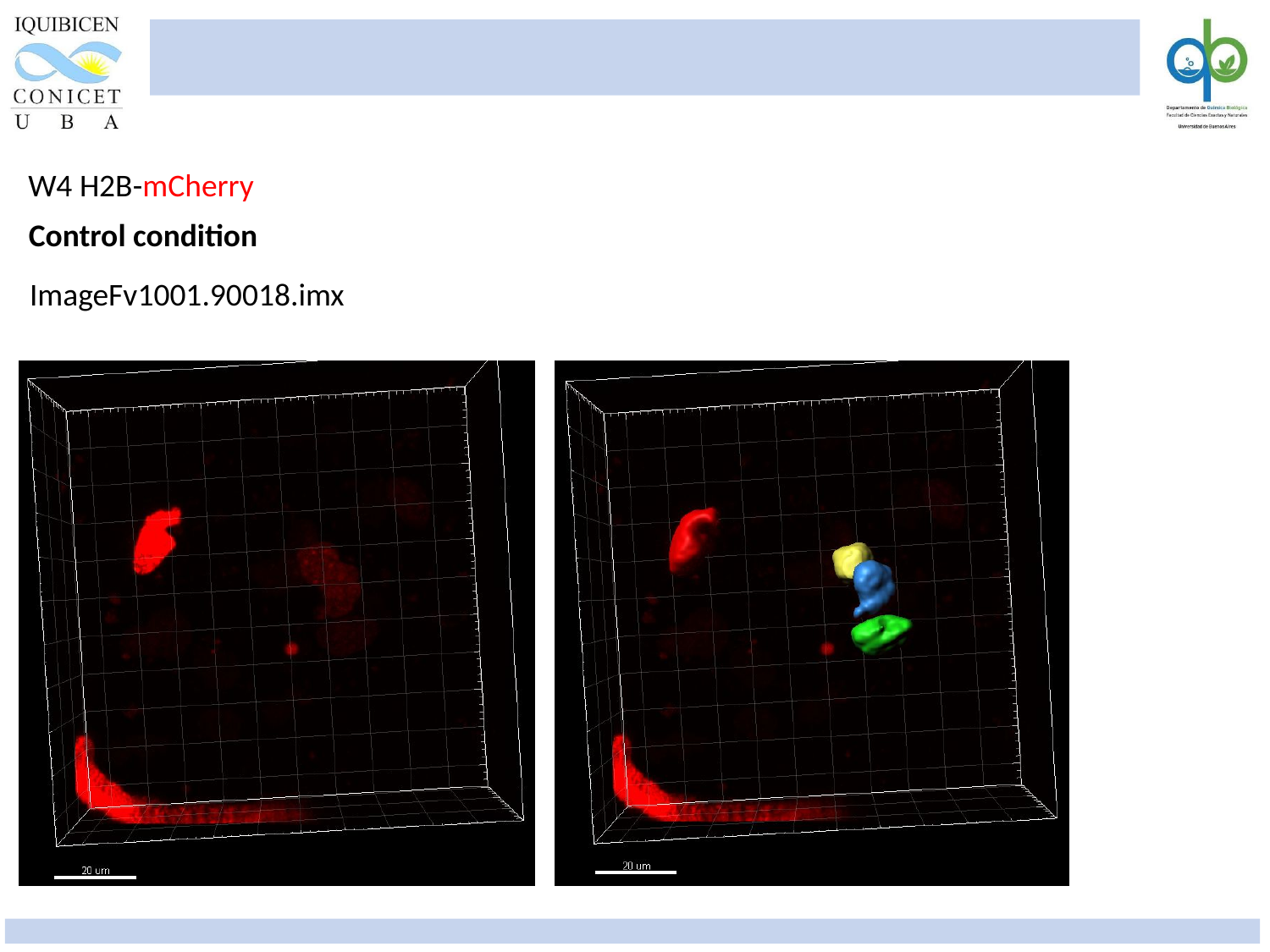

W4 H2B-mCherry
Control condition
ImageFv1001.90018.imx

## Slide 33
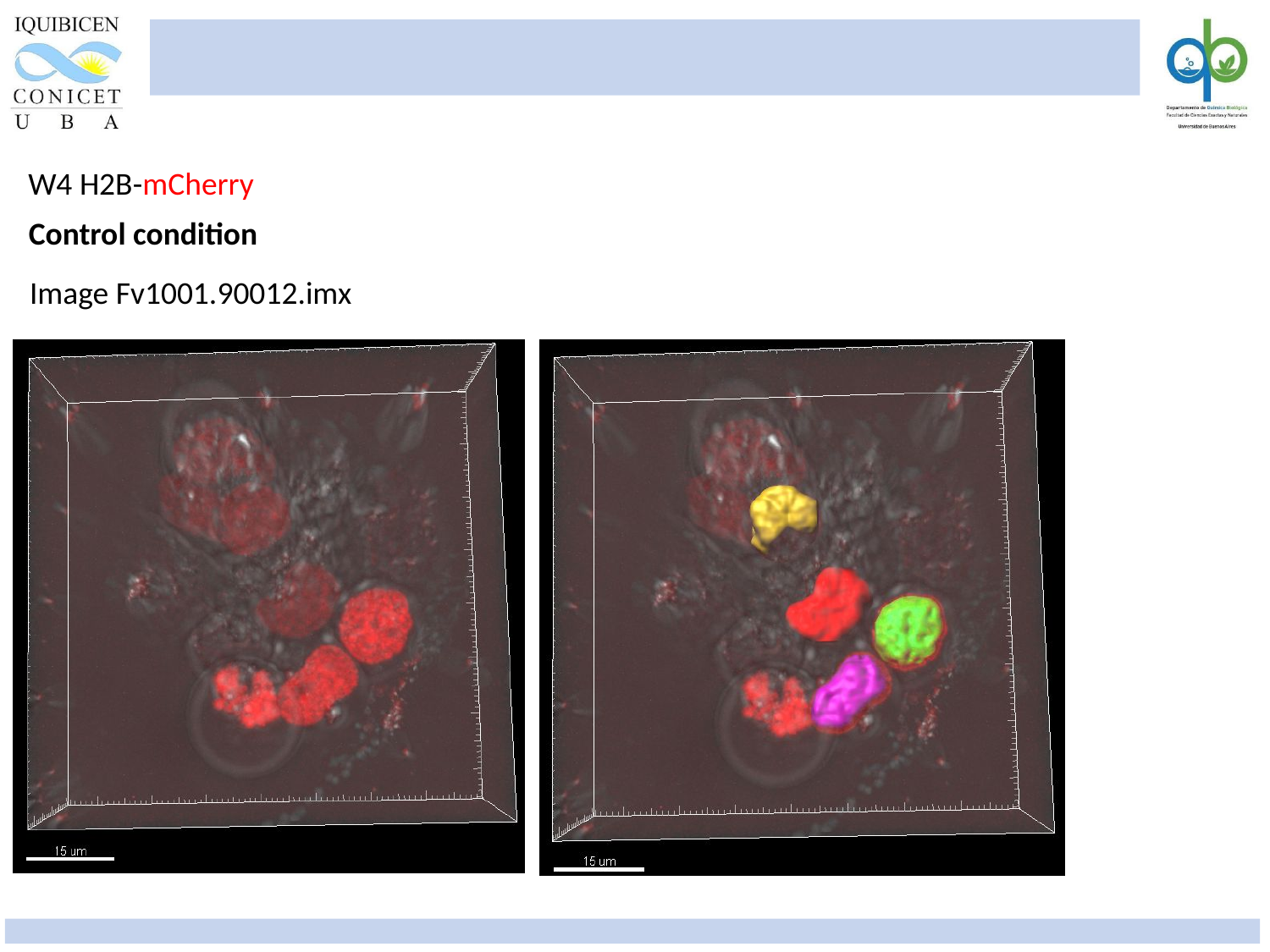

W4 H2B-mCherry
Control condition
Image Fv1001.90012.imx

## Slide 34
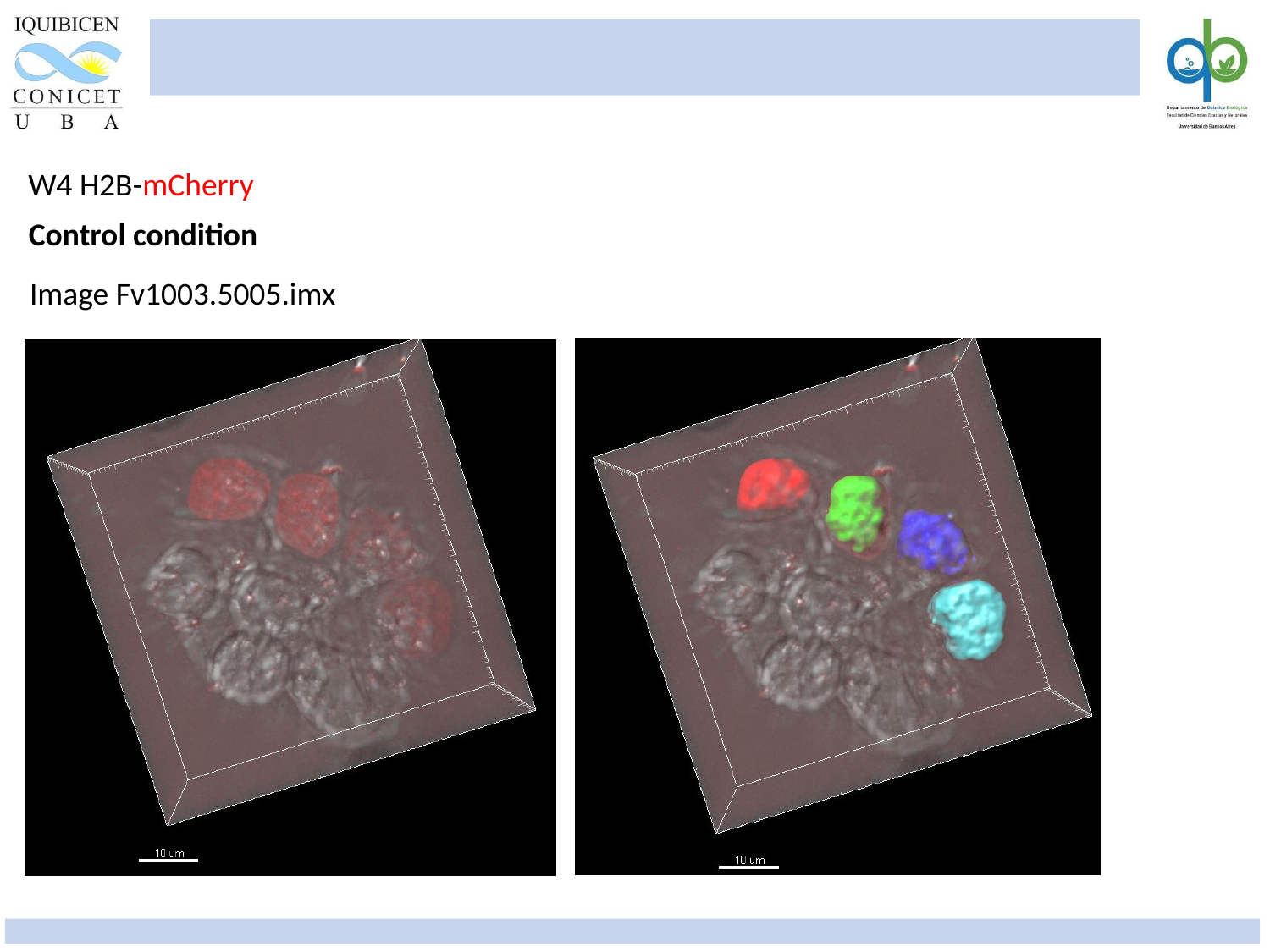

W4 H2B-mCherry
Control condition
Image Fv1003.5005.imx

## Slide 35
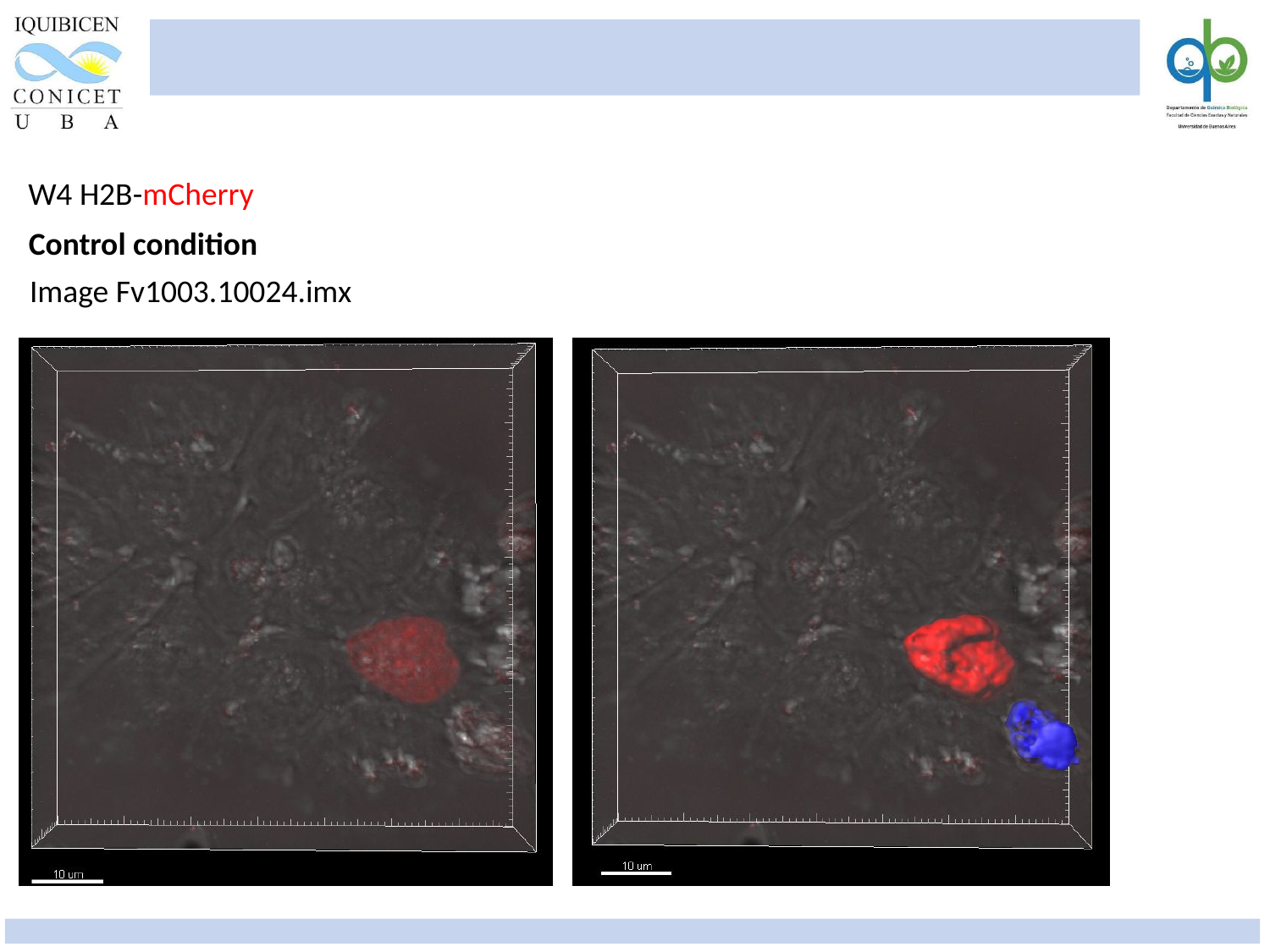

W4 H2B-mCherry
Control condition
Image Fv1003.10024.imx

## Slide 36
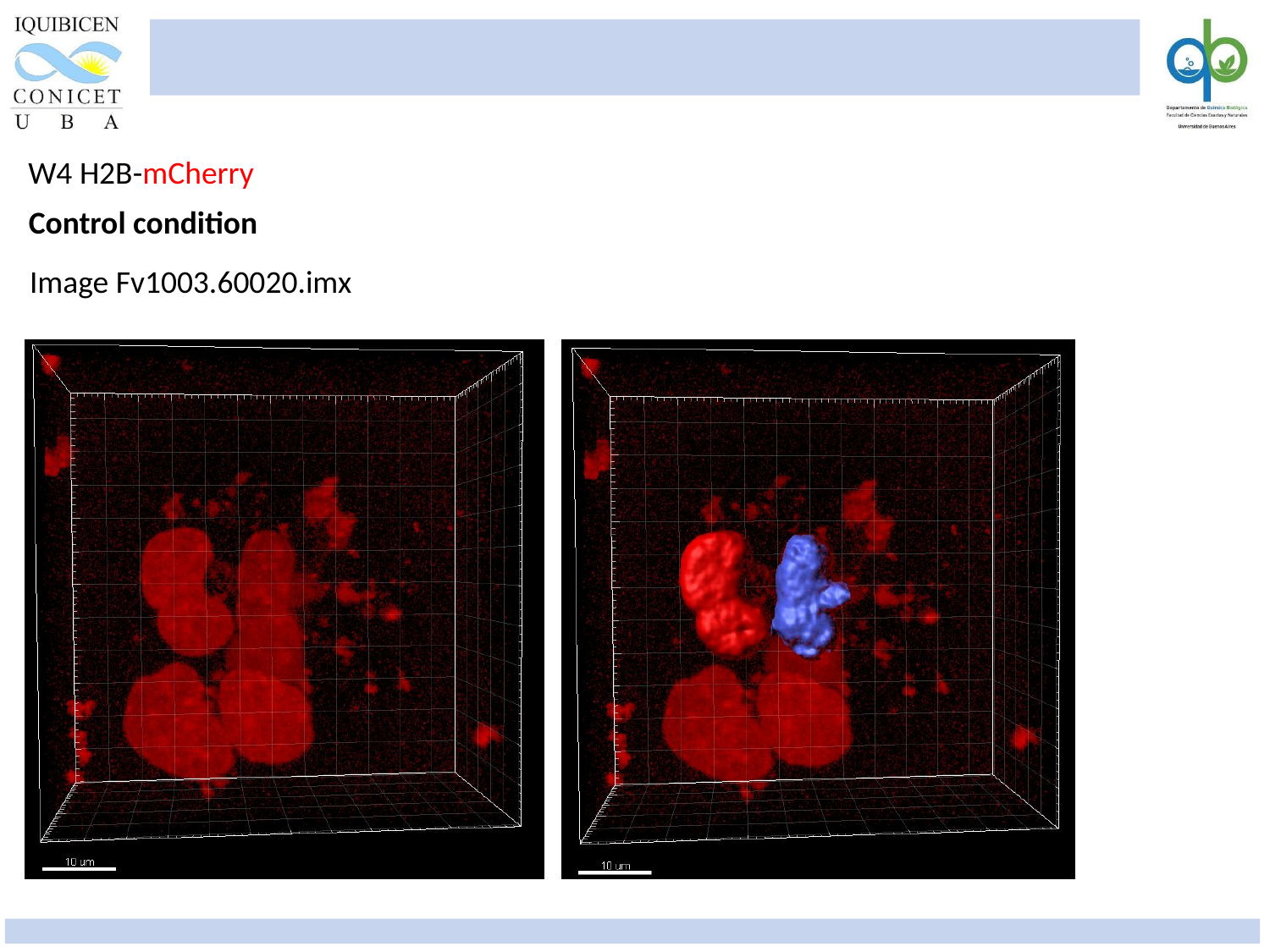

W4 H2B-mCherry
Control condition
Image Fv1003.60020.imx

## Slide 37
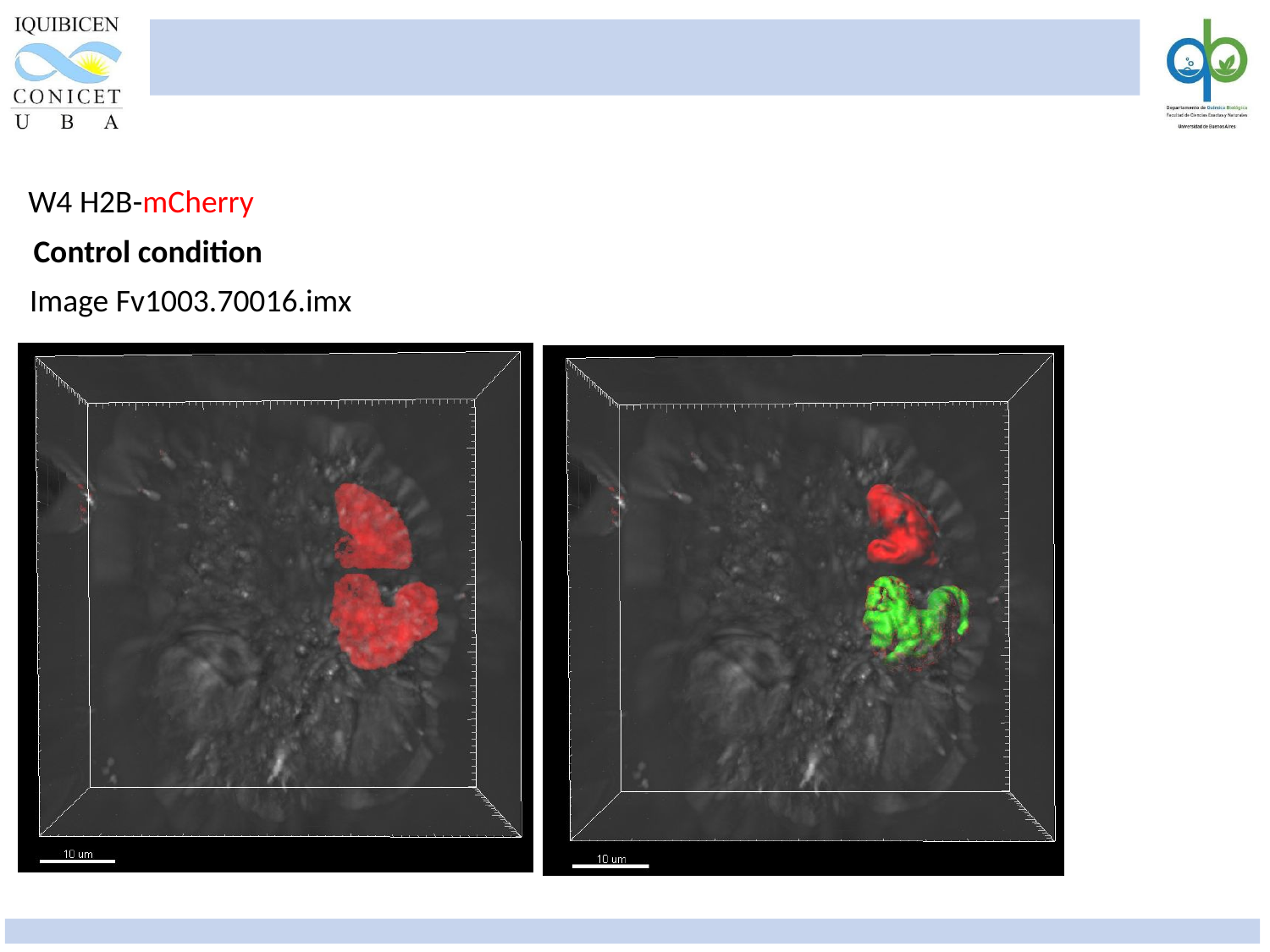

W4 H2B-mCherry
Control condition
Image Fv1003.70016.imx

## Slide 38
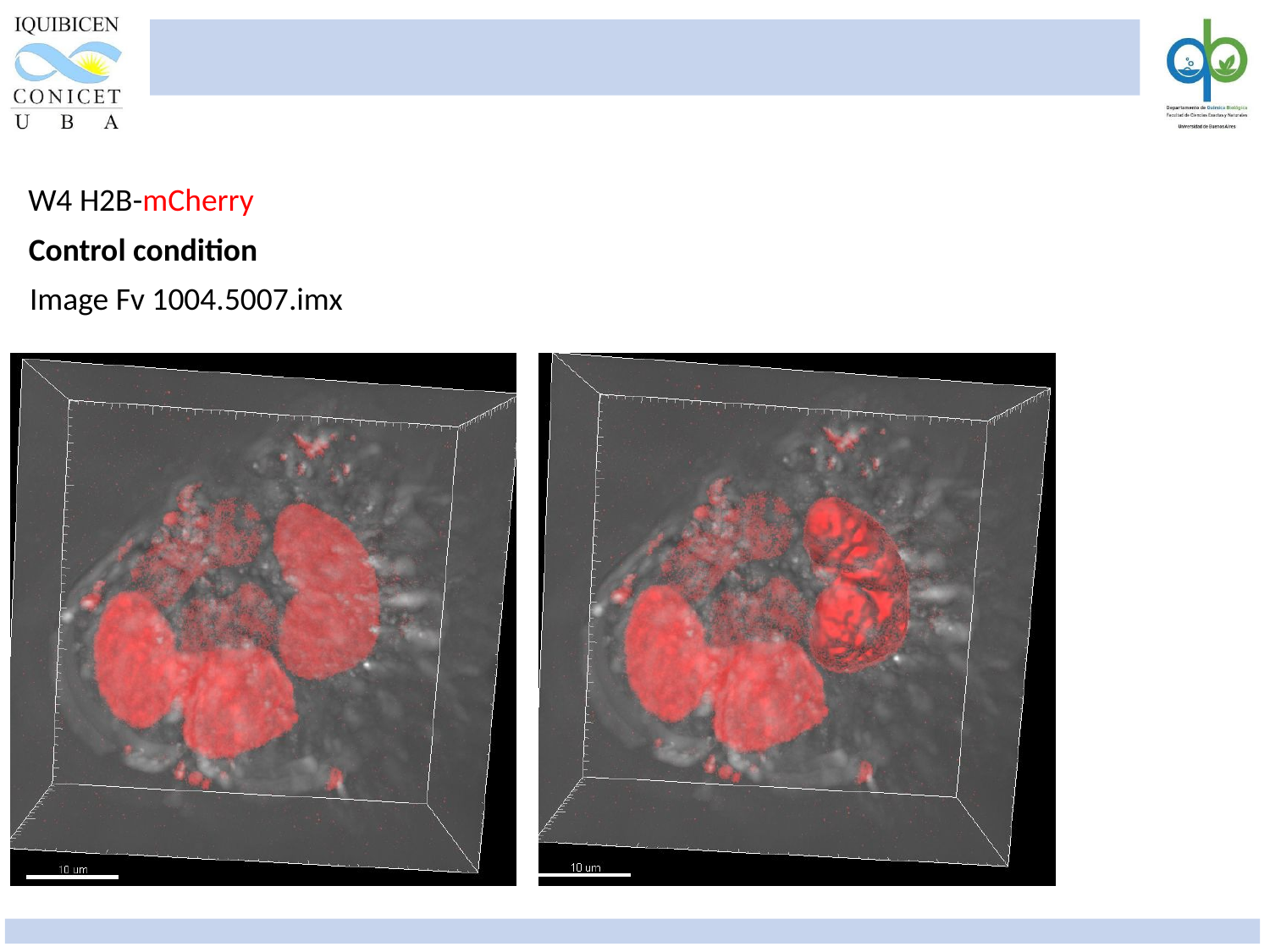

W4 H2B-mCherry
Control condition
Image Fv 1004.5007.imx

## Slide 39
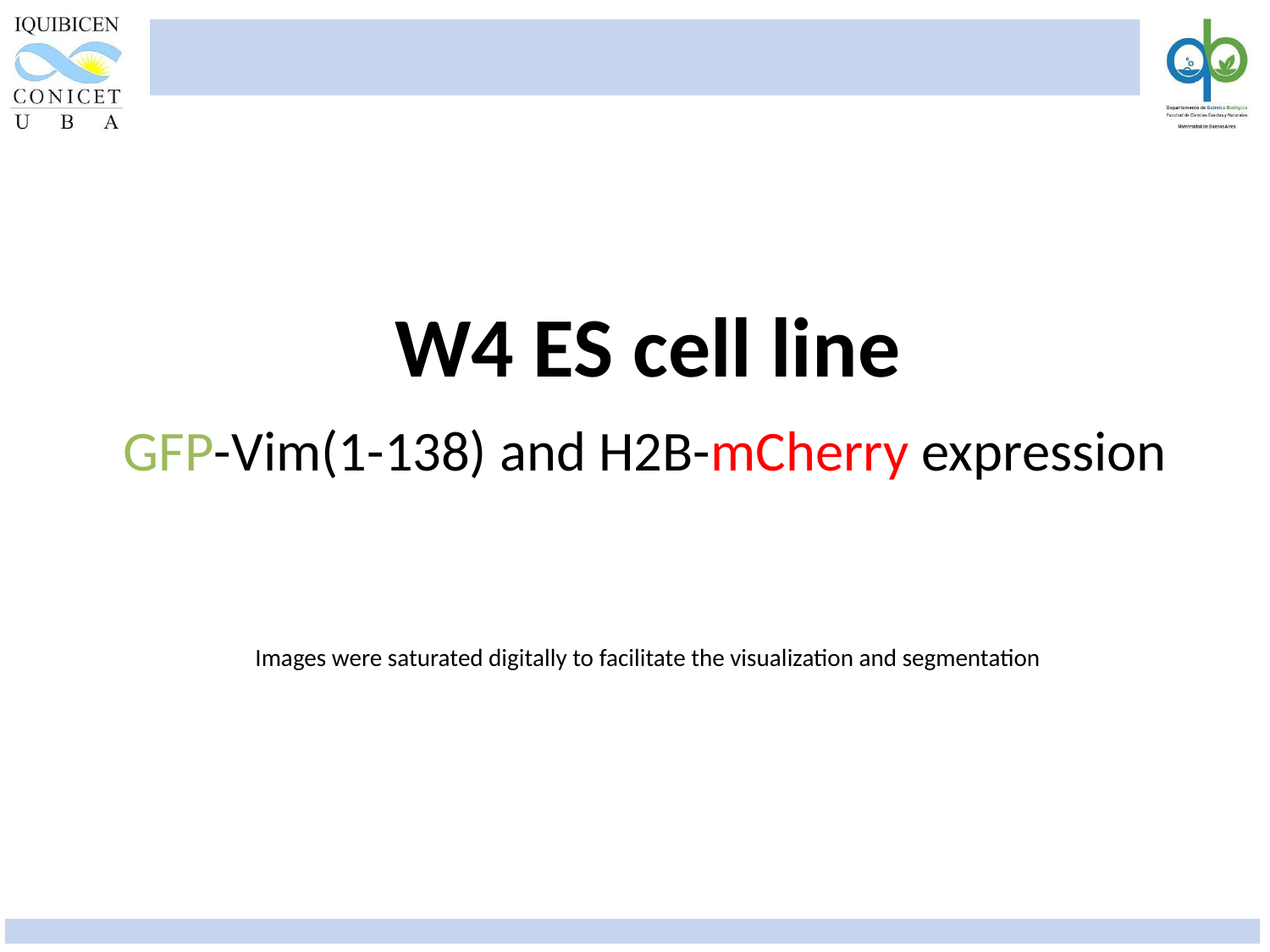

W4 ES cell line
GFP-Vim(1-138) and H2B-mCherry expression
Images were saturated digitally to facilitate the visualization and segmentation

## Slide 40
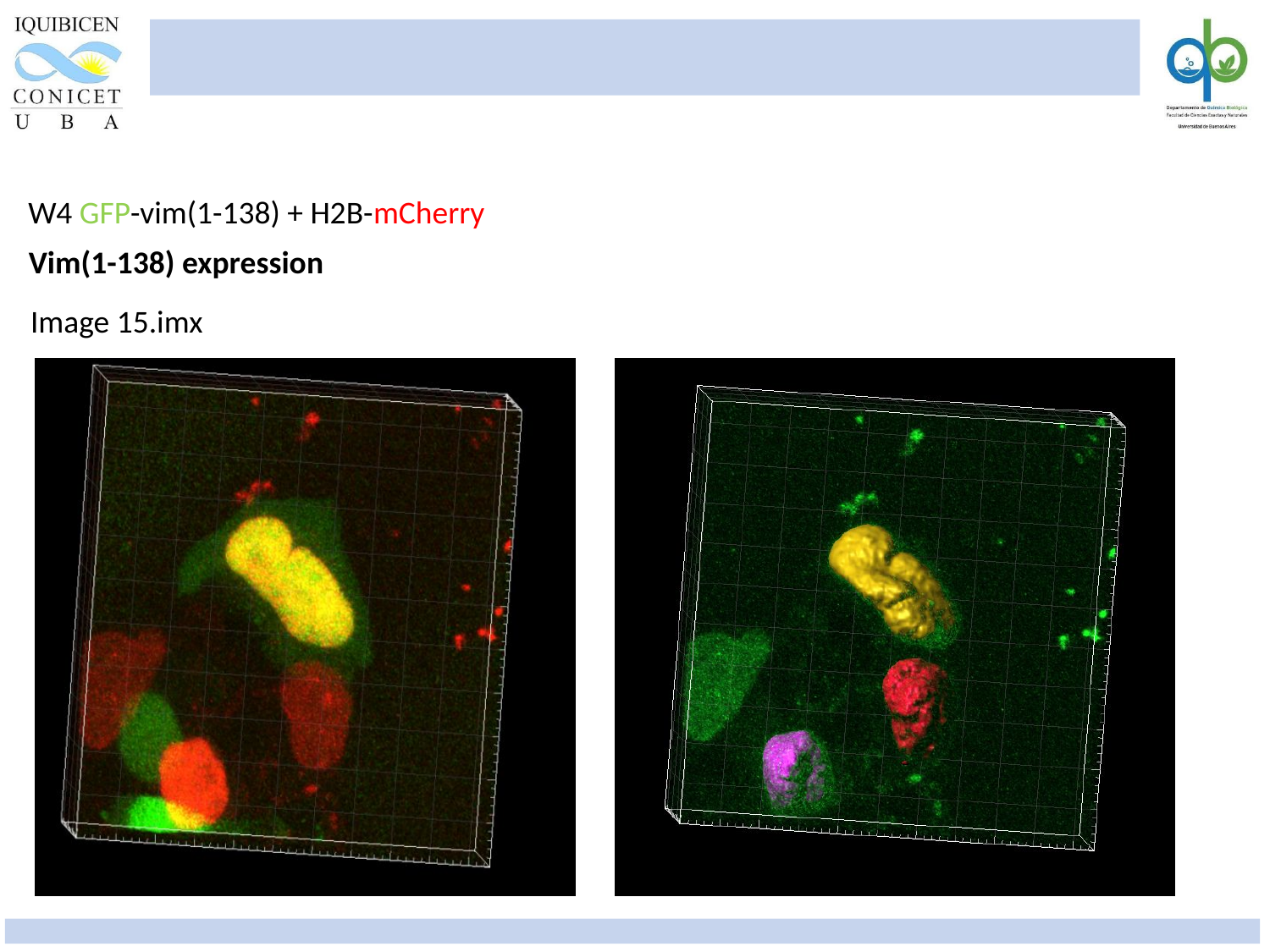

W4 GFP-vim(1-138) + H2B-mCherry
Vim(1-138) expression
Image 15.imx

## Slide 41
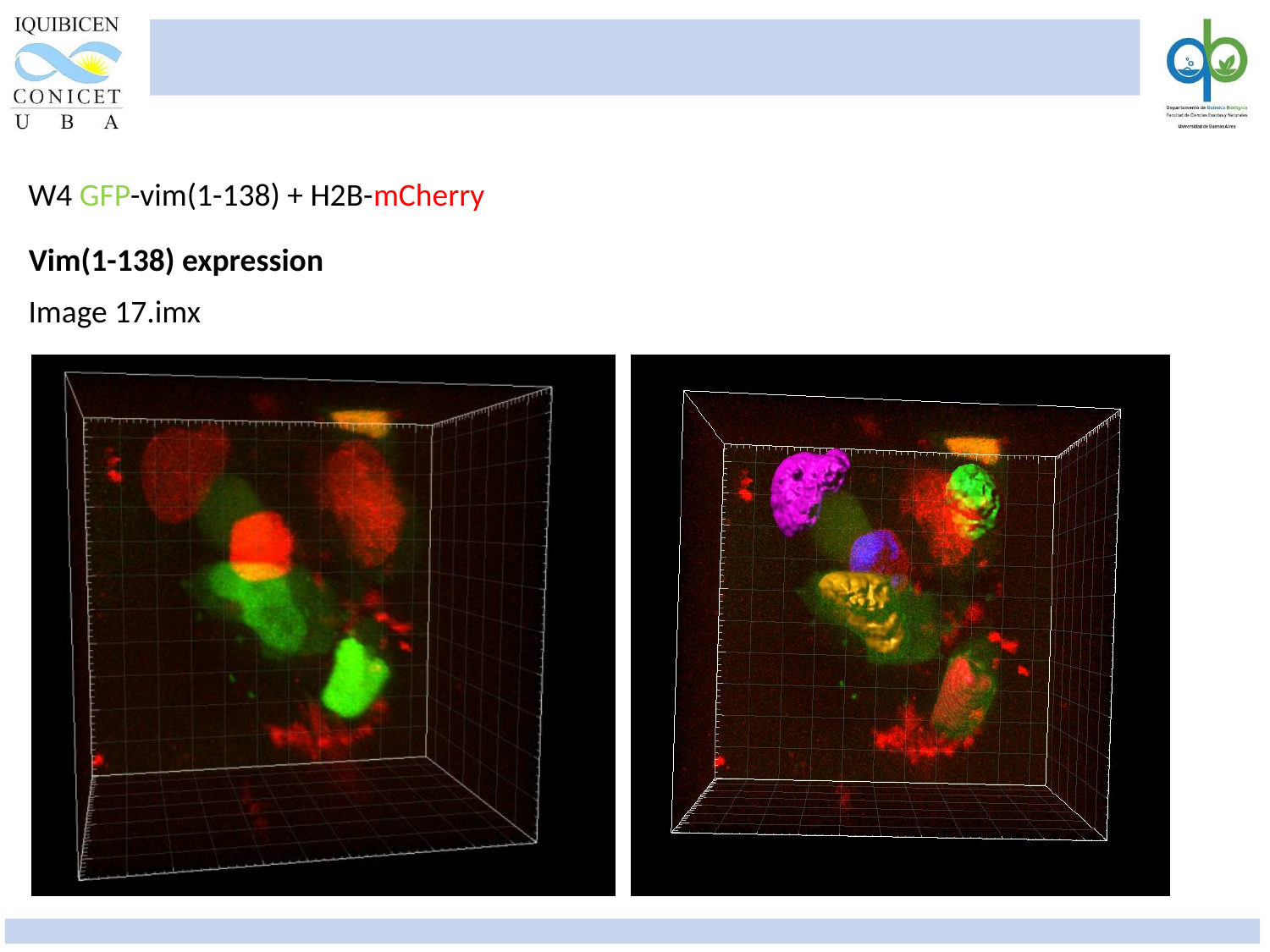

W4 GFP-vim(1-138) + H2B-mCherry
Vim(1-138) expression
Image 17.imx

## Slide 42
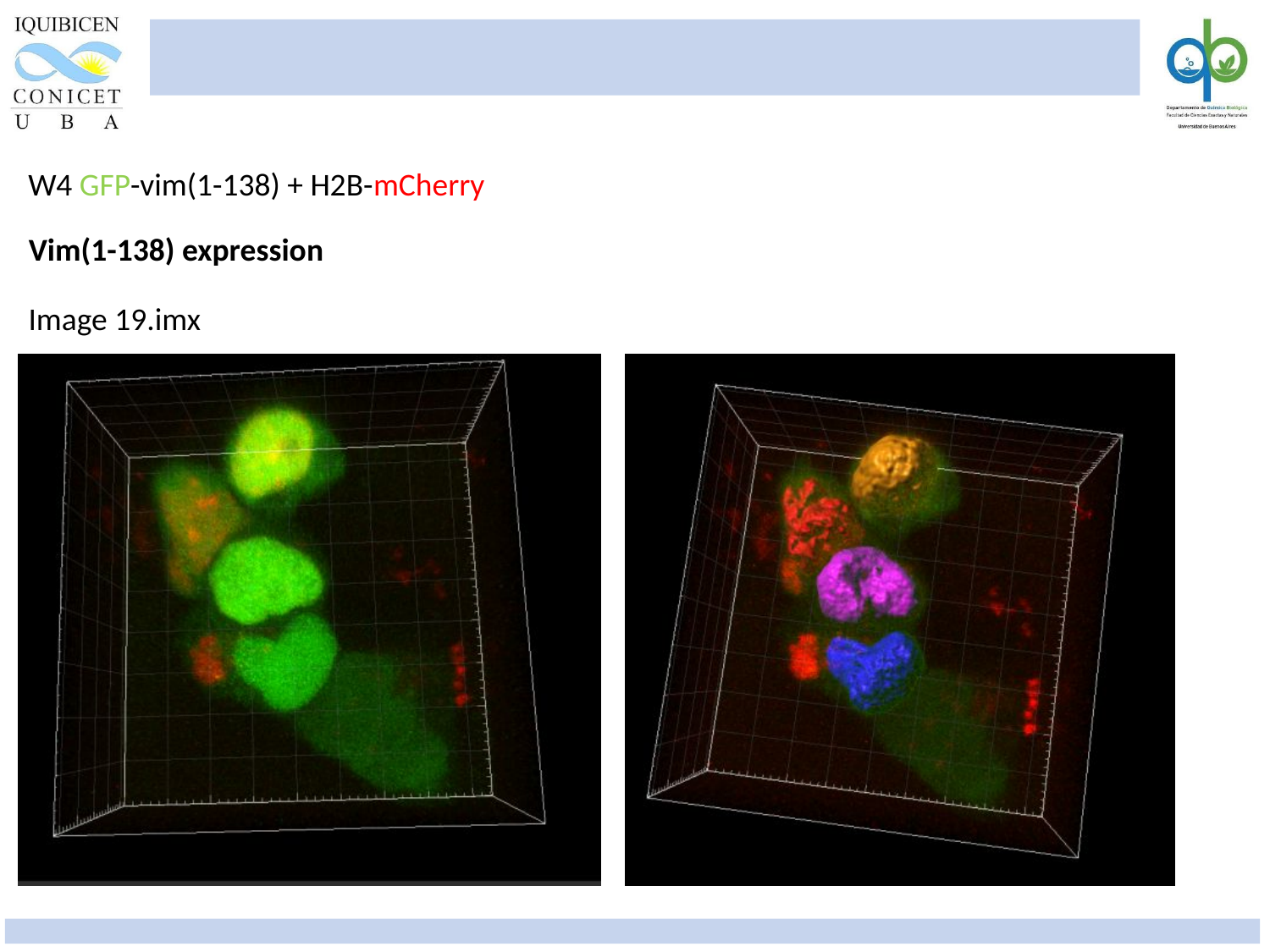

W4 GFP-vim(1-138) + H2B-mCherry
Vim(1-138) expression
Image 19.imx

## Slide 43
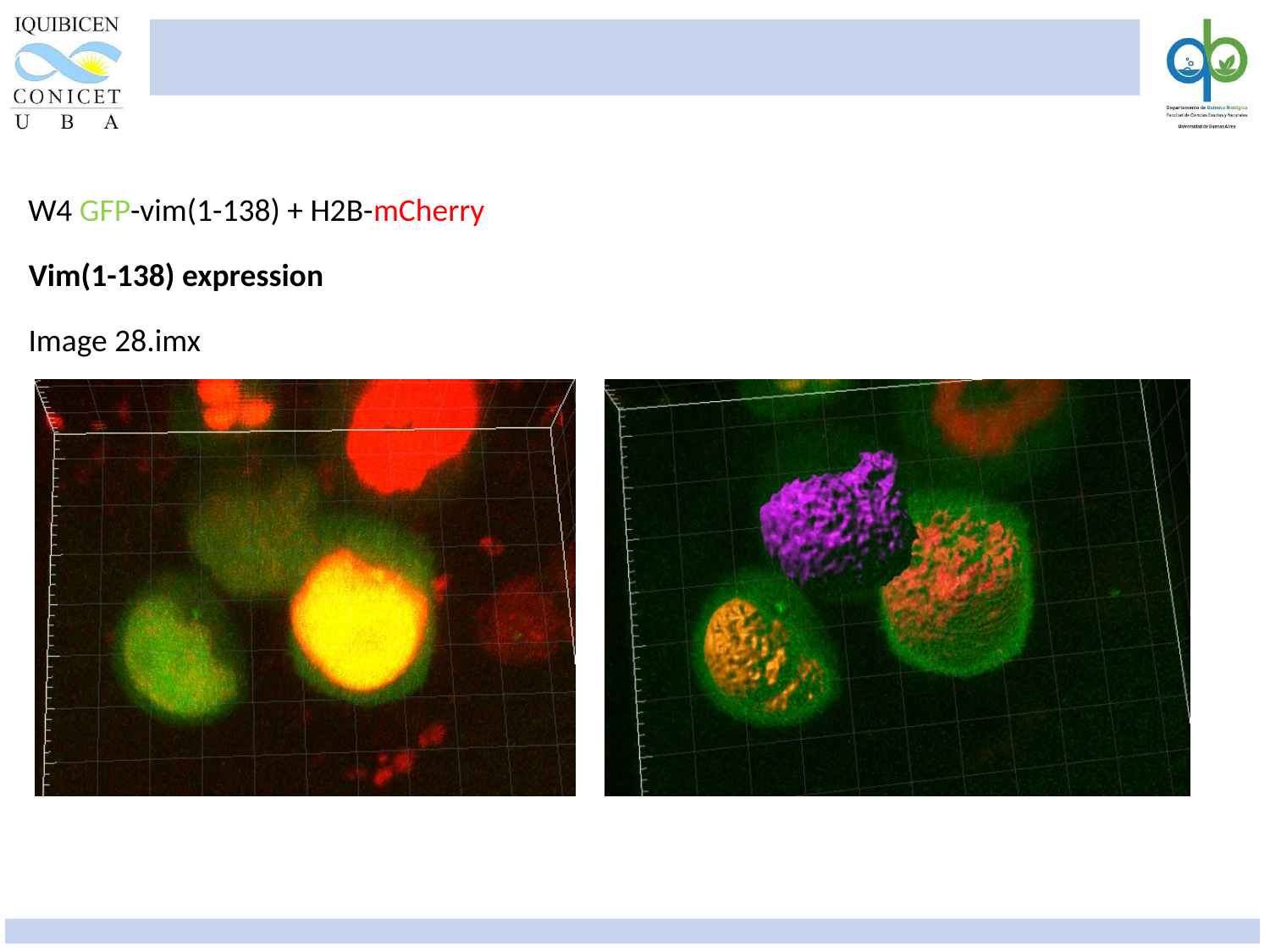

W4 GFP-vim(1-138) + H2B-mCherry
Vim(1-138) expression
Image 28.imx

## Slide 44
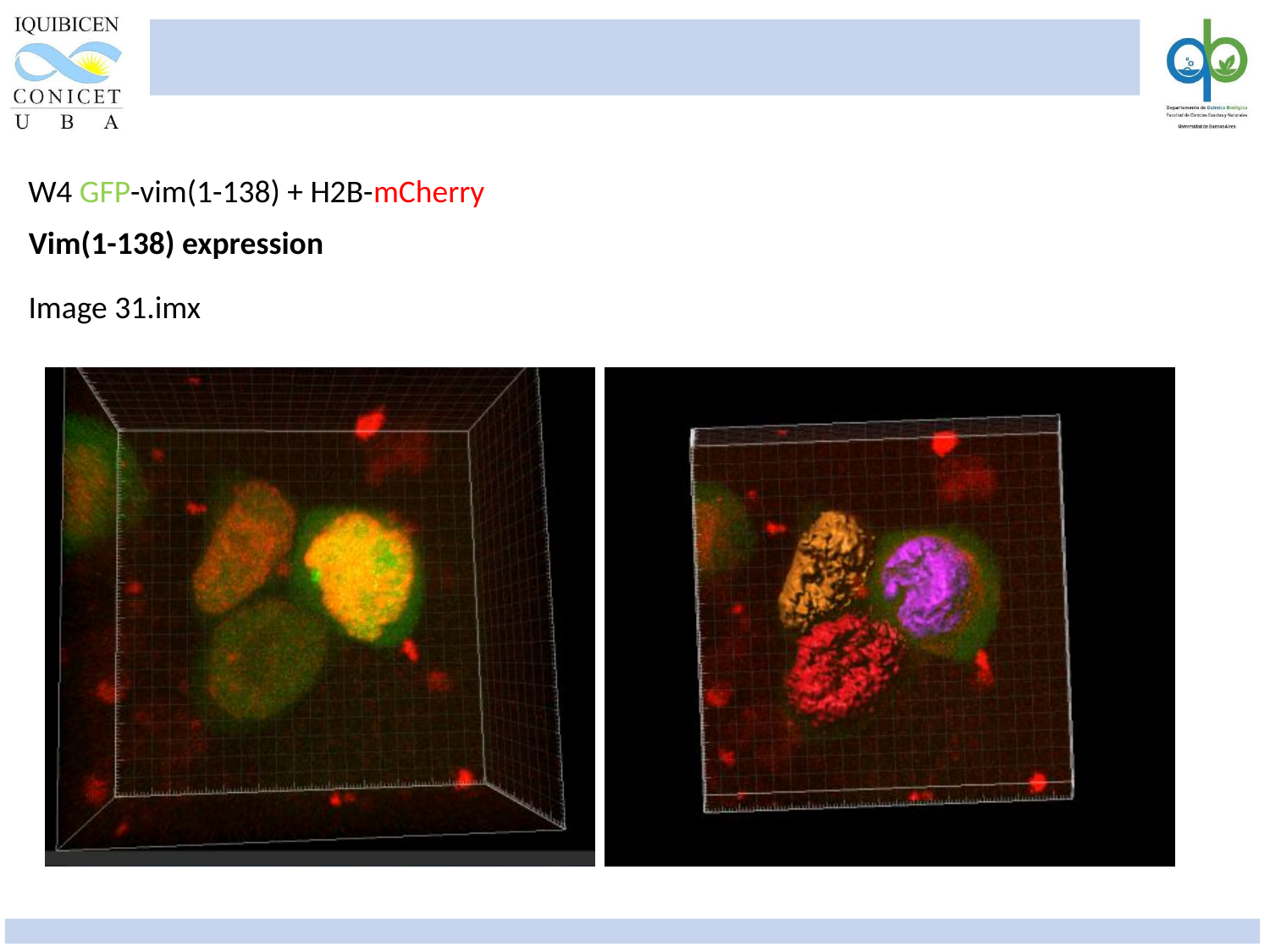

W4 GFP-vim(1-138) + H2B-mCherry
Vim(1-138) expression
Image 31.imx

## Slide 45
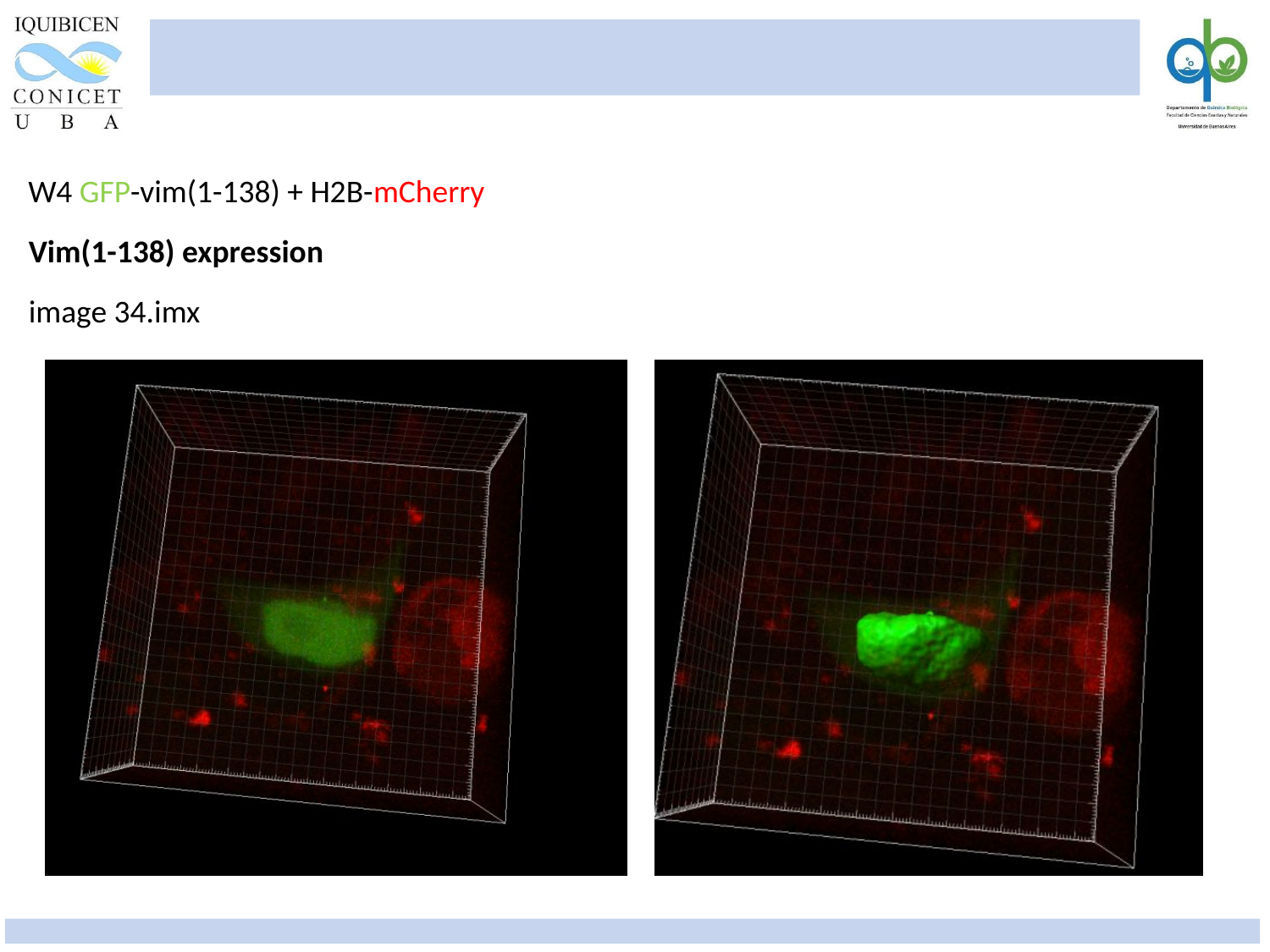

W4 GFP-vim(1-138) + H2B-mCherry
Vim(1-138) expression
image 34.imx

## Slide 46
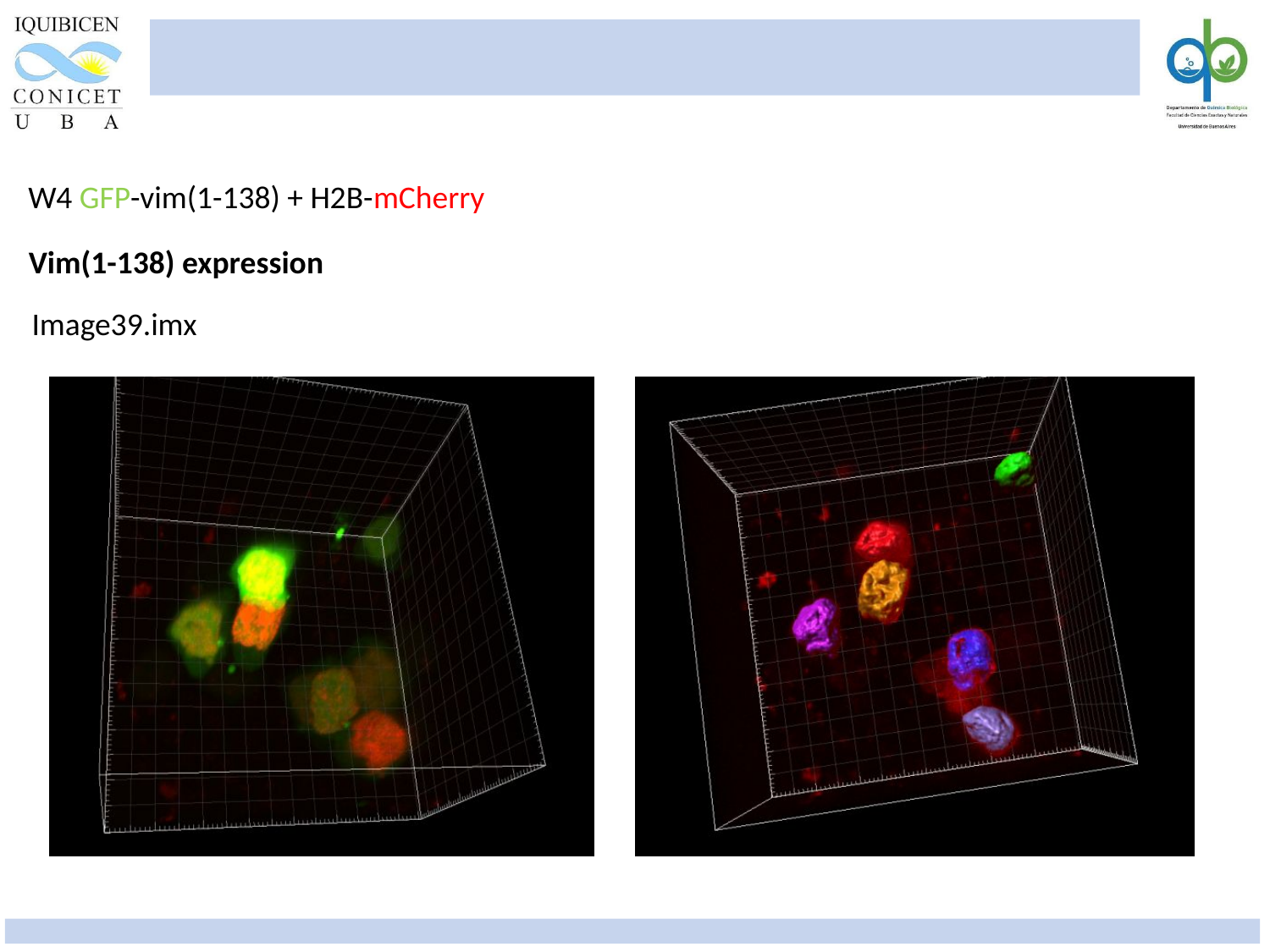

W4 GFP-vim(1-138) + H2B-mCherry
Vim(1-138) expression
Image39.imx

## Slide 47
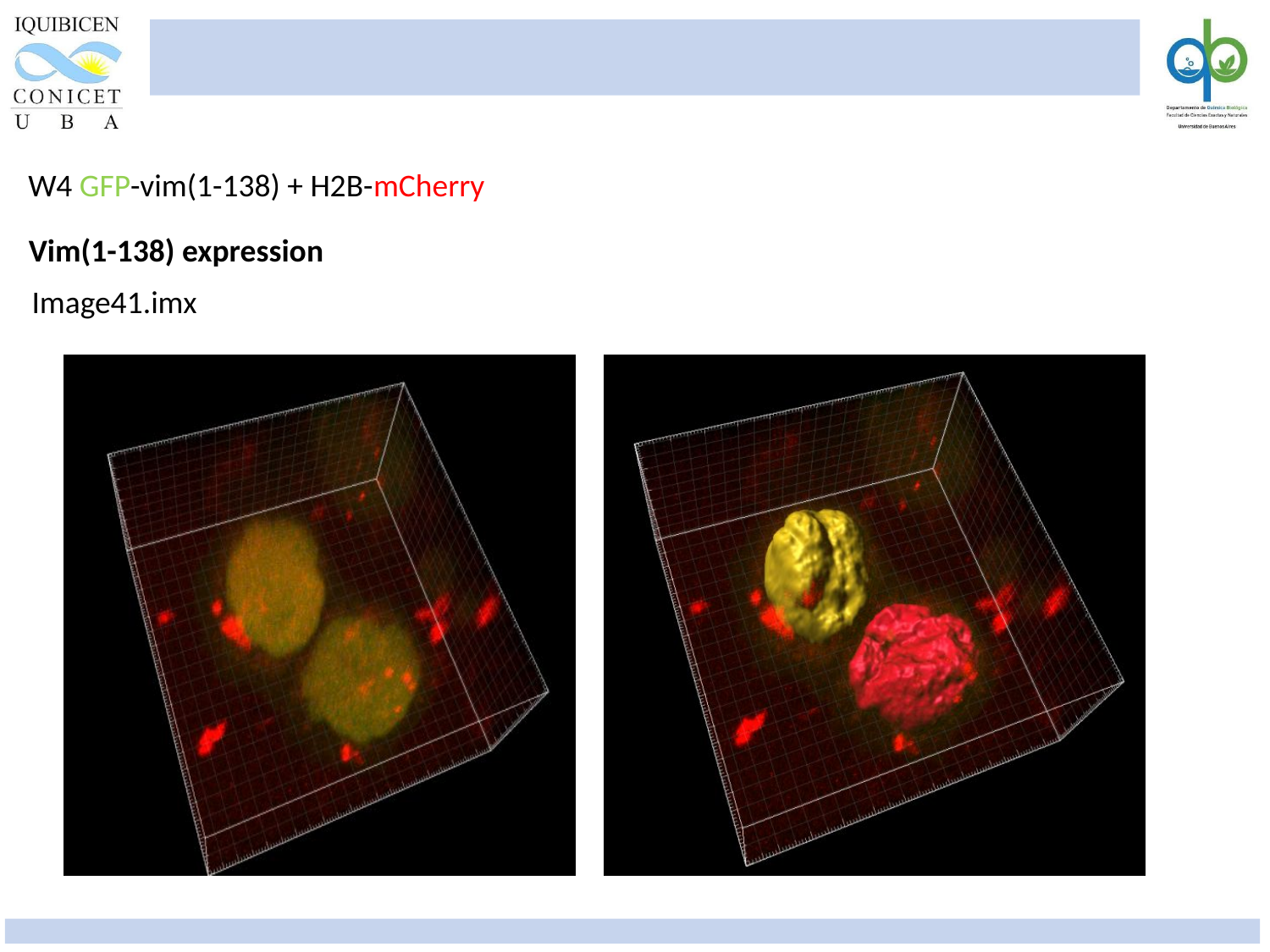

W4 GFP-vim(1-138) + H2B-mCherry
Vim(1-138) expression
Image41.imx

## Slide 48
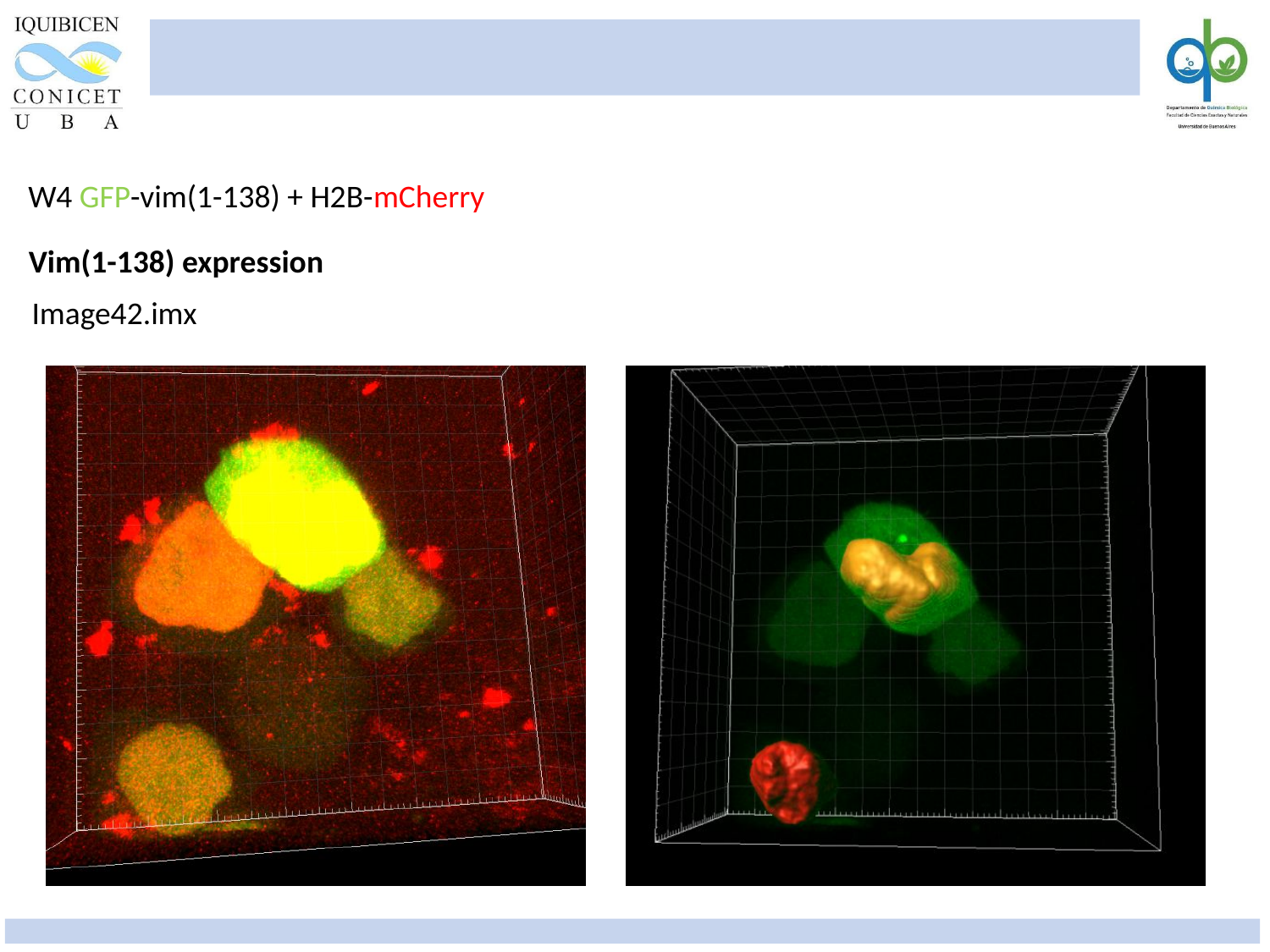

W4 GFP-vim(1-138) + H2B-mCherry
Vim(1-138) expression
Image42.imx

## Slide 49
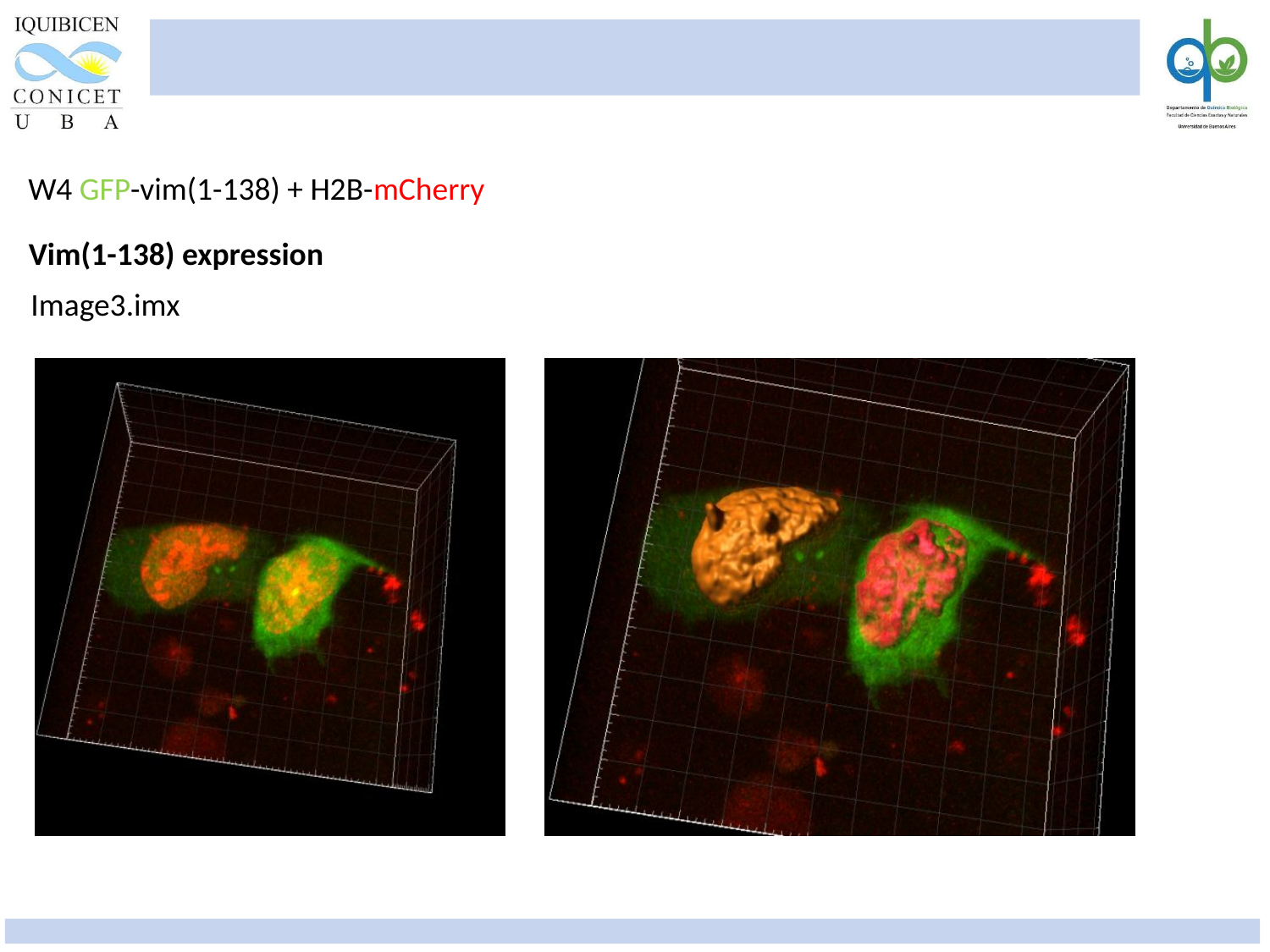

W4 GFP-vim(1-138) + H2B-mCherry
Vim(1-138) expression
Image3.imx

## Slide 50
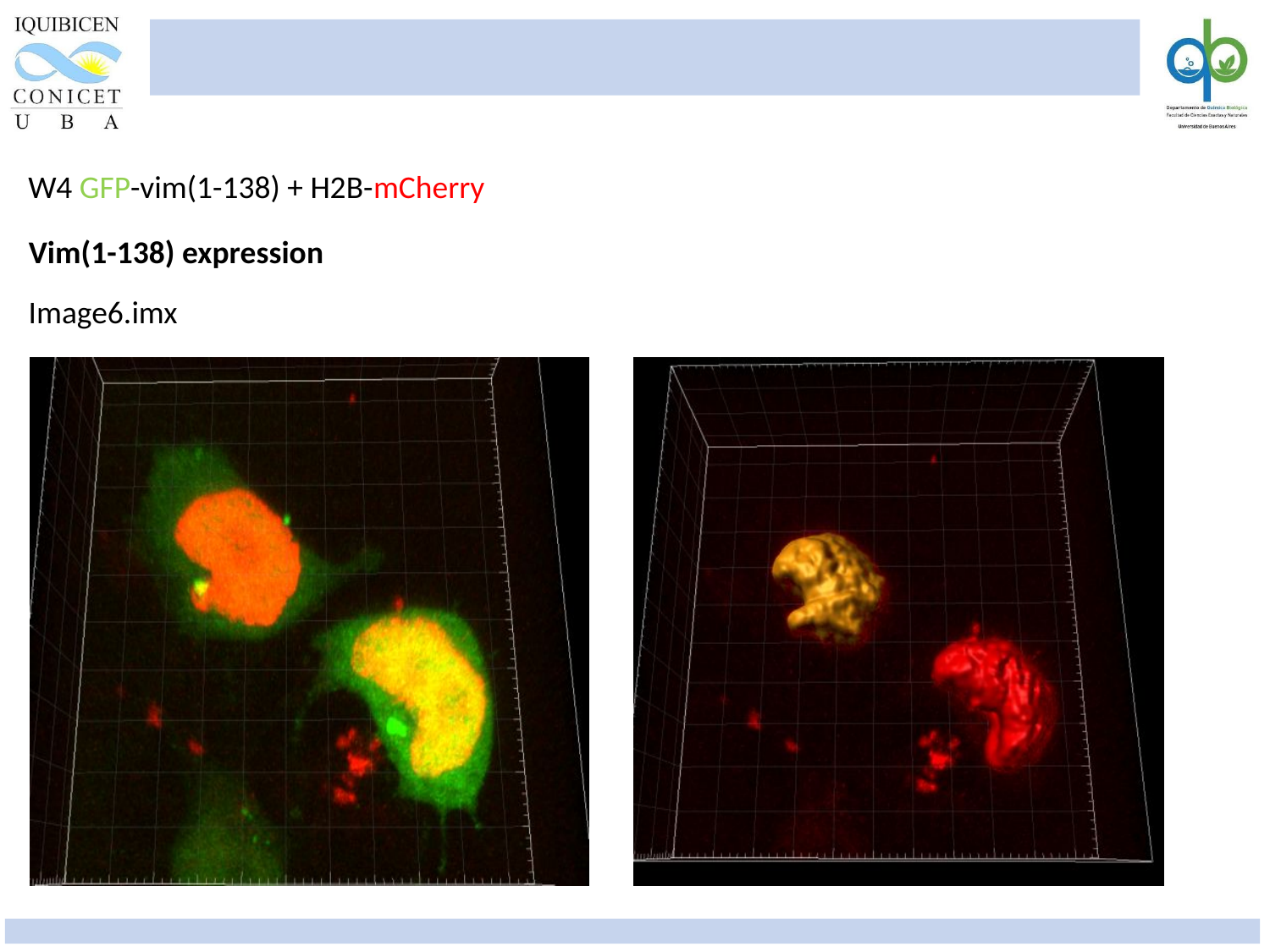

W4 GFP-vim(1-138) + H2B-mCherry
Vim(1-138) expression
Image6.imx

## Slide 51
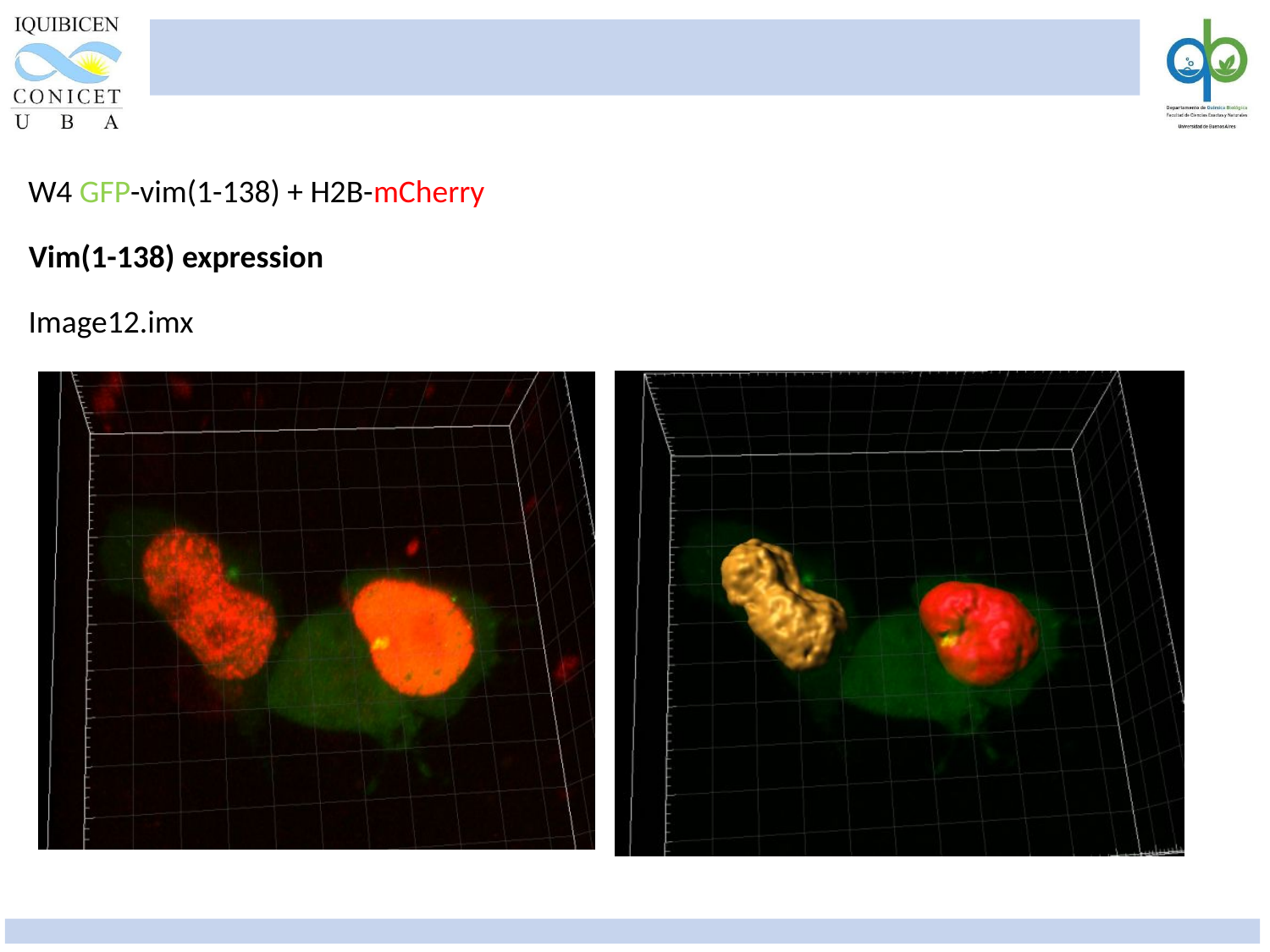

W4 GFP-vim(1-138) + H2B-mCherry
Vim(1-138) expression
Image12.imx

## Slide 52
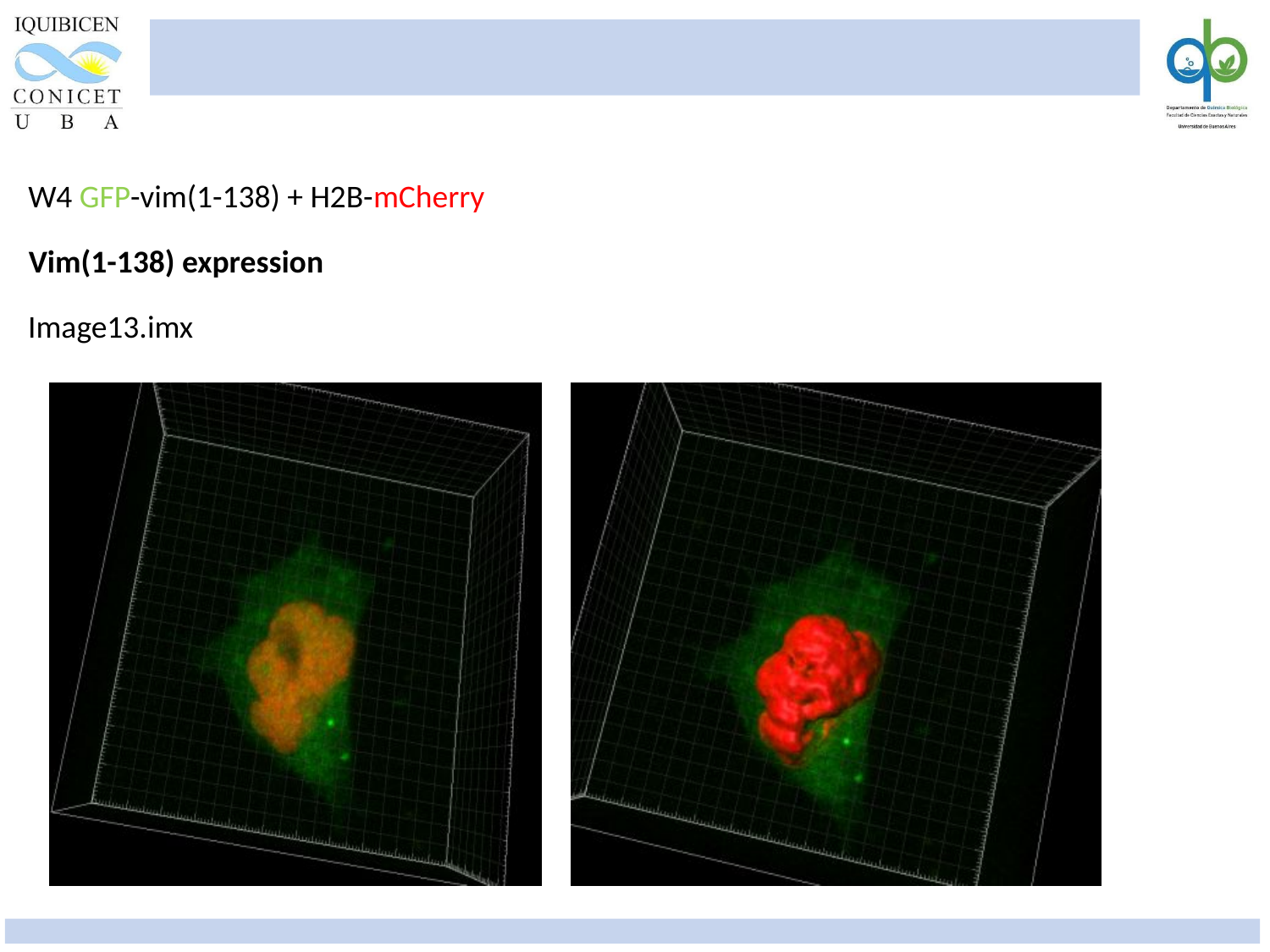

W4 GFP-vim(1-138) + H2B-mCherry
Vim(1-138) expression
Image13.imx
