# Supplementary material for: Nucleus-cytoskeleton communication impacts on OCT4-chromatin interactions in embryonic stem cells
Source: BMC Biol. 2022 Jan 7;20:6. doi: 10.1186/s12915-021-01207-w (PMC8742348; doi:10.1186/s12915-021-01207-w)

## Slide 1
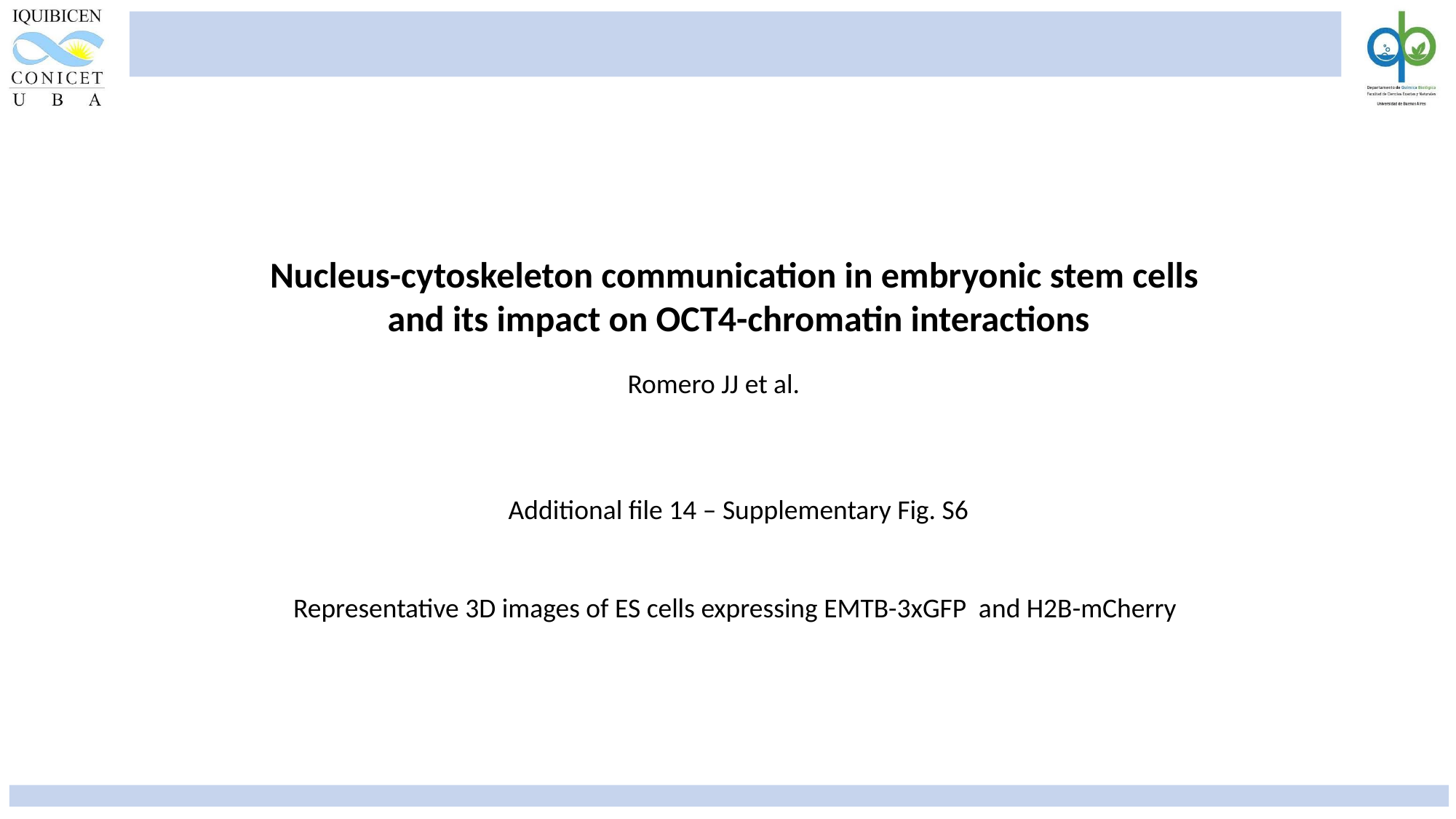

Nucleus-cytoskeleton communication in embryonic stem cells
and its impact on OCT4-chromatin interactions
Romero JJ et al.
Additional file 14 – Supplementary Fig. S6
Representative 3D images of ES cells expressing EMTB-3xGFP and H2B-mCherry

## Slide 2
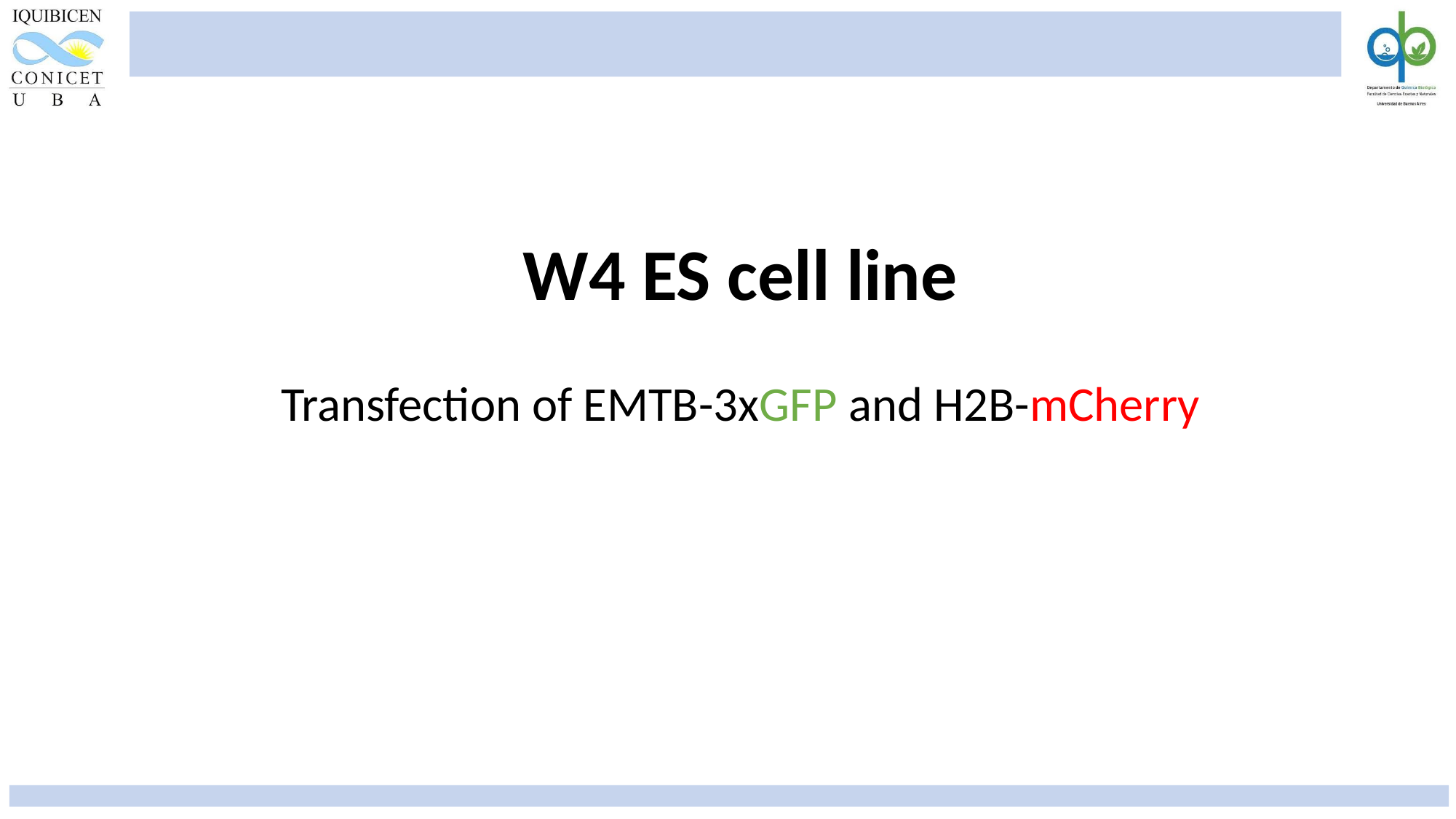

W4 ES cell line
Transfection of EMTB-3xGFP and H2B-mCherry

## Slide 3
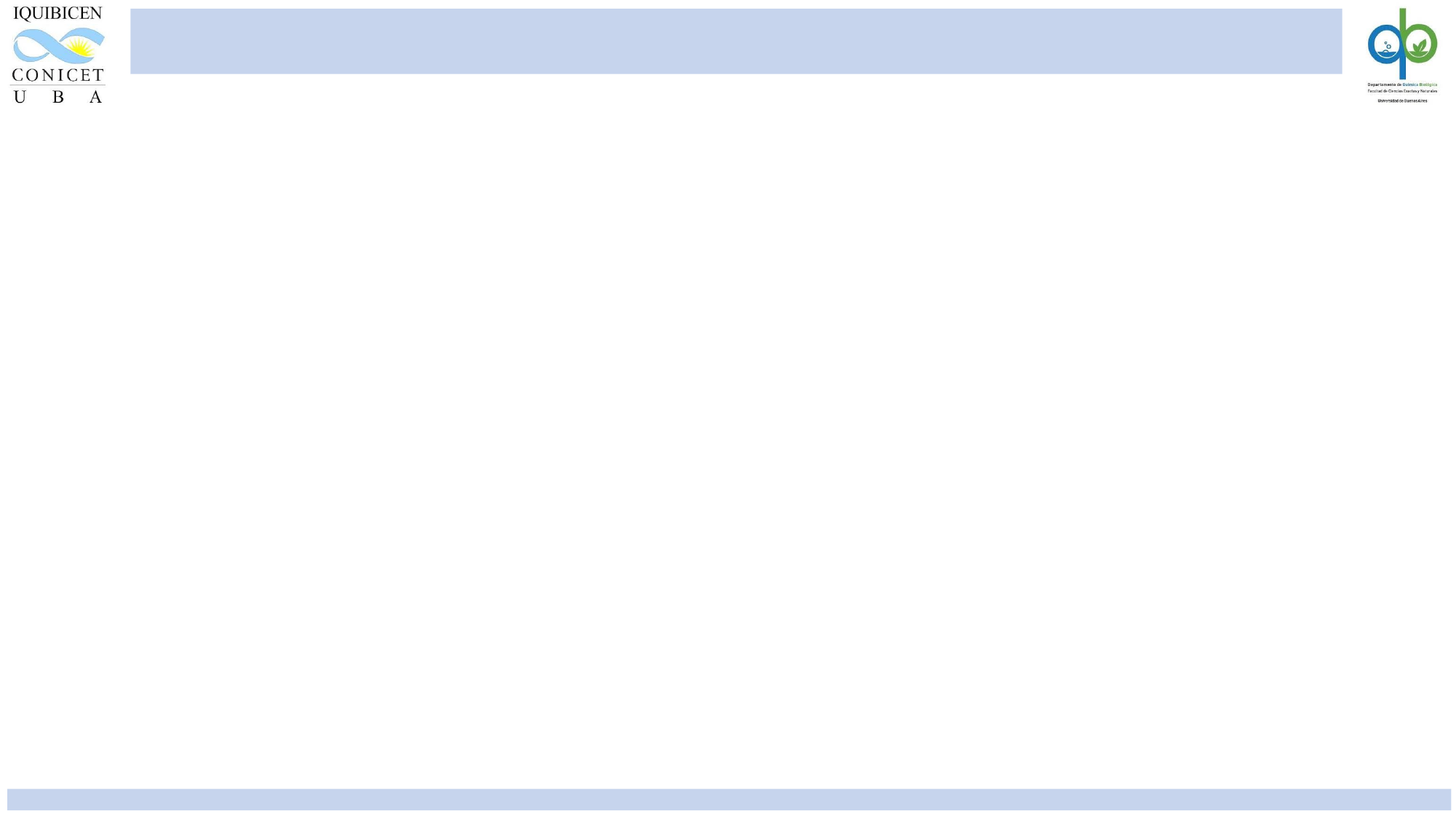

## Slide 4
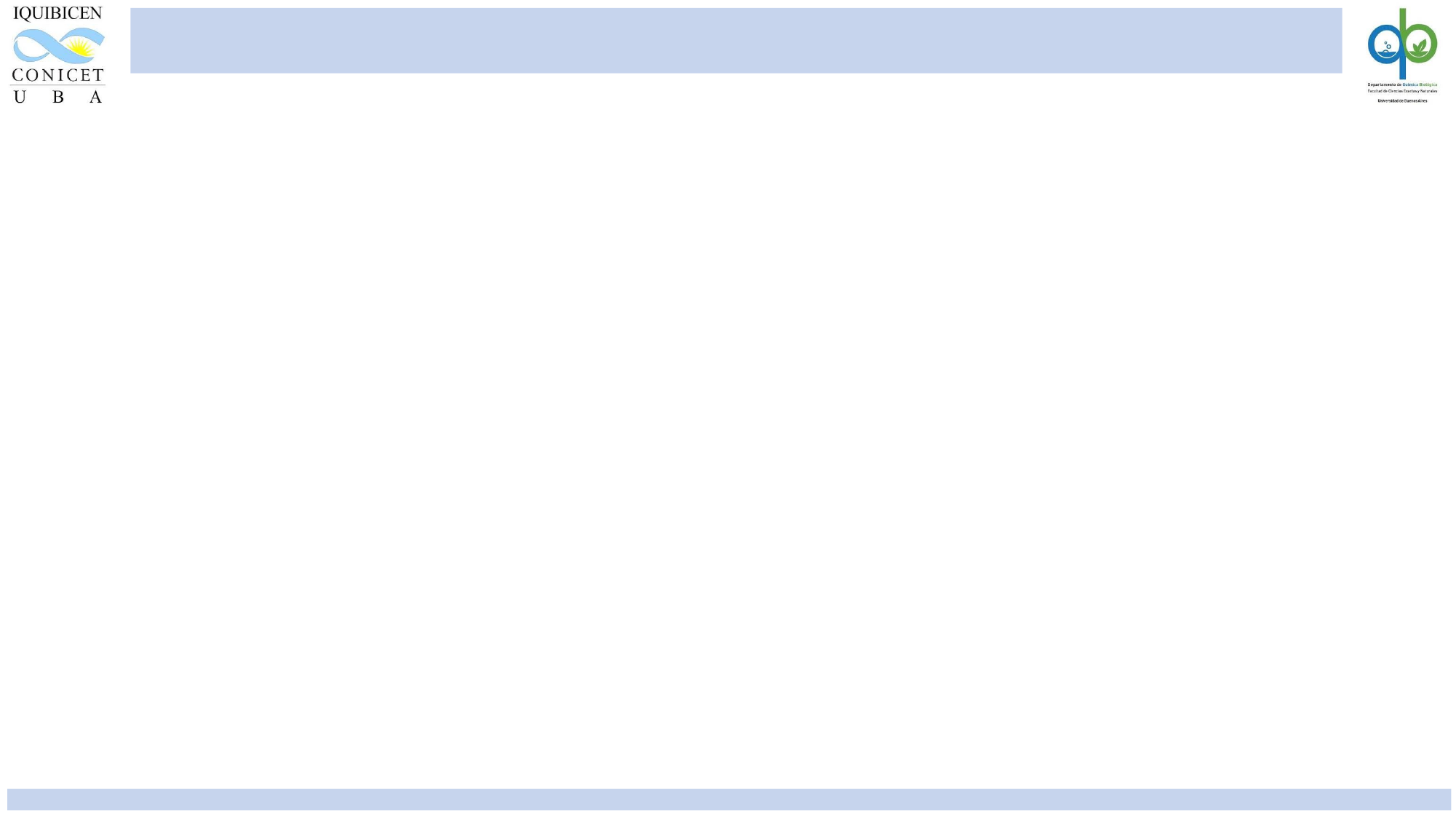

## Slide 5
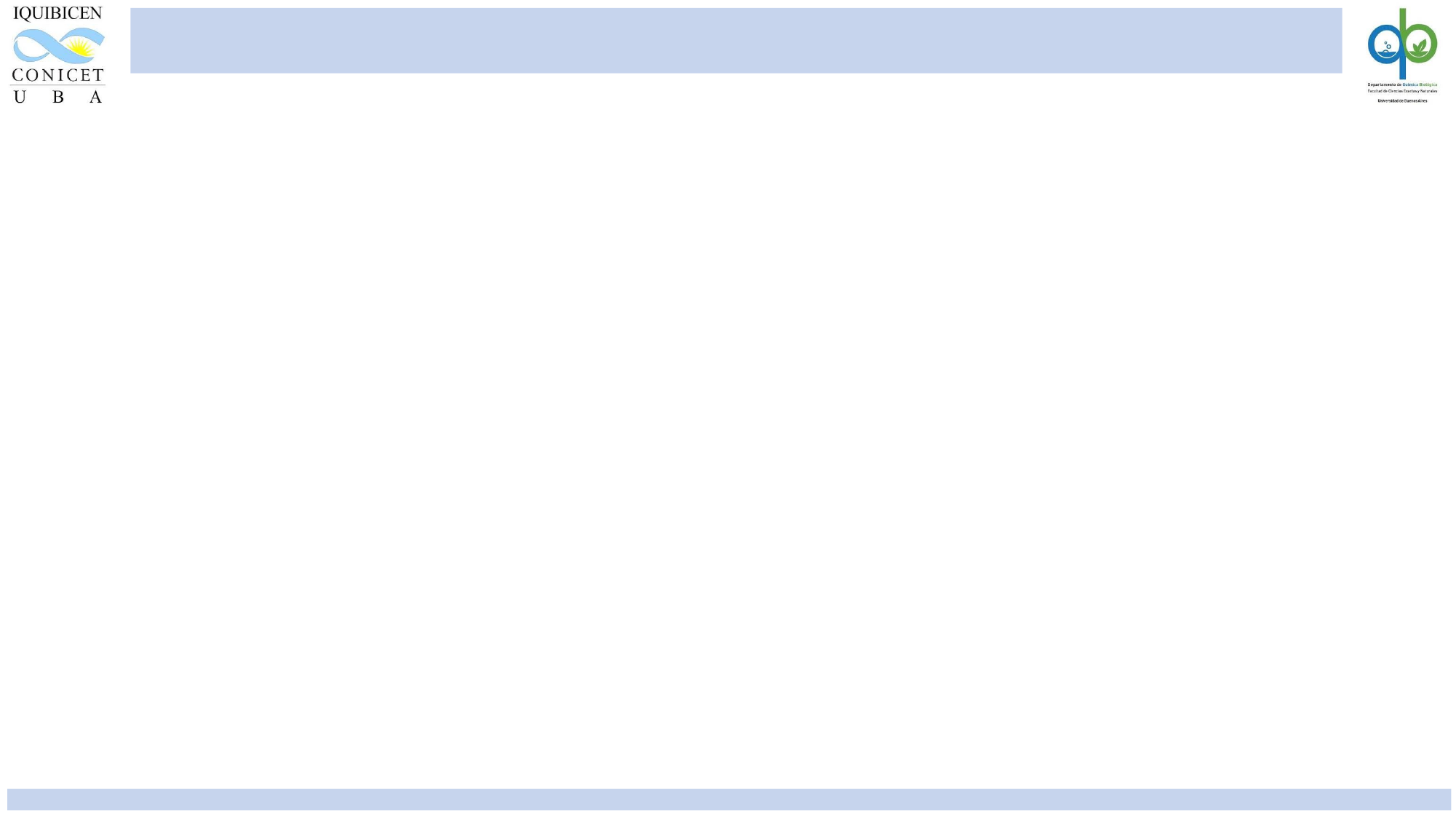

## Slide 6
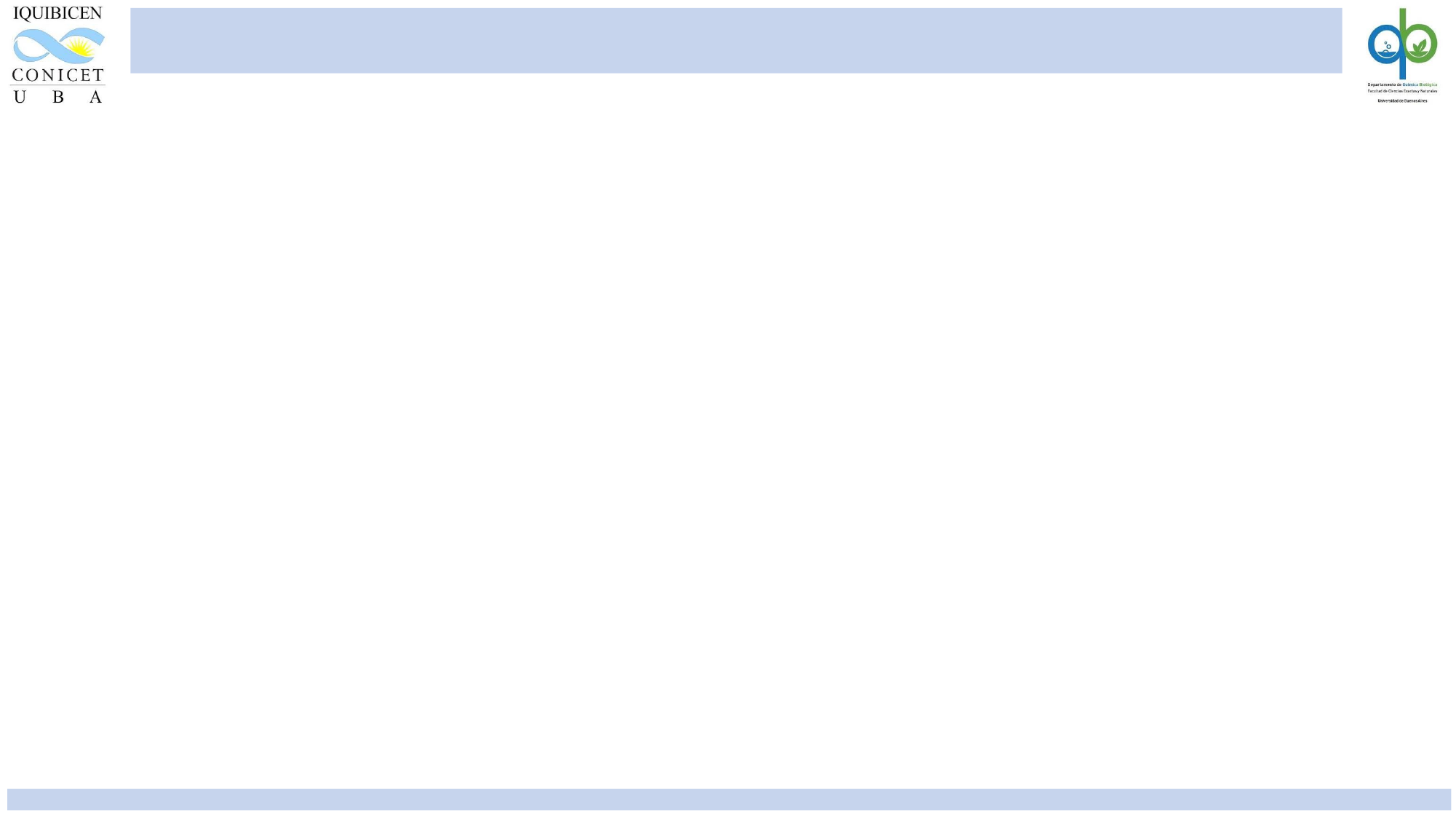

## Slide 7
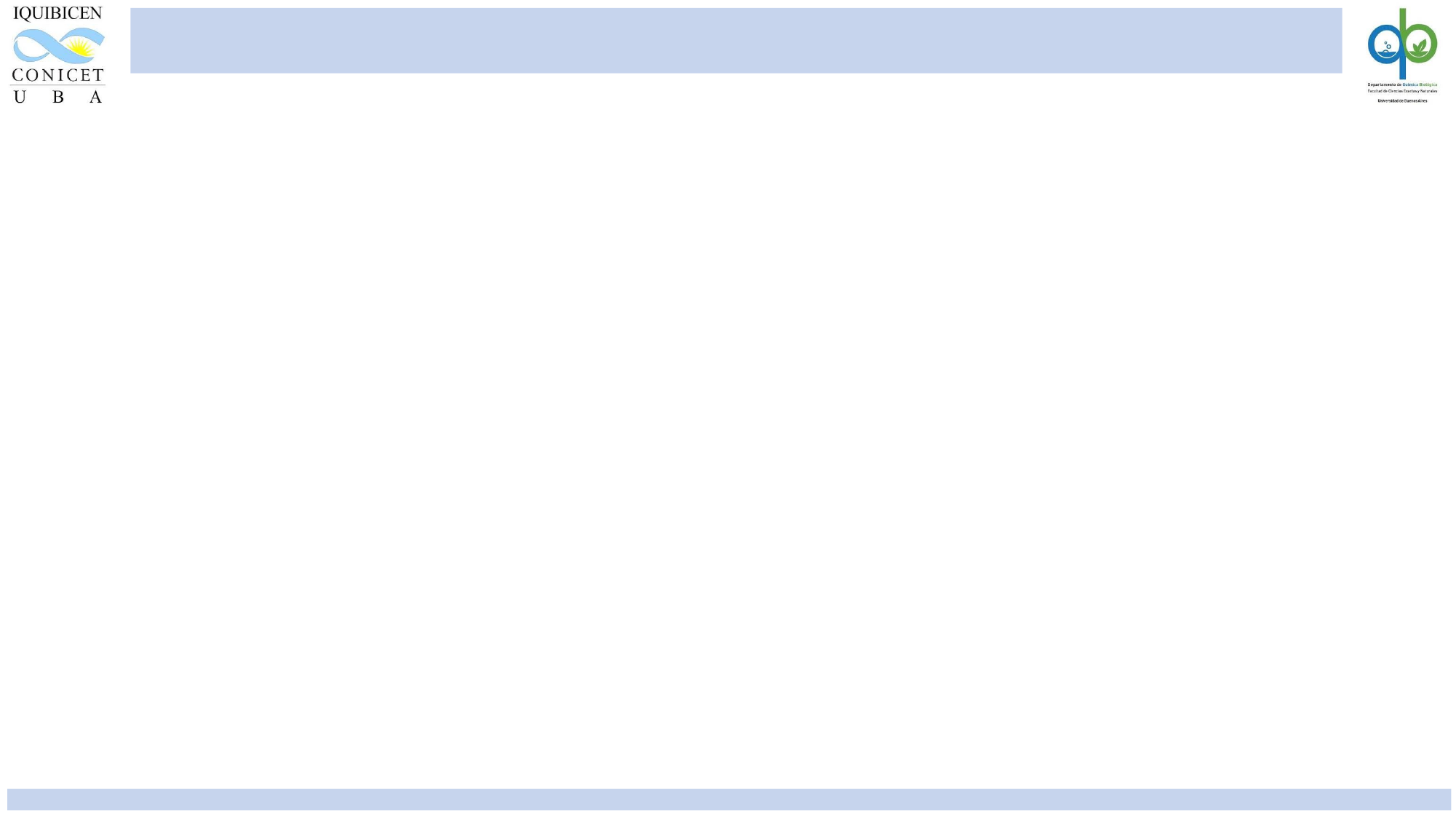

## Slide 8
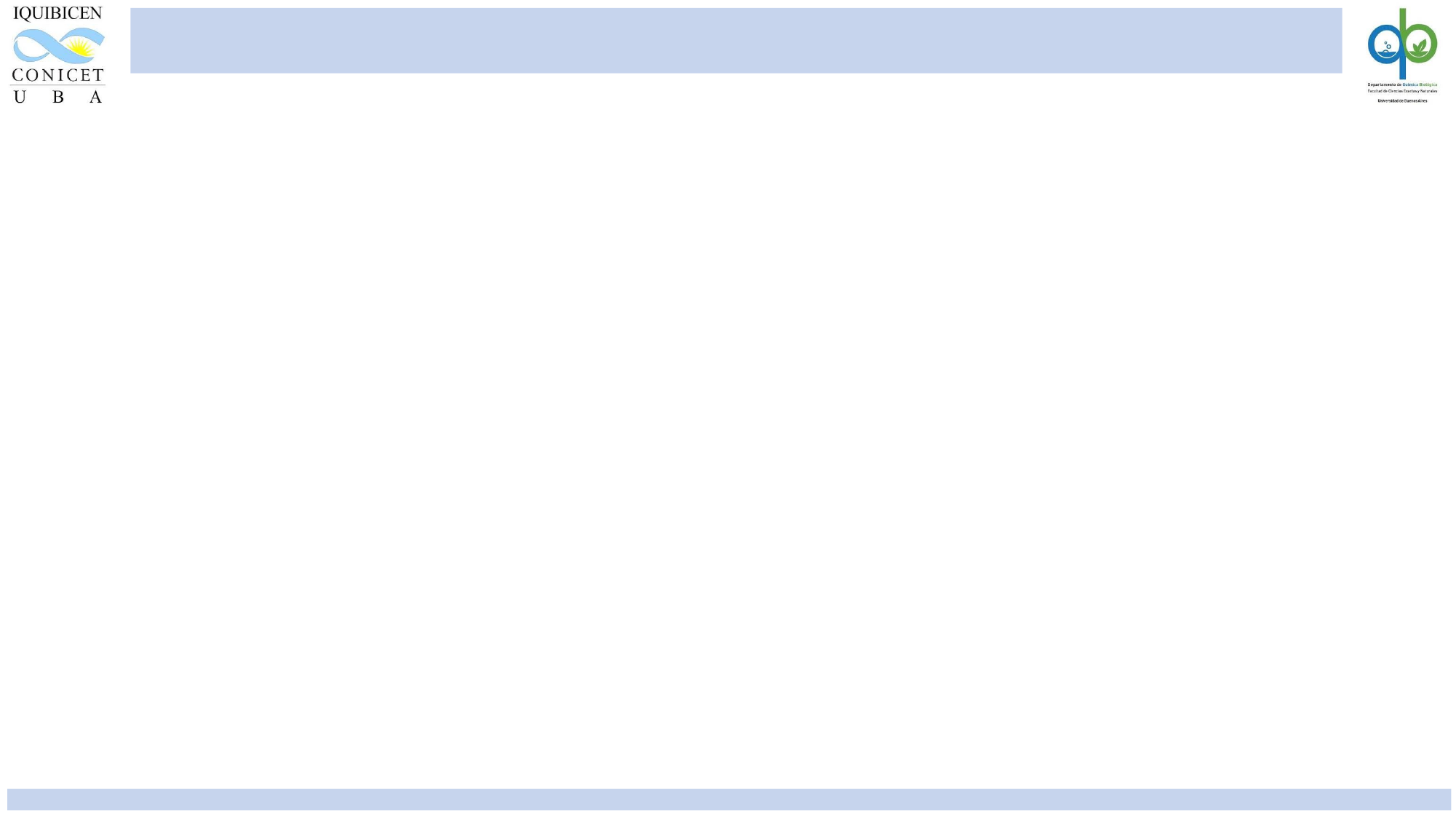

## Slide 9
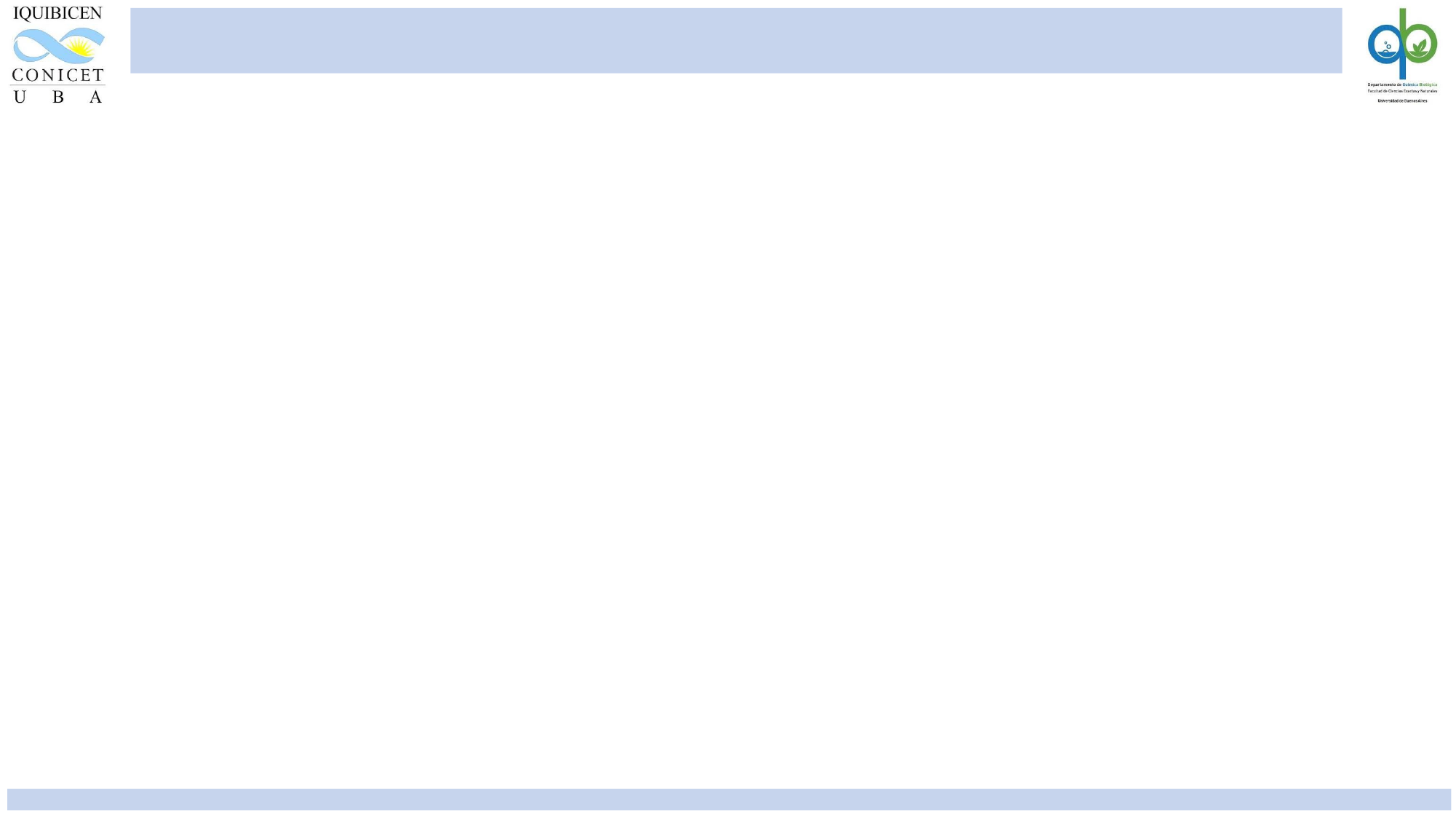

## Slide 10
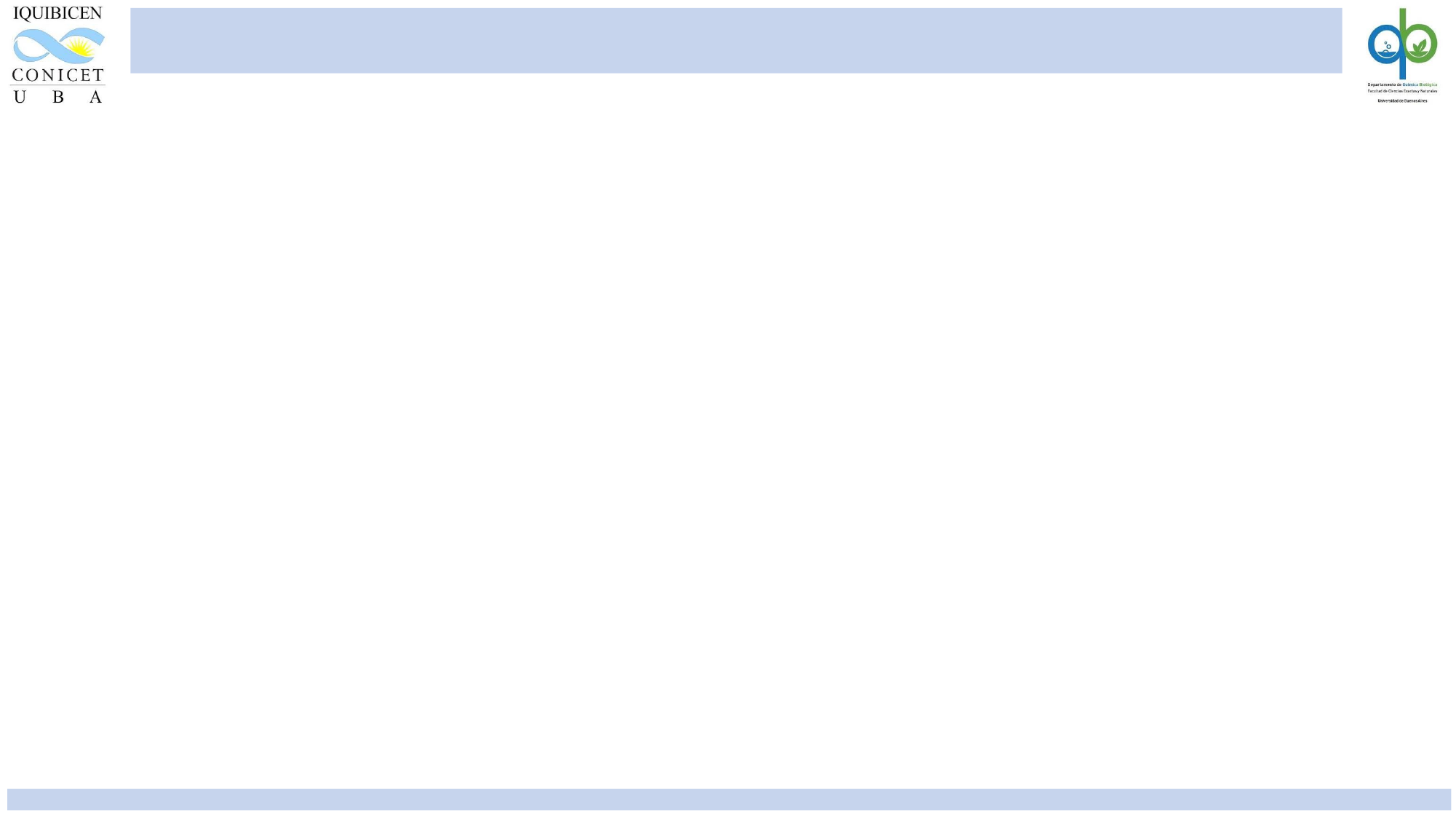

## Slide 11
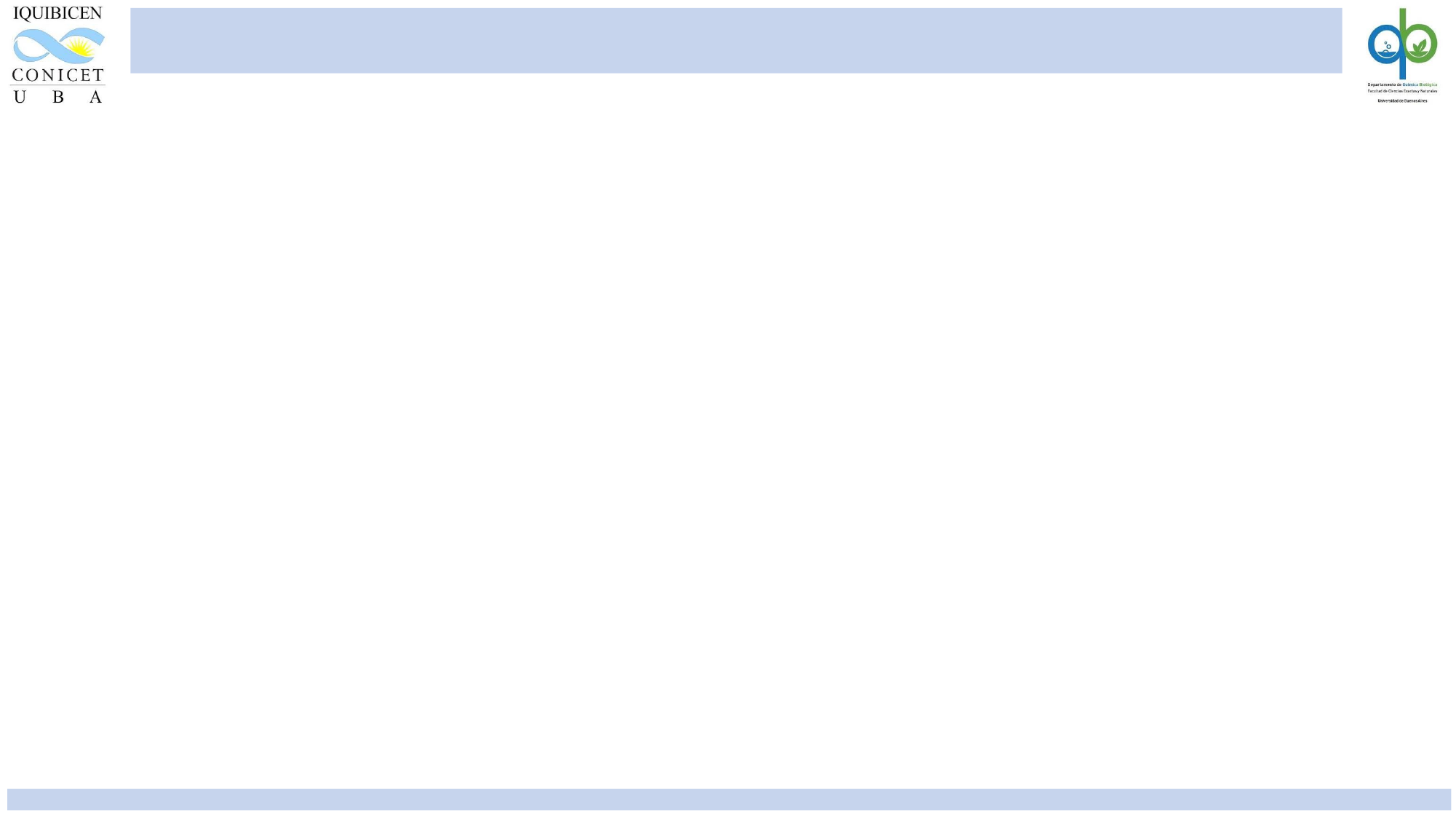

## Slide 12
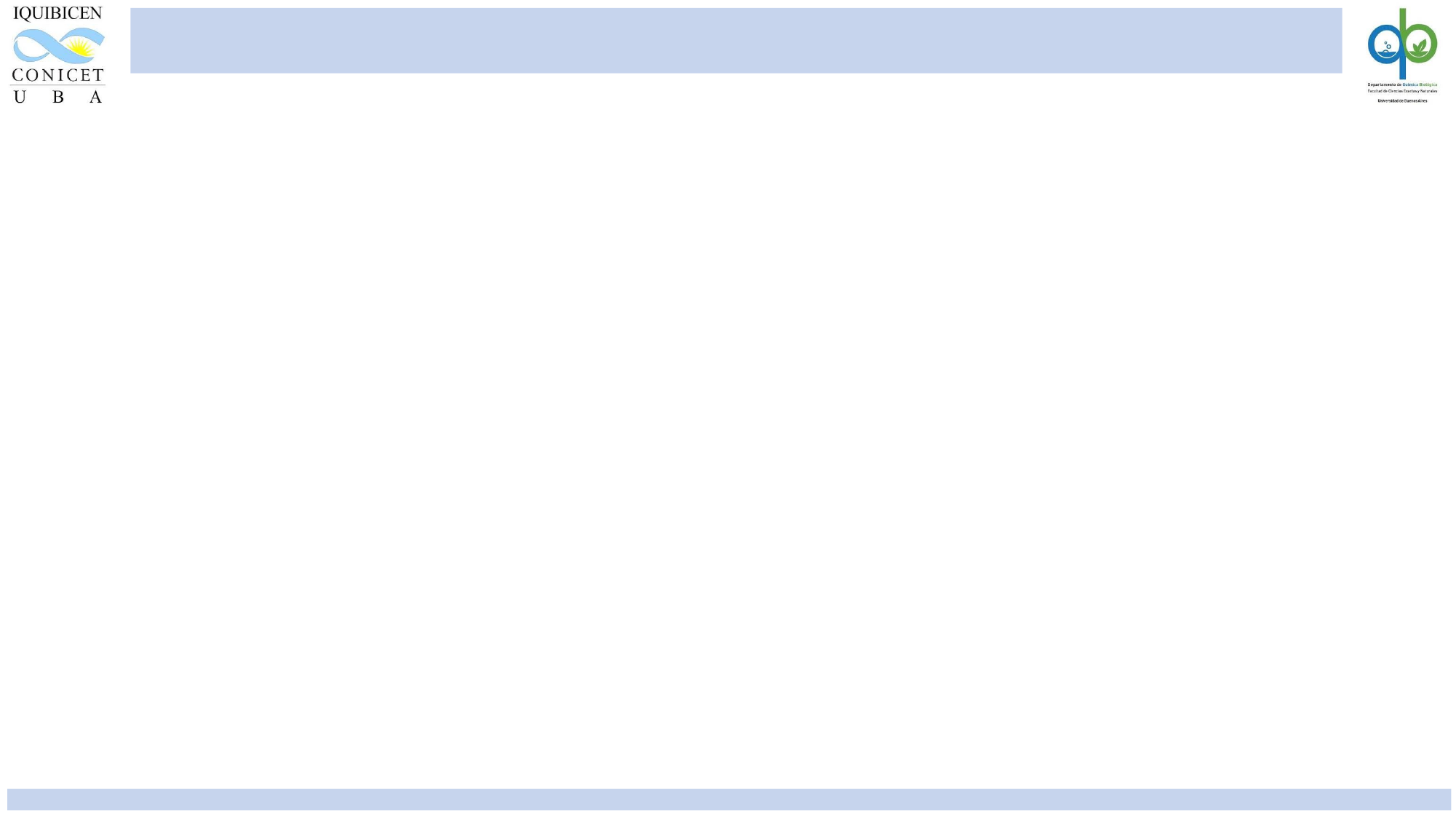

Supplement: Supplementary file 6 — Additional file 6. Supplementary Video S5. EB3-GFP comets in close contact with the cell nucleus. ES cells transfected with EB3-GFP and H2B-mCherry were imaged at 0.6 frames/s (100 frames). Related to Fig. 1c. [file 12915_2021_1207_MOESM6_ESM.pptx]
