# Supplementary material for: Nucleus-cytoskeleton communication impacts on OCT4-chromatin interactions in embryonic stem cells
Source: BMC Biol. 2022 Jan 7;20:6. doi: 10.1186/s12915-021-01207-w (PMC8742348; doi:10.1186/s12915-021-01207-w)
Supplement: Supplementary file 7 — Additional file 7. Supplementary Table S1. Meta-analysis of microarray, RNA-seq and proteomic datasets analyzed in this work. [file 12915_2021_1207_MOESM7_ESM.pptx]

## Slide 1
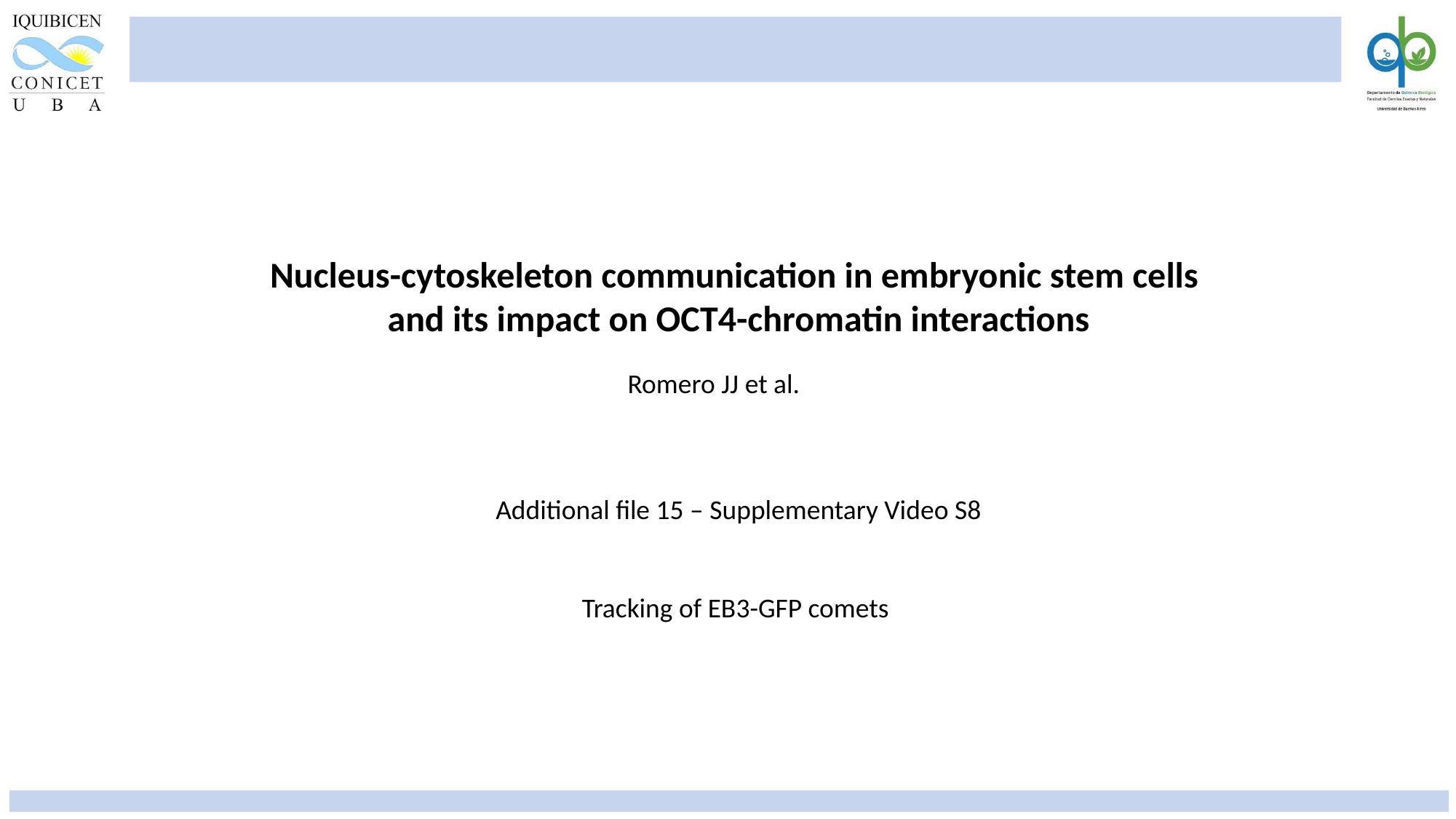

Nucleus-cytoskeleton communication in embryonic stem cells
and its impact on OCT4-chromatin interactions
Romero JJ et al.
Additional file 15 – Supplementary Video S8
Tracking of EB3-GFP comets

## Slide 2
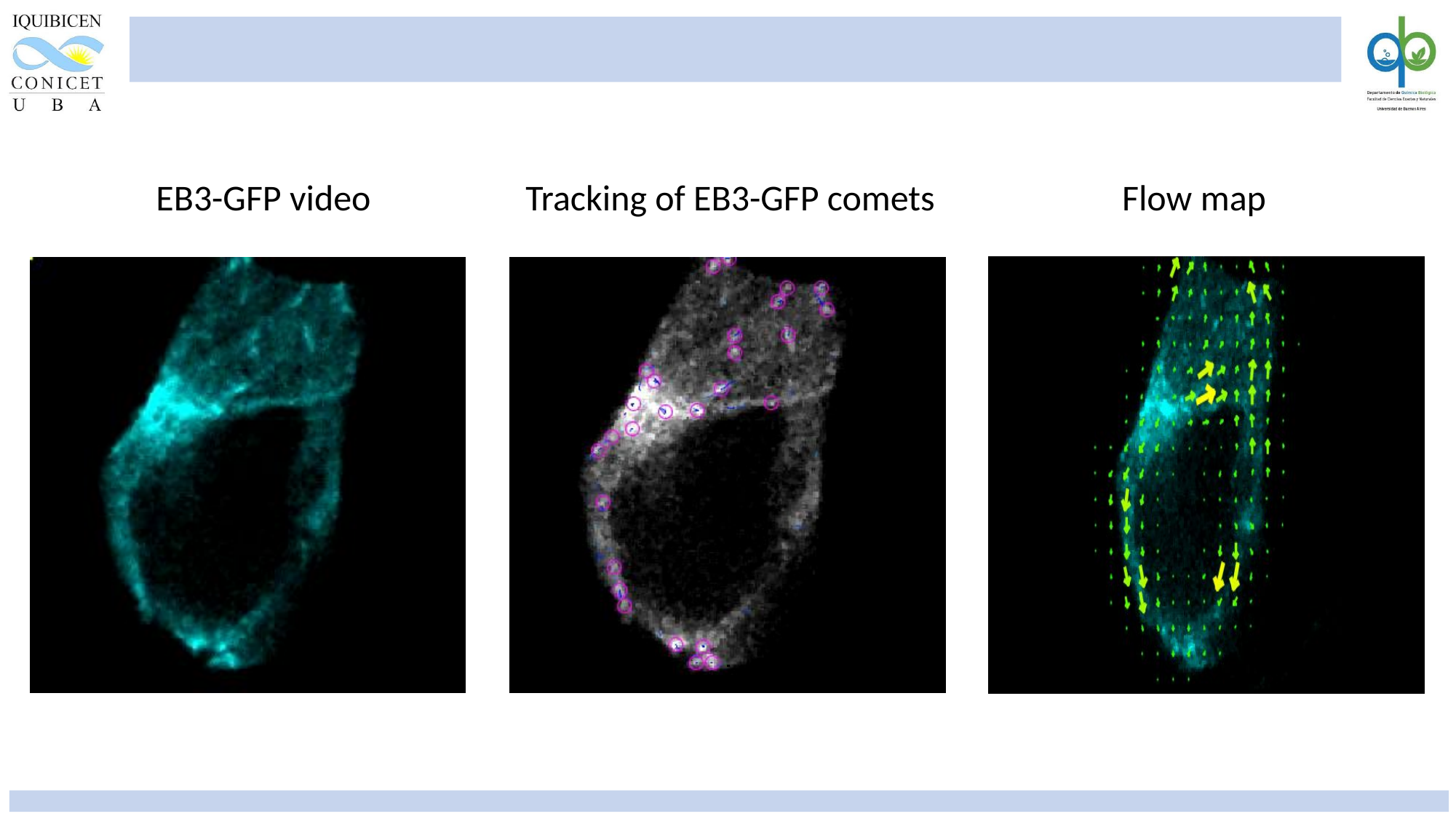

EB3-GFP video
Tracking of EB3-GFP comets
Flow map

## Slide 3
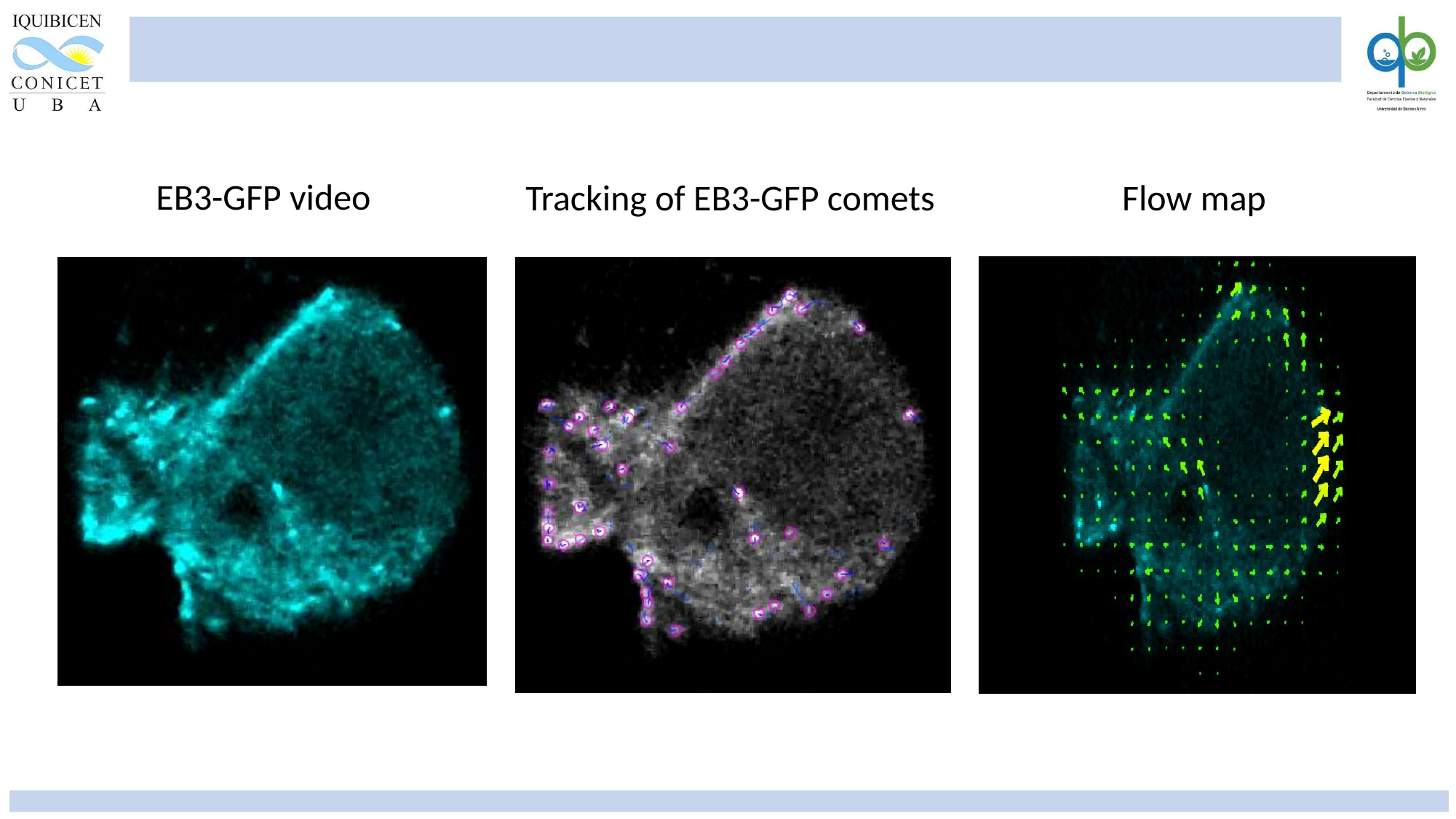

EB3-GFP video
Tracking of EB3-GFP comets
Flow map

## Slide 4
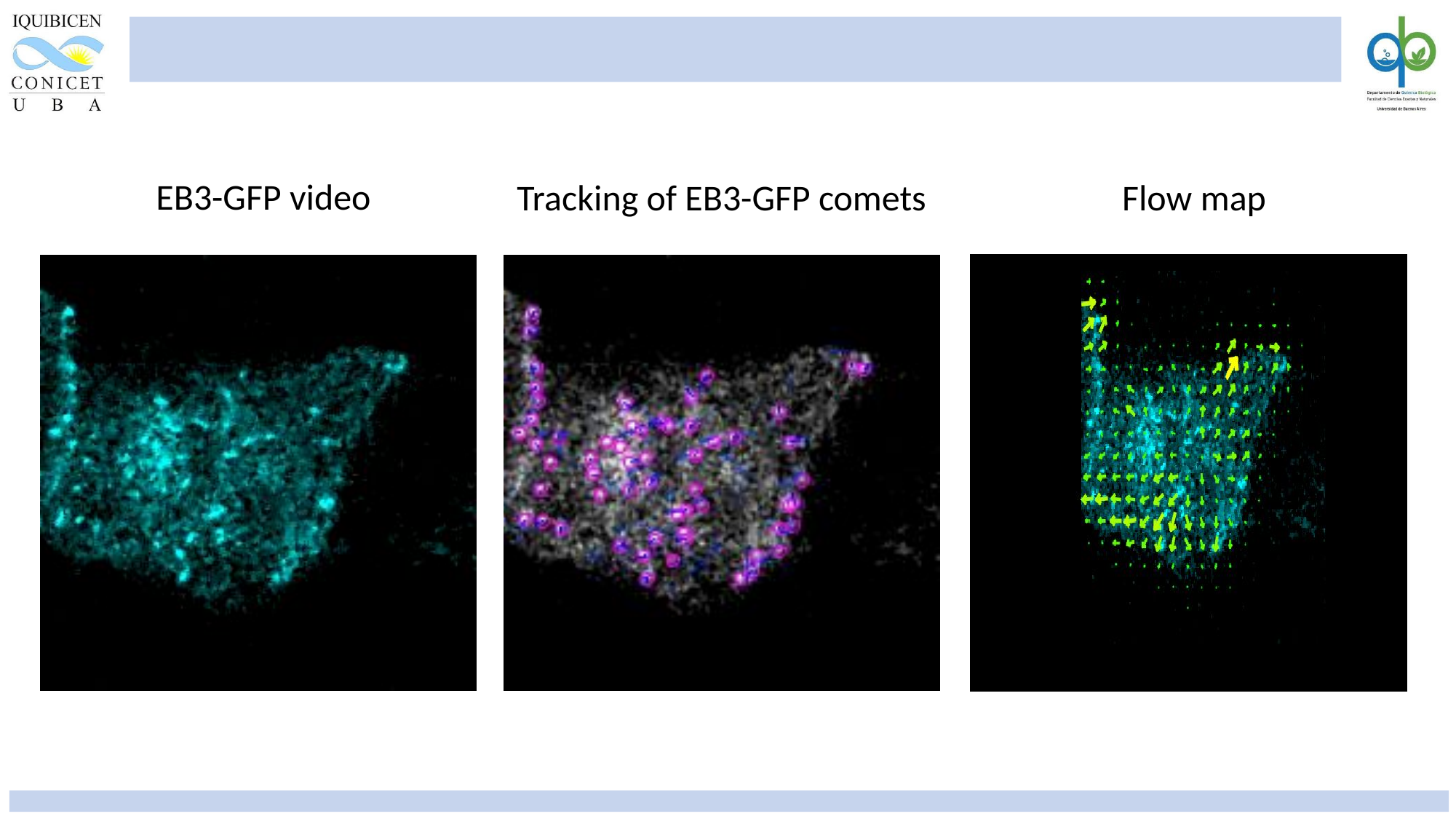

EB3-GFP video
Tracking of EB3-GFP comets
Flow map

## Slide 5
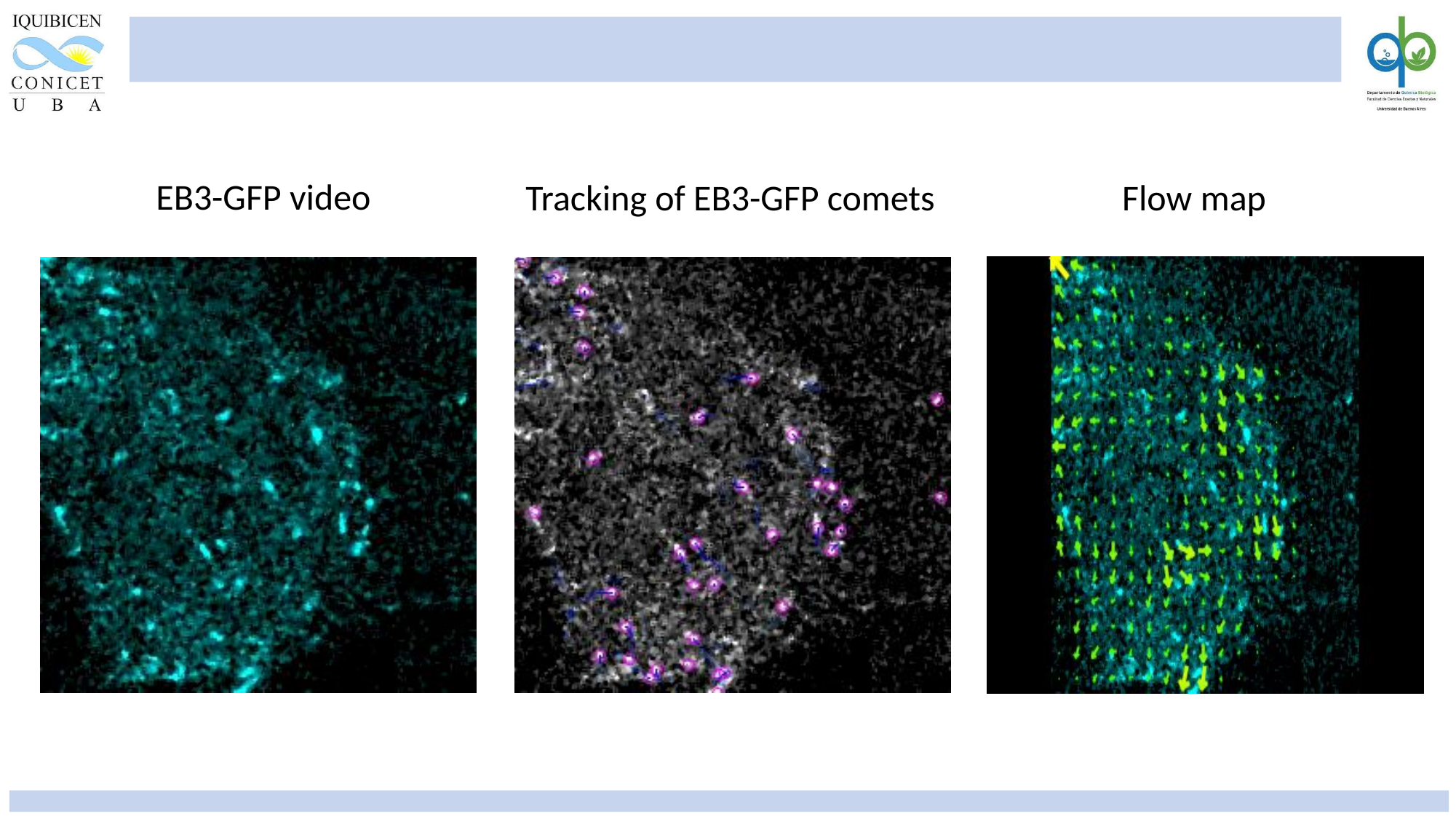

EB3-GFP video
Tracking of EB3-GFP comets
Flow map
