# Supplementary material for: Nucleus-cytoskeleton communication impacts on OCT4-chromatin interactions in embryonic stem cells
Source: BMC Biol. 2022 Jan 7;20:6. doi: 10.1186/s12915-021-01207-w (PMC8742348; doi:10.1186/s12915-021-01207-w)
Supplement: Supplementary file 8 — Additional file 8 Supplementary Fig. S2. Omics data analysis of vimentin expression in mouse embryo and different cell types. Data analysis of vimentin expression from microarray, RNA-seq and proteomics (as indicated in each panel) performed in Stemformatics data-mining platform. Bars represent mean ± SEM when corresponding. Full meta-data of analyzed datasets is available at Additional file 7: Supplementary Table S1. A The left panel shows the comparison between embryonic stem (ES) and epiblast-derived stem (EpiS) cells from different development stages of mouse embryo: Cavity (CAV, E5.5 – E6.0); Pre-primitive streak (PS; E6.0 – E6.5); Late Mid Streak (LMS; E6.75 – E 7.25); Late Streak (LS; E7.25 – E7.5); Early Bud (EB; E7.75) and Late Bud (LB; E8.0). The right panel shows data from different tissues during advanced embryo development. B Data from different stem cell types: ES cells, mesenchymal stem (MS) cells and multipotent adult progenitor (MAP) cells. C Data from ES cells differentiation experiments. The left panel shows vimentin mRNA levels from ES cells and neural progenitor (NP) cells. The right panel shows data from ES cells and ES cells-derived mesoderm cells, cardiac progenitors and cardiomyocytes. D Data obtained from ES cells during their differentiation to epiblast-like stem (EpiLS) and primordial germ cell-like (PGCL) cells. E Data from ES cells, mouse embryonic fibroblasts (MEF) and induced pluripotent stem (iPS) cells. F RNA-seq (left panel) and proteomic (right panel) data of ES cells, and MEF during their reprogramming to iPS cells. [file 12915_2021_1207_MOESM8_ESM.pptx]

## Slide 1
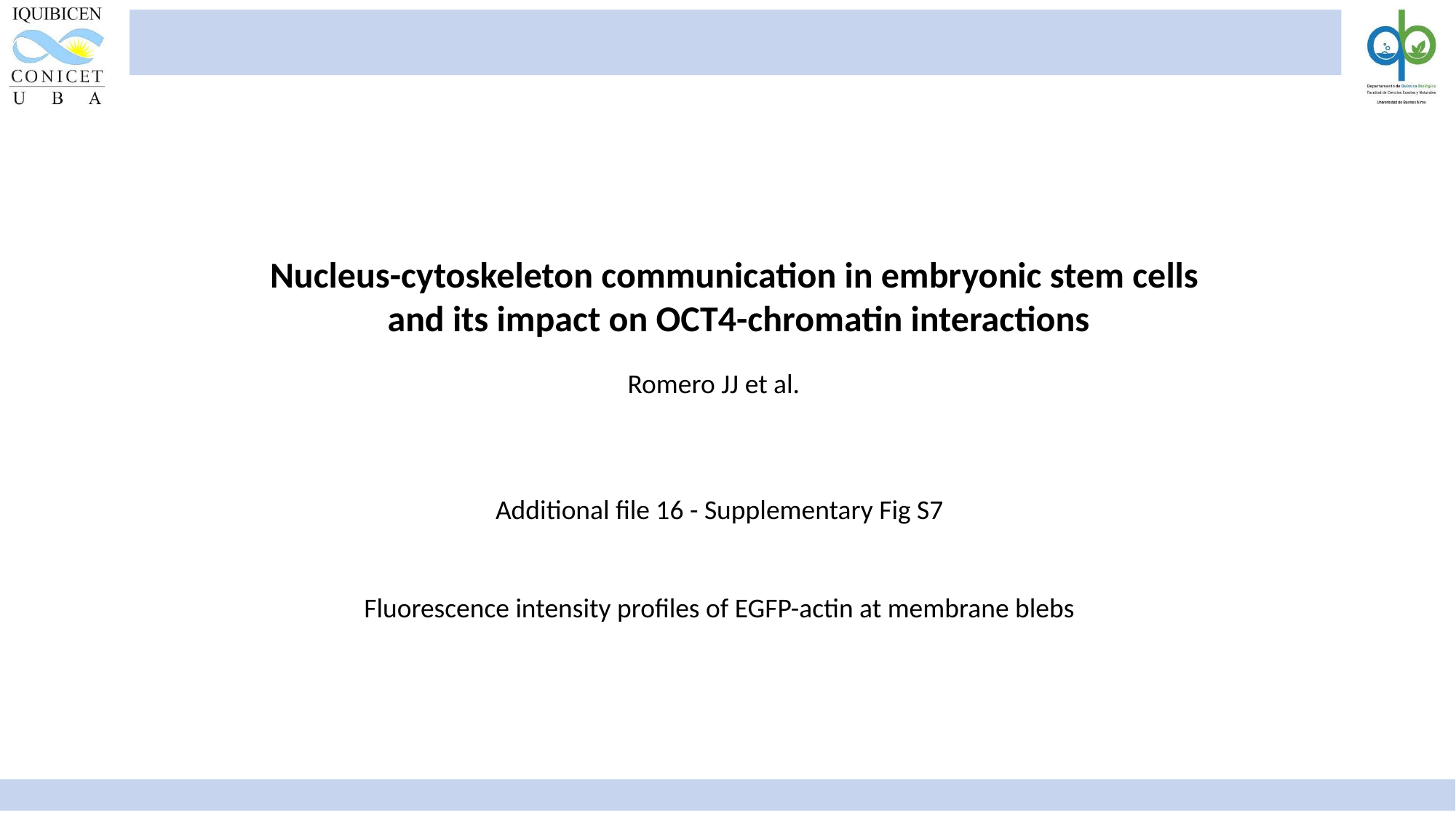

Nucleus-cytoskeleton communication in embryonic stem cells
and its impact on OCT4-chromatin interactions
Romero JJ et al.
Additional file 16 - Supplementary Fig S7
Fluorescence intensity profiles of EGFP-actin at membrane blebs

## Slide 2
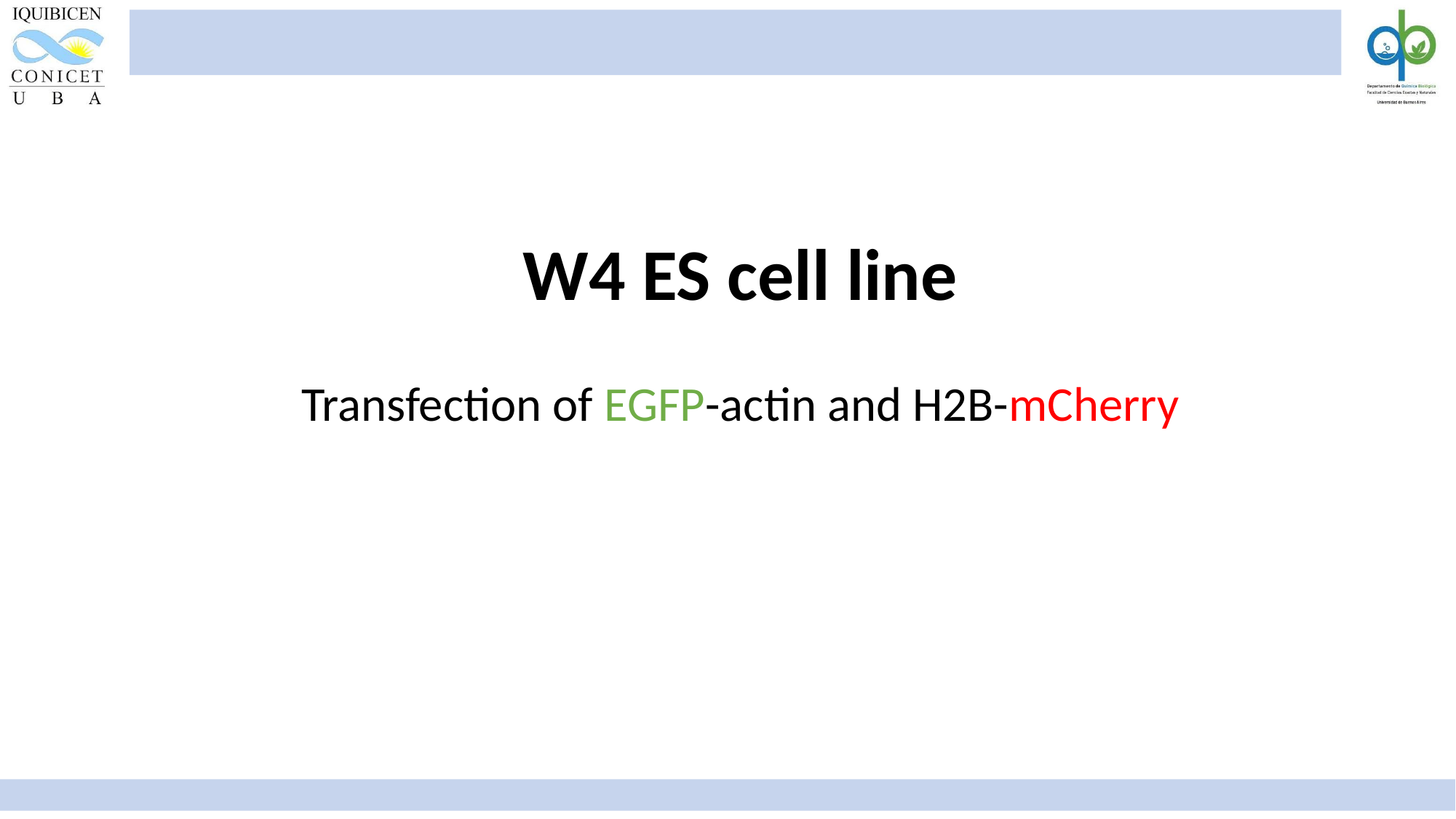

W4 ES cell line
Transfection of EGFP-actin and H2B-mCherry

## Slide 3
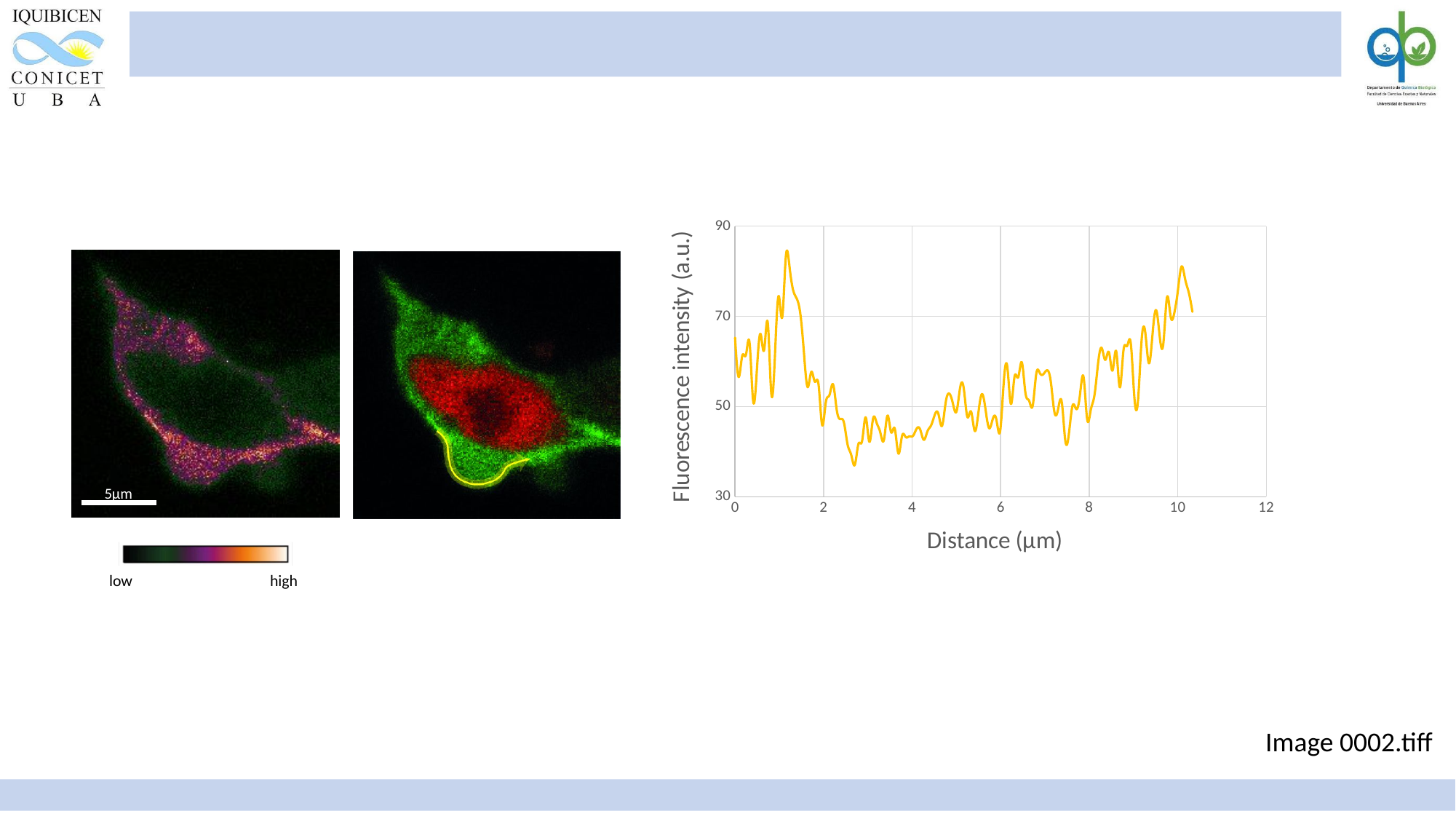

### Chart
| Category | Gray_Value |
|---|---|
5μm
low
high
Image 0002.tiff

## Slide 4
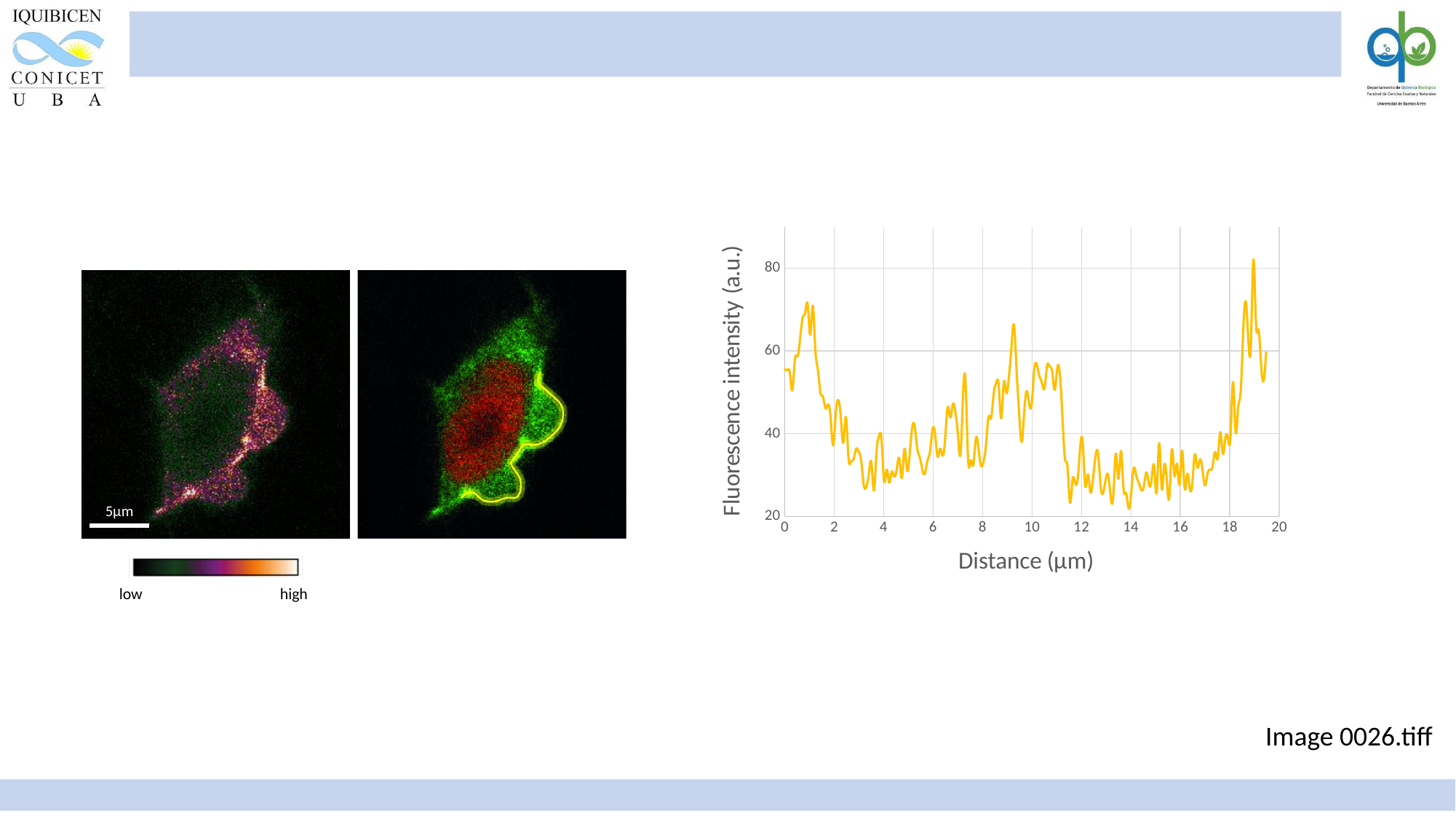

### Chart
| Category | Gray_Value |
|---|---|
5μm
low
high
Image 0026.tiff

## Slide 5
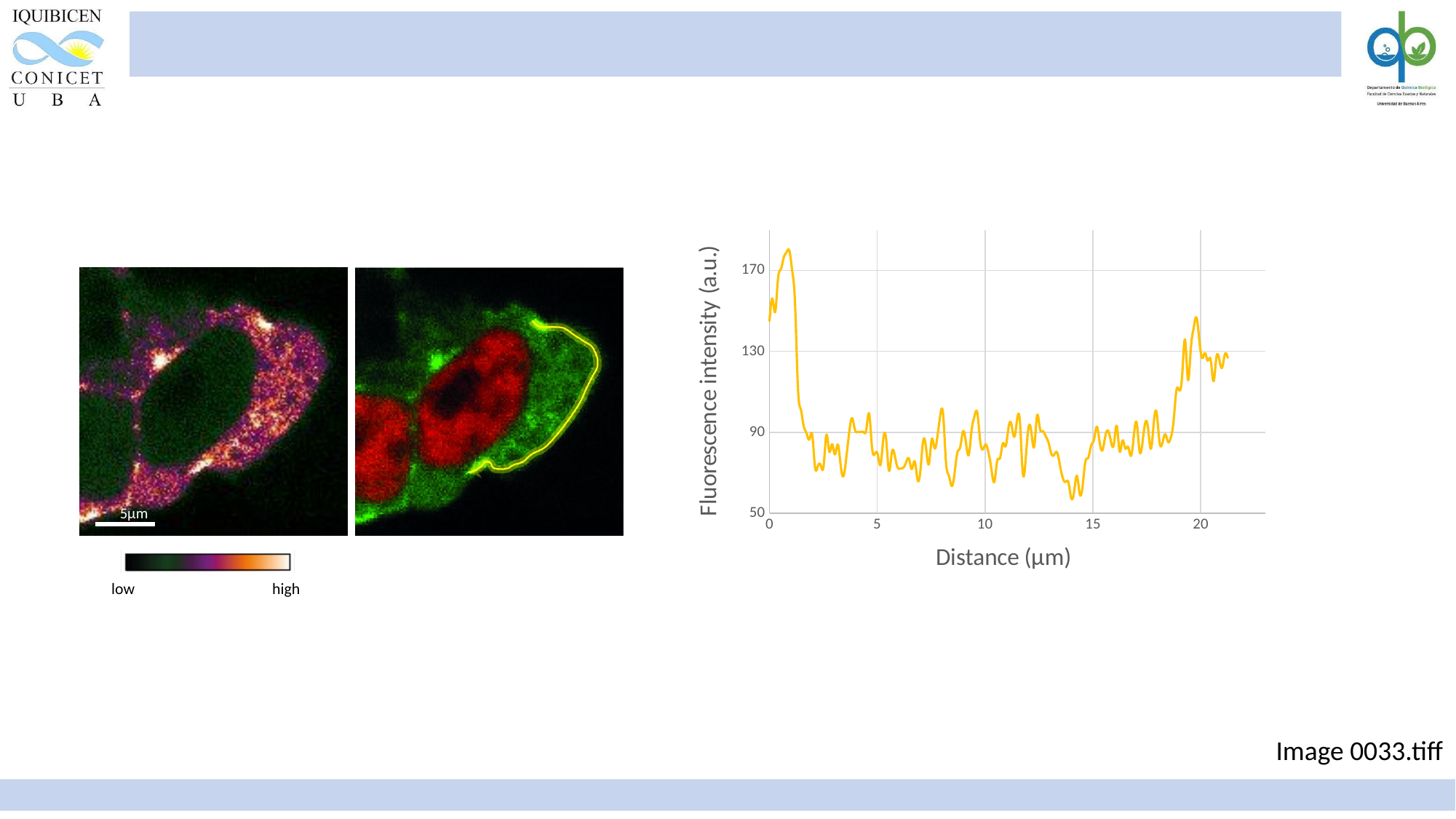

### Chart
| Category | Gray_Value |
|---|---|
5μm
low
high
Image 0033.tiff

## Slide 6
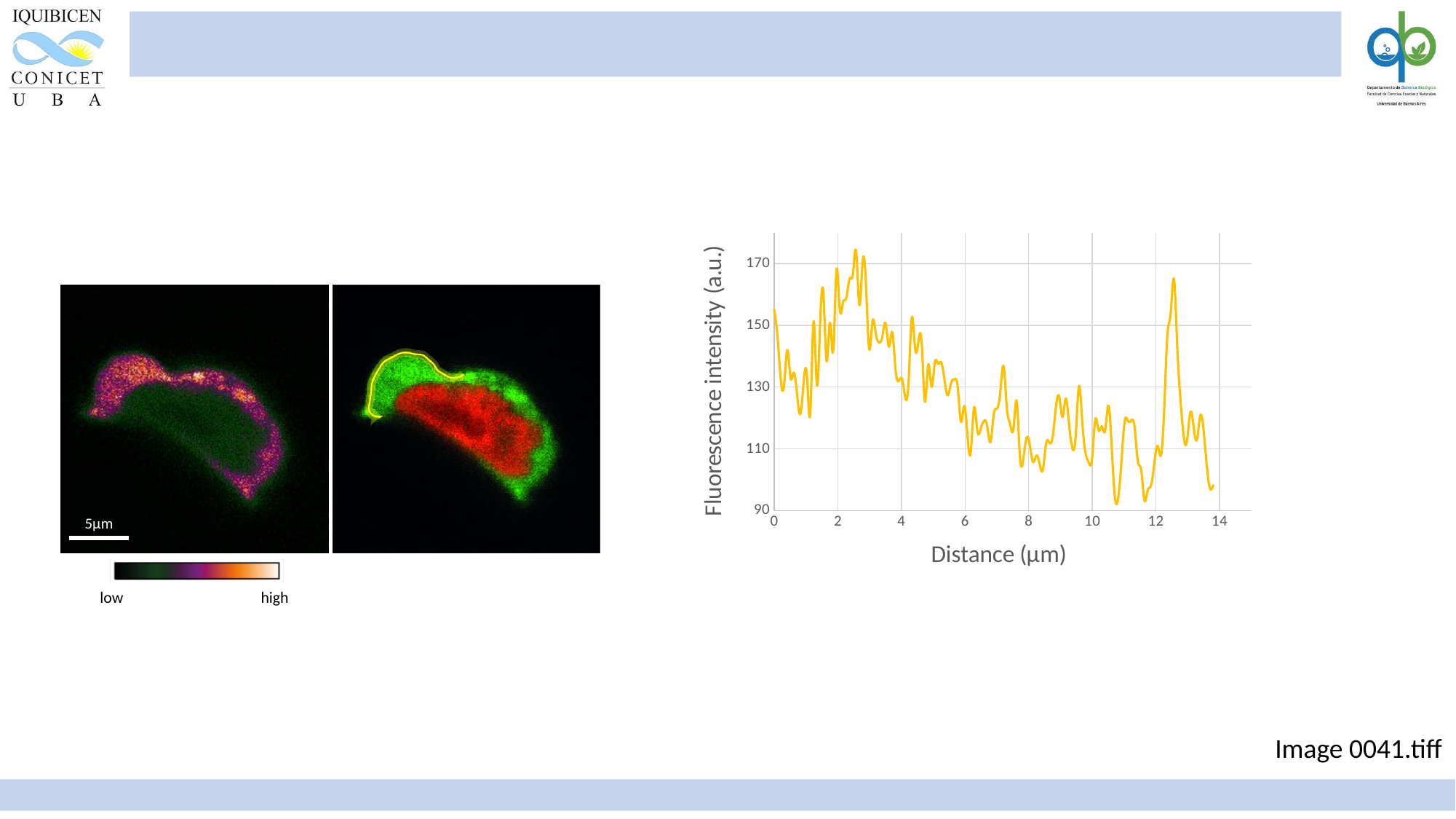

### Chart
| Category | Gray_Value |
|---|---|
5μm
low
high
Image 0041.tiff
