# Supplementary material for: Nucleus-cytoskeleton communication impacts on OCT4-chromatin interactions in embryonic stem cells
Source: BMC Biol. 2022 Jan 7;20:6. doi: 10.1186/s12915-021-01207-w (PMC8742348; doi:10.1186/s12915-021-01207-w)
Supplement: Supplementary file 14 — Additional file 14. Supplementary Fig. S6. Representative 3D images of ES cells expressing EMTB-3xGFP (green) and H2B-mCherry (red). Related to Fig. 1a. [file 12915_2021_1207_MOESM14_ESM.pptx]

## Slide 1
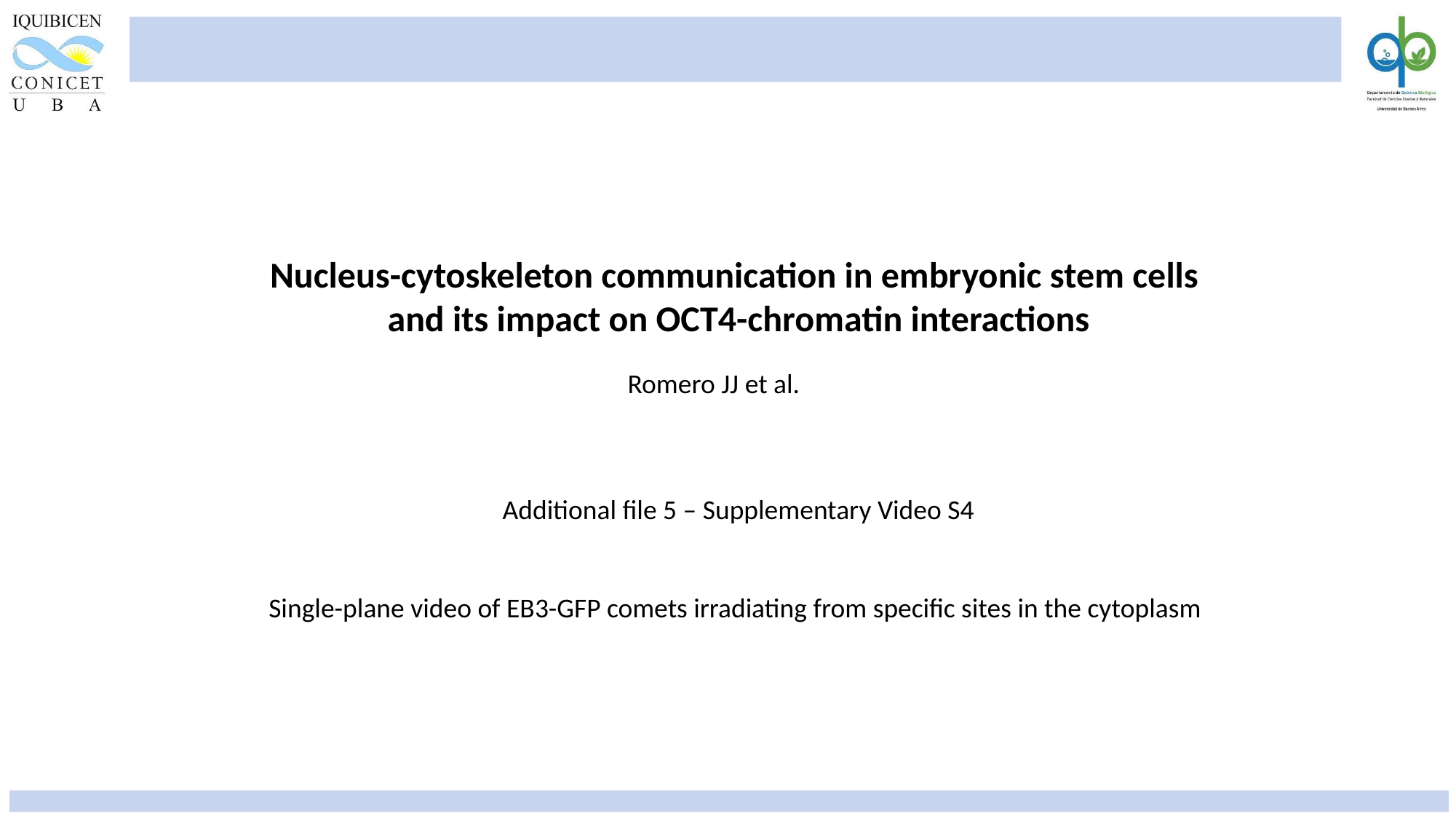

Nucleus-cytoskeleton communication in embryonic stem cells
and its impact on OCT4-chromatin interactions
Romero JJ et al.
Additional file 5 – Supplementary Video S4
Single-plane video of EB3-GFP comets irradiating from specific sites in the cytoplasm

## Slide 2
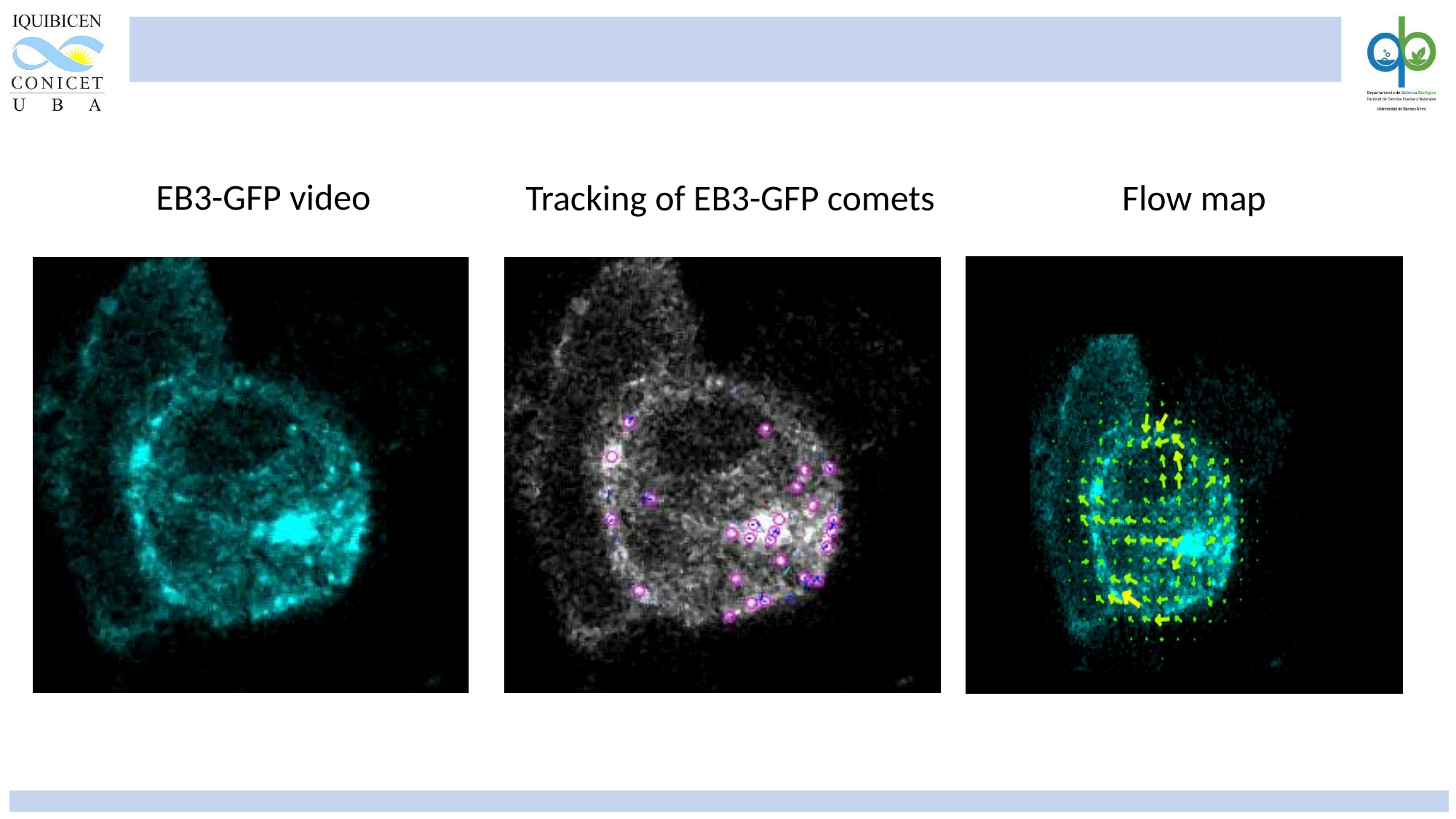

EB3-GFP video
Tracking of EB3-GFP comets
Flow map
