# Supplementary material for: Nucleus-cytoskeleton communication impacts on OCT4-chromatin interactions in embryonic stem cells
Source: BMC Biol. 2022 Jan 7;20:6. doi: 10.1186/s12915-021-01207-w (PMC8742348; doi:10.1186/s12915-021-01207-w)
Supplement: Supplementary file 16 — Additional file 16. Supplementary Fig. S7. Quantification of EGFP-actin fluorescence intensity along membrane blebs. Related to Fig. 2c and d. [file 12915_2021_1207_MOESM16_ESM.pdf]

Supplementary Table S1

	Reference	*Article*	*Specie*	*Platform*	*Approach*	*Database*	*Accession Number*
ES cells and reprogramming process	48	Proteome adaptation in cell reprogramming proceeds via distinct transcriptional networks	Mus musculus	Agilent 1200 HPLC (nanoLC) coupled to a Thermo LTQ-Orbitrap Velos (MS)	Proteomics	proteomeXchange (consortium)	PXD000413
ES cells-derived differentiation	49	Reconstitution of the mouse germ cell specification pathway in culture by pluripotent stem cells	Mus musculus	Affymetrix Mouse430_2 (GPL1261 and A-AFFY-45)	Expression Array	GEO (NCBI)	GSE30056
ES cells and reprogramming process	50	Genome-wide characterization of the routes to pluripotency	Mus musculus	Life Technologies SOLiD 5500xl NCBIM37 Gene + Transcript	RNA-Seq	Sequence Read Archive (NCBI)	SRP046744
Cells from developing embryo	51	Temporal Transcriptional Profiling of Somatic and Germ Cells Reveals Biased Lineage Priming of Sexual Fate in the Fetal Mouse Gonad	Mus musculus	Affymetrix MoGene-1_0-ST V1	Expression Array	GEO (NCBI)	GSE27715
Cells from developing embryo, ES cells /MEF/iPS cells	52	The Transcriptional and Functional Properties of Mouse Epiblast Stem Cells Resemble the Anterior Primitive Streak	Mus musculus	Illumina MouseWG-6 v2	Expression Array	GEO (NCBI)	GSE46227
ES cells-derived differentiation	53	SOX2 co-occupies distal enhancer elements with distinct POU factors in ESCs and NPCs to specify cell state	Mus musculus	Affimetrix Mouse430_2	Expression Array	GEO (NCBI)	GSE38850
Different stem cell types	54	Comparative transcriptome analysis of embryonic and adult stem cells with extended and limited differentiation capacity	Mus musculus	Affymetrix Mouse430_2	Expression Array	GEO (NCBI)	GSE6933
ES cells-derived differentiation	55	Dynamic and coordinated epigenetic regulation of developmental transitions in the cardiac lineage	Mus musculus	Illumina Genome Analyzer IIx RNASeq mm9 Gene TMM-RPKM (GPL11002)	RNA-Seq	GnomEx (Utah)	7R2

Supplementary Table S1. Related to Supplementary Fig. S2. Meta-analysis of microarray, RNA-seq and proteomic datasets analyzed in this work. Studies were selected from the Stemformatics web tool.
